# Author Correction: Past penguin colony responses to explosive volcanism on the Antarctic Peninsula

**DOI:** 10.1038/s41467-022-30657-1

**Published:** 2022-06-07

**Authors:** Stephen J. Roberts, Patrick Monien, Louise C. Foster, Julia Loftfield, Emma P. Hocking, Bernhard Schnetger, Emma J. Pearson, Steve Juggins, Peter Fretwell, Louise Ireland, Ryszard Ochyra, Anna R. Haworth, Claire S. Allen, Steven G. Moreton, Sarah J. Davies, Hans-Jürgen Brumsack, Michael J. Bentley, Dominic A. Hodgson

**Affiliations:** 1 British Antarctic Survey (BAS), Natural Environmental Research Council (NERC), High Cross, Madingley Road, Cambridge, CB3 0ET UK; 2 Institute for Chemistry and Biology of the Marine Environment (ICBM), Carl-von-Ossietzky-Str. 9–11, 26133 Oldenburg, Germany; 3 Department of Geosciences, Bremen University, Klagenfurter Str. 2–4, 28359 Bremen, Germany; 4 School of Geography, Politics and Sociology, Newcastle University, Newcastle-upon-Tyne, NE1 7RU UK; 5 Department of Geography, Northumbria University, Ellison Building, Newcastle-upon-Tyne, NE1 8ST UK; 6 Institute of Botany, Polish Academy of Sciences, ul. Lubicz 46, 31–512 Kraków, Poland; 7 School of Earth and Ocean Sciences, Cardiff University, Main Building, Park Place, Cardiff, CF10 3AT UK; 8 NERC Radiocarbon Facility (Environment), Scottish Enterprise Technology Park, Rankine Avenue, East Kilbride, Scotland G75 OQF UK; 9 Department of Geography and Earth Sciences, Aberystwyth University, Aberystwyth, SY23 3DB UK; 10 Department of Geography, Science Laboratories, Durham University, South Road, Durham, DH1 3LE UK

Correction to: *Nature Communications* 10.1038/ncomms14914, published online 11 April 2017.

The original version of this Article omitted a reference to previous work in ‘Hobbs, L.K., Gilbert, J.S., Lane, S.J. & Loughlin. S.C. Supplementary Case Study 3: Interactions of volcanic airfall and glaciers. In *Global Volcanic Hazards and Risk*’ (eds Loughlin, S.C., Sparks, S., Brown, S.K., Jenkins, S.F. & Vye-Brown, C.) 223–232 (*Cambridge Univ. Press*, 2015). This has been added as reference 71 at page 11 in the discussion: ‘The northern AP is defined as having a ‘moderate to extremely high likelihood of supraglacial airfall by active volcanoes’’. This has been corrected in the PDF and HTML versions of the Article.

