# Supplementary material for: Past penguin colony responses to explosive volcanism on the Antarctic Peninsula
Source: Nat Commun. 2017 Apr 11;8:14914. doi: 10.1038/ncomms14914 (PMC5394244; doi:10.1038/ncomms14914)
Supplement: Supplementary Information — Supplementary Notes, Supplementary Discussion, Supplementary Methods, Supplementary Figures, Supplementary Tables and Supplementary References [file ncomms14914-s1.pdf]

Supplementary Note 1

Geochemical evidence of past penguin colonies

Inorganic geochemistry has been widely used to determine penguin colonisation history of specific locations because penguin guano contains significantly higher concentrations of certain 'bio-elements', *e.g.* As, Ba, Ca, Cd, Cu, P, S, Se, Sr, and Zn[1] (Supplementary Table 1). Where bio-elements accumulate in undisturbed soil and lake sediment profiles[2,3] they can provide long term, *in-situ,* continuous and well-dated proxy-records of past changes in local penguin occupation[3]. In many respects, bio-element assemblages are ideal proxies of ornithogenic ('bird-formed') soil/sediment production. First, trace bio-elements are enriched in these phosphate phases and then immobilised in soils and sediment profiles during this substitution process. Second, when the relative proportions of penguin influenced soil/sediment and lithogenic material in overland runoff are stable, changes in bio-element concentrations in the lake sediments reflect variations in ornithogenic soil/sediment input rather than changing erosive rates[4]. Third, if a strong correlation exists between chemically different bio-elements, their concentrations reflect fluctuations in guano input rather than post-depositional processes or changes in provenance[5]. Previous studies around Antarctica have shown that ornithogenic soils and terrestrially-derived ornithogenic sediments (including lake sediments) formed in areas occupied by penguins have significantly higher concentrations of guano-related 'bio-elements' than most sedimentary deposits (Supplementary Table 1). For example, lake sediments from Hope Bay, Antarctic Peninsula were highly enriched in Ca, Cd, Cu, P, Sr, and Zn linked to a fluctuating input of guano into the lake[5]. Geochemical studies on soils of active penguin rookeries in the Seabee Hook area of Cape Hallett in northern Victoria Land, East Antarctica revealed a significant enrichment in elements, such as As, Ca, Cu, P, S, and Zn[6]. Similarly, in the Vestfold Hills higher concentrations of As, Cd, Cu, F, Mg, Ni, P, Se, Sr, TC TN, TS, and Zn were found in ornithogenic soils[2,7]. At Cape Bird and on Beaufort Island (Ross Sea region) ornithogenic sediments are typically enriched in As, Cu, Cd, Cu, P, TS, and Zn[1]. In the Larsemann Hills, dry mass accumulation ratios of P, Zn, Cu, Se, F, As, Cd, and Ni over Al have been used to reconstruct the historical seabird population in the catchment area of Mochou Lake[8].

Bio-element assemblages are immobile in (Antarctic) lake sediments[2,3,9] and guano input leads to the formation and preservation of stable phosphates, such as struvite ($Mg(NH_4)PO_4 \times 6\ H_2O$), leukoposphite, and, in particular, hydroxylapatite ($Ca_5(PO_4)_3(OH)$), which is one of the dominant compounds found in ornithogenic soils/sediments on King George Island[10]. The high correlation between Mg and P in ornithogenic soils from Vestfold Hills may indicate the presence of struvite[2], but on Ardley Island and, more specifically, in the Ardley Lake sediment record (ARD), Mg is most likely derived from the bedrock lithology (Fig. 3). The more positive correlation between Ca and P ($r=0.63$, $p<0.001$) in the ARD record and lack of a similar correlation in the YAN record suggests that hydroxylapatite is the main phosphate phase (Supplementary Tables 2, 3, Supplementary Fig. 6). During the precipitation of apatites, an exchange between Ca^{2+}, PO_4^{3-}, F^- and OH^- and elements such as Ag, Br, Ba, Cd, Cu, Cr, I, Na, Mg, Mo, Pb, S, Se, Sr, U, V, Y, and Zn is possible[11–14], coupled to microbial mediated degradation of solid phases[14]. Consequently, these trace bio-elements, most of which have naturally higher concentrations in penguin guano, are enriched in these phosphate phases and immobilised during this substitution process.

Geochemical shifts found in the Ardley Lake record are not related to marine or brackish to freshwater transitions since the combined assemblage of elements has only previously been found in association with guano-influenced deposits. Furthermore, no such

assemblage exists in the Yanou Lake record, which has a well-defined marine-brackish-freshwater transition[15]. In summary, we concluded, therefore, that simultaneous changes in bio-element concentrations in our lake sediments directly reflect the amount of ornithogenic soil and guano-influenced sediment present in the catchment area. Since both are proportionately linked to the number of penguins present in the catchment, we were able to reconstruct past changes in penguin population by converting the fraction of guano-influenced sediment in the ARD lake record into guano dry mass accumulation rates (see Methods for details).

Supplementary Note 2

Additional site information

Ardley Lake is the only large (c. 7,270 m^2), permanent closed-basin greater than 3 m deep on Ardley Island, and the only permanent depositional 'sink'. (Fig. 1d; Supplementary Fig. 2). With two prominent meltwater inflows at its western end and an overspill outflow at its retaining sill at c. 16 m above present sea level (a.p.s.l.), Ardley Lake is well-sited in the centre of the island to provide a long-term (mid-late Holocene) and continuous record of past changes in penguin occupation because it has remained above sea-level for the last c. 9 ka[15] (Fig. 1d). Sediments from ephemeral meltwater ponds on Ardley Island ('Lakes' Y2, Y4, G, AD3 and AD4; Fig. 1d) influenced by penguin guano are characterised by phases with enhanced concentrations of Ba, Ca, Cu, F, P, S, Se, Sr, Zn, and total carbon (TC), and a putative link between increased guano input during 'warmer' parts of the late Holocene has been established[3,16].The longest lake sediment guano-record obtained thus far from Ardley Island is a 3,000-year biogeochemical-record from 'Lake' Y2[3,17] (currently accessed by seals). 'Lake' Y4 (inaccessible to seals) contains a c. 1,500-year guano record[17,18] and the oldest penguin bones in raised beaches were deposited c. 2,500 years ago[19]. While important in establishing the longer-term presence of penguins on Ardley Island, our hydrological analysis of the 5 m resolution King George Island Digital Elevation Model (KGI-DEM) shows that Lakes Y2 and Y4 are small, ephemeral open basins with outflows or sub-surface drainage to the sea (Fig. 1d). The c. 8,500-year sediment record from Ardley Lake, has been used to constrain changes in relative sea level[15], but its potential as a penguin guano-record has not been explored.

Ardley Island was initially deglaciated c. 10,000 years ago[15], but recent studies of moss banks on the Ardley Island are limited to c. 500 years[20], and no ornithogenic soils/superficial terrestrial records older than 1,000 years BP have been found[21]. Older evidence of penguin occupation on Ardley Island (and elsewhere on the Peninsula) has come from lake sediment records (Supplementary Table 1). It is commonplace for lakes to preserve longer, more complete, and continuous sedimentary sequences than the superficial terrestrial sedimentary record, which can be patchy and prone to erosion. Ardley Island has an active periglacial environment with constant reworking, seasonal meltwater activity and trampling by penguins. Ardley Lake sediments preserve an undisturbed record of all events in the catchment over the last c. 8,500 years and is a continuous record of mid-late Holocene change on the island. Radiocarbon dated bone-collagen from juvenile (gentoo) penguin bones at several key points in the mid –late Holocene Ardley Lake sediment record also show that penguins laid eggs on Ardley Island from c. 5,040 cal a BP [5,240-4,860 cal a BP] onwards (Supplementary Table 2). We found evidence for a period of sustained increased sediment erosion in elevated dry mass accumulation rates and the deposition of coarser sediment into Ardley Lake between 6–4 ka (Fig. 4b, Supplementary Fig. 16b). This coincided with a period of more intense volcanic activity from Deception Island (T5 eruption series), which could have destabilised

the landscape, and possibly led to the erosion of much of the pre-4 ka BP terrestrial sedimentary record.

Yanou Lake (YAN) is a freshwater lake on the southeast of Fildes Peninsula, King George Island, located c. 1.3 km from Ardley Lake (Fig. 1d). It is underlain by the same basaltic-andesitic volcanic bedrock as Ardley Island, but, as for large parts of Fildes Peninsula, we found no evidence of past or present penguin occupation in its catchment. Yanou Lake is also a closed basin system, up to c. 5 m deep, with a main meltwater input stream at its northern end formed by overspill from the adjacent Gaoshan Lake located at c. 35 m a.p.s.l. (Fig. 1d). A lack of guano deposits in the YAN record therefore reflects the absence of penguins in both Yanou and Gaoshan lake catchments. Since its retaining sill is at c. 11 m a.p.s.l., Yanou Lake remained a shallow marine basin submerged below sea level until c. 6.5 ka BP following deglaciation, with basal sediments deposited in a glaciomarine environment c. 12–7.8 ka BP[15]. The marine embayment evolved into a shallow near-shore lagoonal basin, which persisted until isolation, c. 6.5 ka. In this study, the terrestrial 6.5–0 ka record acts as a control site for bio-geochemical inputs associated with the underlying geology, soils, tephra deposition and natural lake development. The absence of guano-influenced sediments in the YAN record, and the recently-published GDGT-temperature calibration curve specifically developed for the Antarctic and Sub-Antarctic lakes[22] enabled us to reconstruct the first quantitative, Glycerol Dialkyl Glycerol Tetraether (GDGT)-biomarker based mean summer air temperature (MSAT) record (YAN-GDGT-MSAT) from lake sediments on the Antarctic Peninsula (Fig. 4; Supplementary Fig. 16).

The balance between spring/summer open-water conditions indicated by changes in the diatom species assemblage in the new shelf-edge Anvers Trough marine sediment core diatom record (Fig. 1a; Supplementary Fig. 16) reflect changes in sea-ice extent and seasonality and provide the most relevant proxy of changes in the extent of NW-AP and SSI sea-ice edge area available to penguins. We included this new shelf-edge record, located between the mean modern-day (1981–2010 CE) Austral summer minimum (February) and winter maximum (September) sea-ice limits in the Bransfield Strait area (Fig. 1a), to avoid complications associated with fjord and inner-shelf sea-ice records that could be more impacted by terrestrial ice-discharge[23–26]. We compared changes in the new Anvers Shelf open water record with a local SSI sea-ice record from Maxwell Bay[27] to reconstructed MSAT changes in the YAN-GDGT record, the James Ross Island ice core (JRI) atmospheric temperature record[28] and the Palmer Deep sea surface temperature (SST) and 0–200 m temperature records (PD-SST)[24,26]. The PD-SST records are the most relevant temperature constraints available for mid-southern Peninsula penguin colonies and allowed us to examine possible cross-Peninsula temperature differences in parts of the early and mid Holocene not covered by the YAN-GDGT terrestrial temperature record.

Supplementary Note 3

NDVI mapping and penguins on Ardley Island

Vegetated areas on Fildes Peninsula and Ardley Island were mapped from a Normalised Difference Vegetation Index (NDVI) analysis of satellite data acquired on 4th November 2013 (Copyright DigitalGlobe catalog ID 1030010020C0C900, used under license by BAS) following established methods[29] (Fig. 1c). NDVI is the most commonly used vegetation classification ratio and gives an indication of verdancy of vegetation. The preliminary threshold values of 0.1–0.3 used to analyse the Fildes Peninsula/Ardley Island area were initially selected by calibrating the relationship between NDVI values from high-resolution imagery and ground truth data of vegetation assemblages around the Ryder Bay region, Antarctic Peninsula[29]. Although increased moss and lichen density and species richness

has been linked to fertilisation by small-medium penguin colonies across the Antarctic Peninsula[30], dense vegetation and high NDVI areas do not always reflect fertilisation by guano. Indeed, the most intensely vegetated area on Ardley Island are moss banks on northern slopes of the island. While occasionally frequented by passing penguins, these areas are outside modern-day penguin nesting areas. The greater intensity of vegetation is likely due to a favourable slope-aspect, its relatively sheltered location more protected from storms that pass through the Bransfield Strait and because it is sufficiently far from present day nesting areas to not be trampled or poisoned by toxic compounds in penguin guano. Other areas of note on Fildes Peninsula with elevated NDVI values are (Fig. 1c): 1) the valley on the far side of Marsh Airport that consists of a wide expanse of organic-rich mud, the lower parts of which form a large tidally-influenced seal wallow; 2) precipitous offshore islands where sea-bird colonies nest in the cliffs where intense vegetation is possibly related to fertilisation by their guano; 3) moss patches and areas of extensive lichen development in the interstitial raised beach on the north-facing shoreline in front of Yanou Lake where no evidence for present or former penguin colonies has been found.

Gentoo penguins on Ardley Island are known to move between nesting areas on a regular basis due to overcrowding and guano toxicity build-up[31]. Present day nesting areas, outlined in Fig. 1d, have a more patchy distribution, composed of small groups of individual nests. New multi-annual penguin nest distribution maps for Ardley Island are in progress (Mustafa, pers. comm.), but our (single summer-season) observations showed that the present day Ardley penguin colony could be encroaching into the area to the north of Lake Y4 (Fig. 1d), and possibly the Ardley Lake catchment. Soil samples from modern-day nesting areas on the western side of Ardley Island (Fig. 1d) have exceptionally elevated phosphine emission and production levels[31], while higher-than normal organically-derived phosphine and phosphatase activity in soils from elsewhere on Ardley Island has also been linked to penguin-guano deposition[31]. NDVI analysis corresponds, in part, to this pattern of intensive vegetation on inland Ardley Island, which is focussed away from largely de-vegetated active nesting areas at the western coastal margins. Therefore, we link some such instances of NDVI enhancement to fertilisation by penguins. The densest vegetation around Ardley Lake (from NDVI analysis) relates to small moss patches, not individual lichens as these are generally too small to be detected at the scale of analysis undertaken. The present-day nesting density of other seabirds (*e.g.*, skuas) is low, in general, on the island (we observed two nesting pairs in the Ardley Lake catchment) and, although Ardley is low-lying island (<c. 70 m a.p.s.l.), its inland terrain is mostly inaccessible to seals. We also note that penguin-guano contribution to soil-phosphorous is more than three times greater than that of seal-guano (and approximately five times higher than background soils) and phosphate levels in modern nesting areas are significantly elevated[31].

Hydrological analysis of the King George Island 5 m resolution Digital Elevation Model highlighted that meltwater ponds ‘Lakes’ G and Y5 have well-defined hydrological systems, each with independent catchment areas, but both are ephemeral and open systems, outflowing to the sea (Fig. 1d). This is also the case for ‘Lake’ Y4, but its catchment area is poorly defined by the 5 m resolution DEM analysis. ‘Lake’ Y2, on the eastern side of the island is fed from two catchment areas, but is also a very shallow ephemeral basin (<3 m deep) that seasonally dries up due to evaporation and sub-surface drainage to the sea under its retaining c. 11 m a.s.l. raised beach ridges (Fig. 1d).

Supplementary Note 4

Sedimentology of ARD and YAN records

The ARD and YAN sediment records were extracted from the most stable (non-moated) parts of ice-cover, as close as possible to the deepest point of the lake, which was determined by echo-sounded bathymetric readings taken every metre along bisecting fixed ropes with positional fixes using a Garmin handheld GPS (Supplementary Fig. 2). A square-rod Livingston piston corer[32] was used to extract five overlapping cores (ARD 1A–1E) from a core site at the depositional centre of Ardley Lake at water depths of 4.85–5.19 m (Fig. 1e). These were combined into one master core (ARD) with a total composite sediment depth of 359 cm (Supplementary Figs. 3–8). In addition, a 10 cm surface sediment core (ARD-SUR) was taken at the same location using a 50 cm long Russian corer, and surface transect cores were taken. At Yanou Lake, our control site, a UWITEC piston corer was used to extract three c. 1.9 m long overlapping cores (YAN 8A, YAN 8B, YAN 9B) from the deepest part of the northern basin, with a water depth of 4.9 m. Consolidated surface sediments were recovered using a 50 cm long Russian corer, which was able to penetrate the c. 3-5 cm thick living moss layer that carpets the surface of Yanou Lake. Four overlapping cores (YAN 8A-1, 8A-2, 9B-1, and 8B-2) were combined into a master core (YAN) with a total sediment depth of 355 cm composite depth (Supplementary Fig. 9). No evidence of slumping was found in the ARD and YAN sedimentary records.

In the Ardley Lake sediment record, seven major lithological units were visually identified. These broadly coincided with 13 significant geochemical zones, five of which have weighted mean $F_{o.sed.}$ values greater than 10% and are referred to as guano-phases GP-1 to GP-5 (Fig. 4, Table 1; Supplementary Figs. 3, 7, 8). Overall, the sediments were alternating olive-grey to black organic mud layers and fine laminated grey and black organic silt, interspersed with fine volcanic ash layers associated with eruptions from Deception Island (Supplementary Fig. 3). The basal zone of the core (333–359 cm) was characterised by coarse black tephra, associated with a more explosive T7(8?) eruption, capping a light grey clay deposit with medium-coarse sand and matrix-supported gravel dominated by >8 mm clasts (Fig. 3).

The five lithological units visually identified in the Yanou Lake record were refined into eight geochemically significant zones (Supplementary Figs. 9–11). Geochemical Zones 1 and 2 graded from glaciomarine diamicton into black lagoonal muds, with the basal gravel-rich diamicton overlain by fine sediments, containing shallow marine diatoms, deposited when Yanou Lake was still below sea level. Zone 3 is an airfall and reworked volcanic tephra deposit associated with the T7 Deception Island eruption. The short-lived phase of terrestrial lagoonal sedimentation at the base of Zone 2 and the brief return shallow marine conditions in Zone 4 has been linked to a stalled phase of deglaciation at this location during the early Holocene[15], but the re-establishment of marine-brackish conditions after the T7 eruption could be evidence of a minor tsunami or marine flooding of the low-lying nearshore basin which caused a temporary shift in basin ecology. Alternatively, the return to more brackish conditions in Zone 4 could reflect a period of post-T5-eruption catchment destabilisation, which coincided with increased Peninsula-wide storminess[15,26] and led to a greater input of wind-driven sea spray into Ardley Lake (Diatom Zone D2 in Supplementary Fig. 3; Supplementary Fig. 7, 9, 10, 12, 14, 16). In the YAN record, Zone 5 was characterised by fresh-brackish diatoms and composed of fine terrestrial sediments, prominent moss layers and T6 tephra deposits. Geochemical Zone 6 is a c. 130 cm thick deposit composed of airfall and reworked volcanic ash and interbedded silty-clay sediment. The airfall tephra deposits broadly correspond to sand grain-size layers devoid of diatoms (Supplementary Figs. 9–17). Zones 7 and 8 were composed of fine terrestrial sediments, tephra and moss layers, and we link their fresh-

brackish diatom composition to sea-spray inputs associated with Yanou Lake's proximity to the coast.

Supplementary Note 5

Chronology: Calibration and Radiocarbon Reservoir Effects

In general, lacustrine algae ages are regarded as 'more reliable' than bulk sediment and bone-collagen ages because they have a well-defined terrestrial carbon source, which, in a shallow lake environment, is more in equilibrium with atmospheric carbon (see Supplementary Note 6). In both records, calibrated ^{14}C ages of the lacustrine and terrestrial sediment are in, or close to, chronological order with respect to sediment depth. For lacustrine sediments, radiocarbon ages from living and near surface *Drepanocladus longifolius (Mitt.) Paris* species of aquatic moss in both Ardley Lake and Yanou Lake records both returned post-bomb, modern ages indicating a minimal modern day terrestrial lake reservoir effect exists for both lakes.

Older than modelled-age outliers identified during the first phase of age-depth modelling for the ARD record (ARD-M1), run without bone-collagen ages included, were most likely due to re-working of older organic material from the catchment or suggested that a marine reservoir correction needed to be applied to bulk sediment ages from elevated guano zones whose carbon was most likely derived from a marine source (Supplementary Fig. 6). These outliers had minimal influence on the post 3.5 ka ARD age-depth profile in the five age-depth models, ARD-M1 to ARD-M5, developed for the ARD record (see Supplementary Note 7). Model runs ARD-M2 to ARD-M5 were based on the good agreement between the bone-collagen data from 289–291 cm and a lacustrine algae sample from a similar depth (281–282 cm) and lacustrine algae and bulk sediment ages at 252–254 cm depth, outside of a guano-influenced part of the Ardley Lake record. However, further up-core, two bone-collagen calibrated ages from within guano phase GP-4 were significantly (>95% confidence interval maximum-minimum age range) younger (by c. 700 years) than their paired bulk sediment ages (Supplementary Fig. 3, Table 2). We rule out modern or bacterial contamination as the cause of the younger bone-collagen ages since all bones were treated in the same manner (dried/stored frozen) and it would be highly unlikely that both were contaminated to the same extent. Therefore, in the final ARD-M5 age-depth model, we included bone-collagen ages and also applied a local MRE ΔR value of 700 ± 50 years (Total MRE=1,100 ± 50 years) to bone-collagen and guano-influenced bulk sediment samples. This created a better-constrained age-depth model compared to the ARD-M1, but a younger, 8–3.5 cal ka BP age depth profile (see Supplementary Note 7 and Supplementary Figs. 4, 5 for more details). We are justified in applying a marine reservoir correction to bulk lake sediment age data in the guano-phases because bulk sediment organic carbon (%C) is strongly correlated with $\%F_{o.sed.}$ (n=137; PCC=0.86, R^2=0.75; RMSE=7.43; p<0.0001) (Supplementary Figs. 6a–d).

For marine sediments, the scale of the marine reservoir effect (MRE) on the Antarctic Peninsula is still debated and several values have been used[33]. For consistency, we have recalibrated and updated all our previously published lake sediment ages[15,34,35] and sub-fossil penguin age data[36–38] using the latest 2013 calibration curves. For the marine-influenced sections of the YAN record, we recalibrated the published MARINE09 calibrated ages[15] with the MARINE13 calibration curve[39,15]. There were some differences to previously published results[15] using a 100% marine carbon source for all marine-influenced sediments from 210–355 cm depth. We did not include terrestrial-marine partitioning (*e.g.*, 50% marine: 50% terrestrial) used previously[15] as we now consider that the partition could vary in a non-systematic manner through time. There were also no

significant differences between using a local MRE ΔR value of 664 ± 10 years (Total MRE=1,064 ± 10 years minus the global marine reservoir of 400 years)[15], and using a local MRE ΔR value of 700 ± 50 years (Total MRE=1,100 ± 50 years) most commonly used in compilations of published penguin bone and eggshell ages[21,40] (Supplementary Table 2).

Total marine reservoir offsets in the Antarctic can vary from more than c. 1,000 to as much as 6,000 years[41] depending on the depth at which the carbon is derived and stored, *i.e.*, how long it has been isolated from interaction with the atmosphere. The high protein diet of penguins can also influence bone-collagen isotope ratios, while a component of their ^{14}C radiocarbon is derived from respiration. Although entirely land-based, juvenile gentoo penguins receive a regurgitated marine-based diet and modern studies show that their diet is largely derived from relatively short and shallow foraging trips by their parents rather than lengthy, deep water diving[42]. Consequently, a comparatively small MRE might be expected, but there is a general lack of 'modern-day' radiocarbon age comparison data because all penguin bones collected from Antarctica after 1950 are affected by the 'Bomb-carbon' effect, which invalidates post 1950 CE ages. A ΔR value of 880 ± 50 years has been used[34] for marine sediments in the Beak Island Lake records based on the nearest measurements of the pre-1950 CE marine reservoir effect at Hope Bay, which consists of penguin bones from animals consumed at a refuge constructed during the unplanned winter of Dr Otto Nordensk.öld's 1903 Expedition radiocarbon dated at 1,280 ± 50 ^{14}C a BP (LU3101)[43]. The lower ΔR value of 700 ± 50 years used elsewhere[21,40] is justified for the South Shetland Islands as this more closely matches several modern day surface water ages from several sites in Maxwell Bay (weighted mean age = 1,064 ± 10 radiocarbon years[27], Wellner, pers. comm.; Supplementary Figs. 3, 7, Supplementary Table 2). We also note the strong coherence between lacustrine algae and bone-collagen calibrated ages when using a total MRE of 1,100 ± 50 years (ΔR = 700 ± 50 years[21,40]) for the ARD record.

As atmospheric-marine partitioning has not previously been taken into account when calibrating penguin-derived radiocarbon ages, we tested the effect of partitioning carbon between atmospheric and marine sources while calibrating penguin bone-collagen age data. As our penguin bones are juvenile, we did not consider additional partitioning effects that might relate to life-stage (*i.e.*, adult vs juvenile). We investigated the broad-scale partitioning effect using marine: atmospheric ratios of 100%, 75% and 50% and a range of local MRE ΔR values of 0 (highly unlikely), 664 ± 10 years[15], 700 ± 50 years[21] (most likely), and 900 ± 100 years[44] (most commonly-used in AP published papers). In summary, results show that both smaller marine reservoir corrections and increased proportions of atmospherically-derived carbon lead to older calibrated radiocarbon age ranges and mean values.

To summarise, the ΔR = 700 ± 50 years value used in this study encompasses: 1) the ΔR value of 664 ± 10 years applied to marine and marine influenced sediments (including YAN marine sediments) used perviously[15] 2) the ΔR value of 791 ± 121 years[45]. This ΔR value is smaller than the ΔR value of 900 ± 100 years (total MRE = 1,300 years)[44,46], and larger ΔR values[33] used elsewhere, but justified since the marine carbon present in our lake cores is more likely to have been from a shallow water sources, more in equilibrium with atmospheric carbon than deep water carbon sources. While some ice-proximal and periglacially active lakes from other islands in the SSI archipelago require a large lake reservoir effect[47], our modern surface age data from aquatic mosses from the ARD and YAN records show that no significant (>100 years) lake reservoir effect exists. Since ARD and YAN lakes have been located far from active glaciation on KGI for most of the

Holocene following rapid early Holocene retreat of the KGI ice cap[15] and due to the volcanic nature of the bedrock on this part of the Fildes Peninsula, we do not consider our terrestrial or shallow marine sediments to be significantly influenced by old terrestrially-derived carbon stored on land or retained within glacial ice.

Supplementary Note 6

Chronology: Penguin bones and radiocarbon dating

Terrestrial macrofossils and sub-fossils provide a well-defined carbon sources, but are often rarely well-preserved, and therefore extremely valuable if found in Antarctic lake sediment records. A long-standing consensus is that bones and bone-derived materials (*e.g.*, collagen) produce less reliable radiocarbon ages than single-species terrestrial and shallow aquatic mosses and other terrestrially derived specific-source organic carbon (*e.g.*, lacustrine algae). In this and previous studies mosses and terrestrial algae have produced near-zero radiocarbon ages when actively growing in surface sediments[48]. In contrast, penguin and whale bones sampled in terrestrial raised beach sedimentary sequences show wide scatter of radiocarbon ages compared to well-defined horizons in lacustrine deposits[15,34].

Before producing the final age-depth model, ARD-M5, for Ardley Lake, we first examined the influence of bone morphology, weathering, carbon and nitrogen isotope data on bone-collagen ages. In summary, the $\delta^{13}C$ bone-collagen data indicates that the penguins had a fatty (marine) diet (as would be expected) but offset $\delta^{13}C$ and $\delta^{15}N$ values indicate there could have been a degree of stress in their diet, possibly malnourishment, when compared to ‘normal’ penguin isotope ratios (Supplementary Fig. 6e). Our juvenile gentoo penguins $\delta^{13}C$ values of -19.8 to -22.5 ppm are enriched compared to isotopic values obtained from eggshells from the Antarctic Peninsula (Supplementary Fig. 6e Supplementary Table 2)[21,49]. However, gentoo penguins foraging in an enclosed bay have more enriched $\delta^{13}C$ values of -15.9 ppm, while King penguins foraging in open ocean have more depleted values of -22.3 ppm[50]. In general, enriched isotopic carbon values between -19 to -22 are indicative of inshore open sea to offshore foraging[50], which is consistent with a combination of short/shallow foraging during the breeding season, and evidence for a sustained period of increased spring/summer shelf-edge open water that existed when most of bones were deposited in the core (Fig. 5). $\delta^{15}N$ bone-collagen values of c. 8–9 ppm are within error of data from gentoo penguin eggshells from the Antarctic Peninsula (Supplementary Fig. 6e)[21,49] and, more generally, $\delta^{15}N$ values of terrestrial plants and animals (6–10 ppm). Lower $\delta^{15}N$ values in some marine-consumers can be indicative of an excessively fatty diet, but, paradoxically for penguins, can indicate a diet lacking in fish or higher trophic organisms. In general, fish have $\delta^{15}N$ values of 9.6–14.7 ppm and $\delta^{13}C$ values of 20.6–14.6 ppm[50]. Consequently, penguin species that are predominantly fish-eaters or species that favour a more varied diet when food is not scarce (*e.g.*, gentoo penguins) have generally elevated $\delta^{15}N$ values. Some species of penguins have been found to have $\delta^{15}N$ values averaging c. $\delta^{15}N$ = 13 and $\delta^{13}C$ = 19. Therefore, the lower $\delta^{15}N$ values obtained from the ARD record during radiocarbon dating analysis of bone-collagen imply a fatty diet consisting of mostly krill or lower trophic organisms, but this apparent lack of trophic variety might also indicate a degree of dietary stress.

Supplementary Note 7:

Chronology: Age-depth modelling

Overall, for the ARD record, all age-depth models ARD-M1 to ARD-M5 produce similar minimum-maximum 95% confidence basal age ranges of 8,410–9,230 cal a BP, with an average weighted mean basal age: 8,730 ± 380 cal a BP (95% confidence error). The first

age-depth model, ARD-M1 assumed that all deposits had a terrestrial carbon source. This produced broadly linear sedimentation rates of 7–120 cm ka^{-1}, with significantly higher values >2,000 cm ka^{-1} focussed on c. 5 cal ka BP (159–282 cm) laminated sediments where no evidence for slumping exists. We linked this to a phase of increased catchment destabilisation and erosion associated with a phase of increased volcanic activity from Deception Island between c. 5.5–4.8 cal ka BP[28,34,51] (see also Supplementary Table 5). However, the ARD-M1 model produced a comparatively poor match to some individual calibrated ages, in particular, bulk sediment radiocarbon ages within late Holocene guano-phases (Supplementary Figs. 6a–d, 13, 14). To rectify this, we undertook further age-depth modelling, which included newly-available bone-collagen age data. In models ARD-M4 and M5, shown in Supplementary Figure 5, we applied a 1,100 year total marine reservoir effect (MRE) ($\Delta R = 700 \pm 50$ + the global MRE of 400 years[40]) to penguin-bone collagen ages and guano-influenced phase bulk sediments (see Supplementary Note 6 for details; Fig. 3; Supplementary Fig. 3; Supplementary Table 2). The final age-depth model ARD-M5 used in this study applied a MARINE13 calibration and a local MRE ΔR value of 700 ± 50 years to bone-collagen radiocarbon ages and bulk sediment age data from guano-influenced phases GP-1, -4 and -5.

Setup conditions and results for model runs ARD-M1, ARD-M4 and ARD-M5 are summarised in Supplementary Figure 5. Overall, all age-depth models produce similar minimum-maximum 95% confidence basal age ranges of 8,410–9,230 cal a BP, with an average weighted mean basal age: $8{,}730 \pm 380$ cal a BP (95% confidence error). We finalised the ARD-M5 age-depth model by using an initial acc. mean (mean accumulation rate) setting of 50 a cm^{-1} (rather than the record average of 20 a cm^{-1} used in ARD-M1; Supplementary Fig. 5), which caused the ARD-M5 model to pass through the oldest measured sample age in the record (age 18). Since the BACON program adjusts the accumulation rate for each 5 cm segment, using this prior setting increased confidence in the age-depth model to represent the as-measured basal age data. Moreover, the age-depth model ARD-M5 produced the best fit to the measured radiocarbon ages and has the lowest mean 95% minimum-maximum age confidence interval = 410 years (compared to >1,000 years for ARD-M1). Age model ARD-M5 excluded just one calibrated age as an outlier at 158–159 cm depth (no. 11 in Supplementary Fig. 11), a bulk-sediment age from a non-guano influenced zone, which could have been affected by reworking of older carbon after the T4 eruption. Its bulk $\delta^{13}C$ value of -25.9‰ is as depleted as guano-phase bulk sediment carbon isotope data and the diatom ecology in this part of the core is >90% brackish, both implying a substantial marine influence. Therefore, to provide two end-member scenarios for sample no. 11, we also calibrated it assuming a 100% marine carbon source with the MARINE13 calibration curve and $\Delta R = 700 \pm 50$ years (Supplementary Fig. 11; Supplementary Table 2). This produced a calibrated mean age and 95% confidence interval age range that was more consistent with ARD-M5 age-depth model (Supplementary Fig. 1: labelled 11b).

As well providing the best fit to measured sample data, ARD-M5 also produces the best stratigraphically-constrained age agreement of visible tephra deposits with the independently dated YAN record and other well-dated records from across the northern Antarctic Peninsula (*e.g.*, Beak Island[34], James Ross Island[28], Livingston Island[47,52], and marine cores from the Bransfield Strait[51]) (Fig. 5, Supplementary Fig. 16, Supplementary Table 3). In summary, all age depth models, YAN-M1 to YAN-M4, produced the same result between 45–0 cm (c. 6-0 cal ka BP), and there no age reversals in the terrestrial c. 6-0 cal ka BP record (Supplementary Fig. 9). Since the same species of moss in the near surface sediments return modern ages, we consider the terrestrial record from Yanou Lake to be as reliable as is possible to achieve for the Antarctic Peninsula region. Further

downcore, the large mid Holocene T7 and T5 eruptions and the marine-terrestrial transition create a more complex age-depth profile in the YAN record. While the ages of T4 and T6 eruption events are well-constrained by terrestrial aquatic moss layer ages, we were not able to date samples between 190–32.5 cm within the c. 5,000-year airfall and reworked T5 tephra deposit (Supplementary Fig. 10) because no terrestrial macrofossils present and carbon content of sediment in this part of the core is close to zero. Age-depth model YAN-M1 was produced by allowing the Bayesian MCMC BACON analysis to run unconstrained and with a total MRE of 1,064 ± 10 years[15] applied to ages from the marine section of the core below 210 cm depth. The setup for YAN-M2 was the same as for YAN-M1, but we assumed that the tephra and reworked tephra was deposited instantaneously between 190–45 cm. Since the bounding moss ages made the YAN-M2 scenario unrealistic and the ages of the T6–T4 ash layers deposited between c. 6,000–4,200 cal a BP were similar in the ARD-M5 and YAN-M2 age depth models, for YAN-M4, we refined the chronology of Zone 6, we applied ARD-M5 weighted mean calibrated ages for T5a–c tephra deposits between 190–40 cm depth and applied a MRE correction of 1,100 ± 50 (ΔR = 700 ± 50) to all marine sediment ages to enable direct comparisons with the ARD and other penguin records. All YAN age-depth models have similarly good fits to measured radiocarbon data, but we use the YAN-M4 age-depth model as it is the most comprehensive single run composite age-depth model.

Supplementary Note 8

Penguin Population Modelling: additional information

Population modelling and area-required calculations described in the Methods sections were underpinned by analysis of the KGI-DEM and published empirical evidence from penguin studies. First, we undertook hydrological analysis using the 5 m resolution Scientific Committee on Antarctic Research (SCAR), King George Island GIS (KGI-GIS) Digital Elevation Model (KGI-DEM)[53], and field bathymetric data collected during lake coring on Fildes Peninsula to obtain key input parameters for population modelling (*e.g.*, lake and lake catchment area data, lake and landscape slope data, flow and discharge routes and rates). Since many of the lake outlines and watercourses were inaccurate, we updated and improved the KGI-DEM for Ardley Island in ARC-GIS using a new multi-spectral satellite imagery acquired on 4th November 2013 (Copyright DigitalGlobe catalog ID 1030010020C0C900, used under license by BAS). Figure 1 and Supplementary Figure 2 show updated positions for lakes, meltwater streams and other key geomorphological features. Lake bathymetry was determined from GPS-fixed, metre-scale measurements undertaken during coring. We then used the hydrological toolbox in ARC-GIS to determine catchment and lake area (in m^2) for all hydrological systems that have a prominent, *i.e.*, >5 m footprint and undertook bathymetric and landscape slope, sink and flow rate analysis.

Several studies have shown that catchment erosion and deposition in small closed-basins with a single catchment and non-complex inflow characteristics (*e.g.*, Ardley Lake), can be approximated by a linear relationship[54]; thus, a reasonable approximation of the total amount of sediment or, in this case, guano (G_D) delivered, by erosion G_e, into the Ardley Lake basin, can be obtained, simply from ratio of lake-area to catchment-area, expressed as an estimated percentage[54,55]. In addition to the scenarios described in the Methods section, we investigated several possible accumulation area scenarios from A_{max} downwards to constrain the most likely Ardley Lake basin accumulation area. The A_{max} scenario assumes that sediment accumulation area is the same size as the total area of Ardley Lake basin. However, the Ardley Lake basin is not uniformly flat, and it is unlikely that areas with steep slopes will accumulate sediment. Therefore, we compared the A_{max}

accumulation area with the area produced by the Z_A (%) factor in Blais and Kalff[56] as follows:

$$(1)\ Z_A\,(\%) = [49.92\,(\pm 3.37) - 2.50(\pm 0.31)] \cdot \mathrm{Slope_{mean}}$$
$$= [49.92\,(\pm 3.37) - 2.50(\pm 0.31)] \cdot 16.57\,(\pm 0.12)$$
$$= 8.5 \pm 6.2\%\ \mathrm{of}\ A_{max} = 618\,m^2 = 6.18\,x\,10^6\,cm^2$$

where $\mathrm{Slope_{mean}}$ was calculated using the ARC-GIS hydrological toolbox using bathymetric data measured during coring (Supplementary Fig. 2b). However, the Blais and Kalff[56] model assumes that most basin slopes are heavily skewed to lower slope values, which is not the case for Ardley Lake where slope angles are mostly in the 10–20 degree range, with a wide flat accumulation area covering most of the western half of the lake. The Ardley Basin has steeper edges close to the deepest plateau area (<3 m) in the eastern half of the lake (Supplementary Fig. 2c). The deepest part of the basin >3 m, at the eastern end of the lake, has an accumulation area, $A_{\min(>3m)} = 1{,}722 \pm 172\ (10\%\ \mathrm{error})$, which we consider to represent the minimum accumulation area within the basin (not shown in Supplementary Fig. 19). Since there will be sediment accumulation across the whole basin, we consider that the median slope value is c. 27 degrees and the lake area <30 degrees, $A_{<30} = 5{,}682 \pm 568\ (\pm 10\%\ \mathrm{error\ applied})$ provide the most realistic constraint on the stable sediment accumulation area within Ardley Lake (Supplementary Fig. 2). This investigated the following three possible accumulation area scenarios, but retain $A_{<30}$ from this point forward:

$$A_{\max} = 7{,}274 \pm 727\,m^2 = 7.27\,x\,10^7 cm^2$$
$$A_{<30} = 5{,}682 \pm 568\,m^2 = 5.68\,x\,10^7 cm^2$$
$$A_{\min(>3\,\mathrm{m})} = 1{,}722 \pm 172\,m^2 = 1.72\,x\,10^7 cm^2$$

Using $F_{o.sed.}$ DMAR measured data and catchment-delivery (yield) and hyperboloid and ellipsoidal basin accumulation equations that account for sediment focussing[54–57], we estimated the minimum, maximum and most likely Ardley Lake basin-wide accumulation of guano and colony population levels through time for these accumulation area scenarios as described in the Methods section (see Fig. 5 and Supplementary Fig. 19 for summary results).

Supplementary Note 9

Tephra deposits in the ARD and YAN records

Geochemically distinguishing between basaltic-andesitic tephra produced by individual eruptions from Deception Island using established shard specific major element electron probe micro-analysis techniques[58,59] is challenging. Since trace element characterisation and other more sophisticated discrimination methods (*e.g.*, Pb-isotope analysis, crystal-fraction geochemistry) remain at an early developmental stage for the Antarctic Peninsula region, we used a combination of micro-XRF (µ-XRF) scanning alongside traditional grain size and tephra layer thickness measurements to establish an eruption event chronostratigraphy (see also Supplementary Note 7, Supplementary Fig. 10, Supplementary Table 2). Individual eruption events, denoted by suffixes a, b, c etc., were assigned to a 1,000-year age event bracket by comparing the eruption size and weighted mean and 95% confidence interval age ranges data for each tephra layer in the ARD and YAN records with published ash deposit ages from other parts of the South Shetland Islands (*e.g.*, James Ross Island ice core and Beak Island lake records[28,34], see Supplementary Table 3) and summary published age data[60,61]. These papers provide a good composite assessment of several eruption ages from marine and terrestrial records

across the Antarctic Peninsula. Hereafter, for example, T1a, b, c represent major eruptions a–c between 0–1000 years. Numerous finer ash, and non-visible (cryptotephra) deposits have also been found in lake sediments across the South Shetland Islands[47,52]. These were generally less than 2 mm thick within our lake records and not included in our analysis because, in this study, we were interested in major eruptions that could have a distal impact on penguin colonies and finer layers are often reworked ash[47].

After the stratigraphic position of tephra deposits was visually identified, we used X-radiographs and statistical analysis of µ-XRF data to precisely determine the depth of ash deposition (Supplementary Figs. 3, 7, 9, 10). Wet sediment µ-XRF scanning was undertaken continuously down to 200 µm, providing an ultra-high resolution record of geochemical change in both the ARD and YAN sequences. Identifying tephra deposits using µ-XRF scan data worked best in the YAN record, where visible ash layers were characterised by elevated µ-XRF Ca, Ti, Mn, and Zr, and coeval reductions in K and Rb. In previous scanning studies, Zr has also been used as a proxy for grain size in mineral-rich sediment matrices while Rb has been associated with increased clay-fraction erosional input[62]. Ultra high-resolution datasets (200 µm) proved to be particularly useful for identifying tephra deposits in the top 45 cm of the YAN record that represents the last 5,000 years (Supplementary Fig. 11) and where traditional cm-scale subsampling lack sufficient resolution. In the ARD record, Ca values were an order of magnitude higher than in the YAN record because Ca associated with volcanic ash deposition was overprinted by Ca associated with hydroxylapatite ($Ca_5(PO_4)_3(OH)$) formed by guano deposition (Supplementary Fig. 6, 13, 16). In both records, visible ash layers were characterised by reduced Br/Ti, incoherent/coherent scatter (inc./coh.) ratios, since Br is commonly elevated in lake sediments with higher organic carbon content[62], and key scatter-normalised element percentages (*e.g.*, Ti, Ca, Fe, Mn). Key element/Ti ratios (*e.g.*, Ca/Ti, Mn/Ti and Br/Ti) compare favourably with Al- and Ti- normalised patterns in subsample XRF data (Supplementary Figs. 6, 13, 16; Supplementary Table 6). In the ARD record, total scatter normalised Ti-normalised Cu, Zn and Sr also correspond well with XRF-subsample $F_{o.sed.}$ *%* and PCA1 profiles. Thus, in some cases, future studies could utilise, µ-XRF scanning to rapidly identify (fine-scale) changes in lake sediments associated with guano input.

Although volcanic ash layers were readily identifiable in organic-rich sediments of the YAN record, it proved challenging to distinguish tephra deposits from the background sediment geochemistry because basaltic volcanic ash layers erupted from Deception Island have a similar bulk geochemical composition to the volcanic basement bedrock of King George Island. To overcome this issue, we also examined changes in fine-medium sand component, which is greater in airfall ash deposits than the mean background grain size deposition in both cores. After the influence of glaciogenically-derived sediment had receded at both sites by c. 8 ka, particle size and geochemical data suggest that the main supply of sand size sediment was from volcanic ash. In both cores, >0.5 cm thick black, sand-rich tephra layers were readily visually identified and prominent in fine-medium sand profiles (Supplementary Fig. 12).

In the YAN record µ-XRF wet sediment (untreated) analysis, the first PCA axis PCA1 explains >80% of the variance in the data and downcore patterns broadly reflect organic-minerogenic variations. Calcium has a strong component along the PCA2 axis in an opposite direction to Br indicating a dominant bedrock/tephra rather than organic source (Supplementary Fig. 11). PCA2 also defines a secondary trend in organic-minerogenic compositional change not associated with the main minerogenic input into the lake. Since Al counts are insufficient in ITRAX™ Mo-tube µ-XRF analysis, we use Ti as a normalising element. Titanium is the largest readily measurable crustally-derived component present in

μ-XRF analysis along PCA1 after Fe (which has multiple sources) and is commonly associated with catchment bedrock erosion (rather than, for example, aeolian sources, in both ARD and YAN[62]) (Supplementary Figs. 11, 13, 16). The similarity between downcore trends in scatter-normalised Br percentage data and Br/Ti in both profiles to broad scale changes in organic carbon (%TOC) and organic carbon-related proxies (*e.g.*, inc./coh. scatter ratio) suggests that Br is predominately sequestered by organic material in the ARD and YAN records (Supplementary Figs. 7, 10, 11). However, ARD and YAN are nearshore lakes in windy environments, and brackish diatoms comprise up to 90% of the diatom population in some parts of both cores. The relationship between marine organic matter (MOM) and Br is still debated[63]. Several studies have observed fixation and enrichment of organobromines in diatom frustules[64,65]. This or sea-salt derived Br[62] could account for Br-deviations away from organic trends in Geochemical Zone 6 of both ARD and YAN records.

We also compared Ca/Ti ratios from μ-XRF and XRF analysis of both records to determine whether Ca/Ti values in the ARD record were higher than the YAN record. Ca/Ti ratios are strongly correlated (n=276; R^2>0.79; RMSE=0.09; p<0.0001) in both records (Supplementary Fig. 13), but more well-constrained, and generally lower in the YAN terrestrial record (n=139; R^2=0.84; RMSE=0.0.049; p<0.0001; Supplementary Fig. 14). We identified some significantly elevated Ca and Ca/Ti values in the ARD record in μ-XRF and XRF data that indicate additional sources of non-bedrock derived Ca, most likely bone, sea spray and guano-influenced hydroxylapatite ($Ca_5(PO_4)_3(OH)$). The strong similarity between $F_{.o.sed}$ percentage and Ca/Ti ratio downcore profiles show that hydroxylapatite ($Ca_5(PO_4)_3(OH)$) formation is a dominant influence in the ARD record (Supplementary Figs. 6, 13, 16), but elevated Ca/Ti values in non-guano influenced and high PCA1 values in the brackish D2 diatom zone suggest increased sea-spray inputs and windier than-present day conditions could be responsible for additional Ca between c. 5.3–4.6 cal ka BP and 4.4–3.8 cal ka BP (Supplementary Figs. 16, 14). Unlike the YAN record, Ca/Ti ratios in the ARD record do not define the position of tephra deposits, but ARD μ-XRF and XRF downcore Ca/Ti and PCA1 patterns are coherent (84% and 88% of variance explained for μ-XRF and XRF, respectively).

Supplementary Discussion

In this study, we have examined several key environmental factors that drive centennial-millennial scale penguin colony change, namely: temperature, sea–ice distribution, precipitation and wind strength, along with increased land availability associated with a drop in relative sea level, and large volcanic eruptions from the nearby Deception Island volcano. Modern-day and past (palaeo)temperature and sea-ice records are known to vary spatially, on a regional basis, across the Peninsula[28,35,66]. Since some palaeotemperature and palaeosea-ice records were considered to be located too far from the SSI[26,28] or could reflect cross-Peninsula temperature differences[35] and local influences (*e.g.*, increased terrestrial glacial discharge), we developed our own local, South Shetland Island (SSI)-based, (atmospheric) palaeotemperature record from nearby Yanou Lake by applying the recently produced (sub)Antarctic region GDGT calibration curve[22] and examined reconstructed sea-ice changes from a core located at the present-day summer sea-ice limit in the Anvers Trough.

The Anvers Trough core was the nearest available record with consistently open-water spring-summer at the modern shelf edge. It is relevant to the SSI-WAP region because it is located between the mean modern-day (1981–2010 CE) Austral summer minimum (February) and winter maximum (September) sea-ice limits in the Bransfield Strait area

(Fig. 1a). The balance between open-water and sea-ice coverage at this location was considered less likely to be influenced by glacial meltwater and other terrestrial influences and more likely to reflect changes in the availability of sea-ice edge feeding grounds for penguins. We included a published sea-ice record from Maxwell Bay[27] to provide the most locally-available constraint on sea-ice conditions, and a constraint for the SSI side of the Bransfield Strait. Since the as-measured %TOC-based Maxwell Bay sea-ice dataset was noisy and not sampled at regular time intervals, we first undertook negative exponential regression smoothing and then resampled the data at 100-year intervals. This also allowed direct comparison with the JRI and Palmer deep records that were resampled at 100-year intervals. The Anvers Trough sea-ice records were significantly less noisy at 100-year intervals than the Maxwell Bay sea-ice record, and not significantly different to the as-measured diatom profiles shown in Figure 4.

Variations in climate and open water conditions on and around the South Shetland Islands and across the northern AP influence positive colony responses, but neither are consistent in time or space (Fig. 4, Supplementary Figs. 18, 19, Supplementary Tables 7, 8). Overall, there were no significant differences, at 5% or 10% significance level, between guano and non-guano phases, in cross-northern Peninsula 9–0 ka terrestrial mean summer air temperature (MSAT) data from Yanou Lake (YAN-GDGT <10 °C dataset) (this study) and (ice core-atmospheric) temperature anomaly data James Ross Island (JRI)[28], but non-guano phases were significantly warmer in the 9–0 ka Palmer Deep sea surface temperature (PD-SST 0–200 m) dataset[26] (Supplementary Table 8; two-tailed Mann-Whitney U-tests: YAN-GDGT <10 °C dataset: p=0.58, JRI: p=0.57, PD-SST: p=0.01; guano phases GP-1–GP-5: YAN-GDGT-MSAT <10 °C dataset weighted mean temperature ± 1σ = 2.74 ± 2.98 °C, JRI mean temperature anomaly ± 1σ = 0.102 ± 0.022 °C, PD-SST mean temperature ± 1σ = 0.48 ± 0.56 °C; non-guano phases: YAN-GDGT 2.19 ± 2.51 °C; JRI 0.021 ± 0.004 °C, PD-SST: 0.97 ± 0.76 °C).

Similarly, there were no significant differences in open water conditions in Maxwell Bay (p=0.26) or at the Anvers Shelf (p=0.25) during guano and non-guano phases, but more open water existed at the Anvers Shelf during mid Holocene guano phases than late Holocene guano phases (p=0.10), whereas significantly more open water existed in Maxwell Bay during late Holocene guano phases than in non-guano phases (p=0.03). For the YAN-GDGT temperature record, late Holocene guano phases (GP-4, GP-5) were significantly warmer (p=0.0003; 4.73 ± 2.40 °C) than mid Holocene guano phases (GP-1 to GP-3: 0.09 ± 0.59 °C; Supplementary Table 8). For the JRI temperature anomaly record, mid- and late Holocene guano and non-guano phases were statistically similar (mid: 0.157 ± 0.03 °C, late: 0.075 ± 0.016 °C; p=0.48), but late Holocene guano phases were significantly warmer (0.07 ± 0.016 °C; p=0.026) than non-guano phases (-0.224 ± 0.425 °C).

In the absence of significant changes in post T7-eruption local (Maxwell Bay) sea-ice conditions (Fig. 4c), the comparatively small initial colonisation of the Ardley Lake catchment during guano phase GP-1 (6.7–6.3 cal ka BP) was most likely driven by extended sea-ice seasonality at the shelf-edge during the spring/summer c. 6.7–6.0 cal ka BP (inferred from higher *Fragilariopsis curta / F. kerguelensis* diatom ratios and lower percentage of pelagic open water diatoms in the Anvers Trough marine sediment record), which was concordant with 'cooler' conditions in the Palmer Deep basin records from c. 7 ka onwards (Fig. 4d-e), and 'cooler' and increased 'sea-ice' conditions around the South Shetland Islands 7.3–5.2 cal ka BP. The start of a 'Mid Holocene Hypsithermal' at c. 4.5 ka in several terrestrial cross-Peninsula records[67,68] corresponds with the start of GP-3, and a more persistent phase of spring/summer open water conditions at the shelf-

edge. Increasing El Niño-South Oscillation (ENSO) frequency and Southern Hemisphere Westerlies (SWW) wind-strength[26] affecting the South Shetland Islands at this time is apparent as elevated brackish conditions in Ardley Lake from increased sea-spray input (Supplementary Fig. 17). Increased storminess and snow accumulation on Ardley Island and Fildes Peninsula likely reduced the length of the terrestrial snow/ice-free spring-summer season, making conditions less favourable for chick-rearing, while the extended lake-ice season and increased meltwater input from a late spring or summer melt into Yanou Lake could have supressed the water temperature in Yanou Lake, possibly causing it to decouple from mean summer air temperatures, until at least c. 3.8 cal ka BP (Supplementary Fig. 17). An absence of sub-fossils elsewhere on the AP during GP-3 and the generally cooler-than-6–0 ka average conditions in the Palmer Deep area that persisted during throughout the mid Holocene[26] might have made the mid-southern AP a less favourable location for penguin colonies (Fig. 5). The sustained period of spring/summer open-water at the Anvers trough shelf edge could have also led to reduced competition from ice-dependent species (*e.g.*, Adélie penguins), enabling the Ardley Lake gentoo colony to expand. Most notably, the late Holocene guano phase GP-4 $F_{o.sed.}$ DMAR maxima ($F_{o.sed.}$ % = 43.66 ± 15.37 (1σ) %) occurred when the YAN-GDGT and JRI records were in-phase and warmer than their 6–0 ka respective means (Fig. 4, see Supplementary Fig. 18, Supplementary Tables 7, 8), which coincided with a period of persistently reduced open water in the Anvers Trough record from c. 4 ka. Therefore, we conclude that a combination of 'more favourable' climate (warmer) and open water (reduced) at the shelf edge and in Maxwell Bay best explain some positive colony responses and guano phase development.

However, our main finding is that volcanic eruptions had the most significant negative impact on the Ardley Lake penguin colony. Comparison to shard-specific tephra data from the Scotia Sea and Boyd Strait in Moreton and Smellie[59] shows Deception Island is the source for all new data collected. Eruptions T7 and T9 have a bi-modal basaltic-andesitic and trachydacitic-rhyolitic composition. Evidence for a large early Holocene, c. 10.5–9.5 cal ka BP (T9), 'megascopic' (caldera-forming?) explosive eruption, with a bi-modal basaltic-andesitic and trachydacitic-rhyolitic composition, has been found in the Beak Island Beak 1 lake record and in the JRI ice core record[28,34,35] and several marine cores from the northern AP and Scotia Sea[58–60,69–71] (Supplementary Tables 3, 8, Supplementary Fig. 20, 21, Supplementary Data 7). Although basal unit radiocarbon ages from bulk glaciogenic sediments in the ARD record are the least reliable in the ARD record and the T8 eruption is not present in the YAN record which dates to c. 12 cal ka BP, tephra deposits equivalent in timing to ARD-T8 eruption have been found in lake sediments from Livingston Island at c. 8.2 ka[47]. There is no evidence of a major T8 event in the JRI and all other Peninsula records.

The T7 eruption event was probably the most explosive and widespread Deception Island eruption of the mid Holocene (Supplementary Figs. 16, 21). The T7 tephra has a similar bi-modal composition to the T9 tephra and has been found in several lake and marine cores from the NW-AP[47,51], but not in the Beak Island or JRI record[28,34]. This implies that: 1) several explosive eruption occurred between c. 7.5–7.0 cal ka BP; 2) that the ash cloud trajectory did not pass over (or deposit ash on) the NE Antarctic Peninsula; 3) that extensive reworking of the T9 (and/or T8) eruption events occurred across the Peninsula during early Holocene deglaciation, unlocking tephra stored in glacial ice. It is difficult to precisely disentangle individual eruptions in this part of the record due to large radiocarbon age uncertainties and reworking. Nevertheless, the ages of the key eruption events T3a/b, T5 and T7 tephra in ARD and YAN records are consistent with ages of 3.31/3.72 ± 0.07, 5.00 ± 0.07 and 6.92 ± 0.07 ka for the largest airfall tephra deposits found in marine cores

from the Bransfield Strait[51]. As these marine-based ages were obtained using relative palaeomagnetic intensity correlation dating, they provide an independent cross-check on radiocarbon-based eruption ages in this and previous studies.

The causes of penguin mortality related to volcanic activity probably mirror those of humans, though specific percentages will vary significantly. Close to the eruption source (<20–50 km), approximately 33% of all human deaths associated with volcanoes have been caused by direct contact with pyroclastic flows, while indirect physical processes, which also affect distal locations, causing a further 24% of known fatalities[72,73]. Circumstantial evidence from the volcanically-active South Sandwich Islands shows that, close to an erupting volcano, penguin colonies prefer not to nest on recent ash fall, which could also cause high, but localised, mortality rates (Supplementary Fig. 23). At more distal locations, indirect processes, such as burial by tephra, the generation of tsunamis and (supraglacial) lahars, and the deposition of fine ash layers and aerosols, pose a substantial physiological risk to penguins[74,75].

The most disruptive eruption events occurred during the mid-Holocene c. 5.4–4.8 [5.5–4.6] cal ka BP (T5 in Fig. 4, Table 2; Supplementary Fig. 10, Supplementary Table 3). Silt/sand sediment flux profiles for the ARD and YAN records (Supplementary Fig. 16) both show increased reworking of coarser catchment material into the lake c. 6,000–3,000 years ago, supporting our hypothesis that absence of terrestrial sediment sequences older than c. 2,500 years on Ardley Island could be because substantial landscape erosion occurred during the mid-Holocene. We also found thick ash layers composed of coarse sand-size deposits at this time in other lake cores we took from Fildes Peninsula[15], for example: 1) an ash layer >1 m thick in Belen Lake with an upper age constraint of 4,829 ± 38 ^{14}C a BP (weighted mean age = 5,510 ± 60 cal a BP and min-max 2-σ age range: 5,600–5,330 cal a BP) which is equivalent in age to T5a in the YAN and ARD records; 2) large airfall ash deposits >5 cm thick in marine-sediment sections of the Long Lake record immediately after 6,445 ± 40 ^{14}C a BP (6,150 ± 80 and 6,290–5,990 cal a BP), equivalent to T6, and at 5,872 ± 40 ^{14}C a BP (5,530 ± 70, 5,330–5,660 cal a BP = T5) and approximately two metres of reworked ash layer deposited in terrestrial sediments deposited immediately after this eruption event, which is equivalent to the thick unit of ash deposited in the YAN record between c. 40–190 cm depth. Other ash layers found in all records are smaller and not followed by such extensive reworking. Substantial airfall deposits or melt-season reworking of T5 event tephra are also apparent in other N-AP lake, marine and ice core records[28,34,47,51,52,61] (Supplementary Table 3).

As well as the potential hazard posed by catchment destabilisation and lahar development following large eruptions, most Deception Island eruptions commonly produce fine basaltic-andesitic tephra containing volcanic glass, which is highly abrasive to soft tissues, and a highly toxic aerosol (SO_2, F) and trace metal (*e.g.*, Cd, As, Pb) content[58,59] (*e.g.*, fluorine (F) in magma: 112-461 ppm[76]; F in volcanic glass shards: 522 ± 128–1,123 ± 224 ppm this study (≡ mg kg^{-1} in Supplementary Table 10); SO_2 in glass: 22–2,764 ppm, this study). Nearly 80% of the livestock in Southern Iceland were poisoned by the addition of 250 ppm F from the effusive basaltic Laki 1783 eruption, leading to the deaths of nearly one third of the Icelandic population in a subsequent famine[74,77,78]. Most terrestrial animals (except penguins) have F bone concentrations of 500–1,000 ppm, and in humans, F-induced osteosclerosis occurs when concentrations reach 3,500–5,500 ppm[79]. Heavy metals, such as Cu, Hg and Cd are often bio-accumulated in top predators, such as mammals and seabirds in the Southern Hemisphere due to their high Cu, Cd and Hg dietary intake[19,80,81]. Thus, penguin or sea mammal droppings can be a source for these elements. In contrast, the behaviour of Hg in sediments is more complex. In its methylated

form, mercury is highly mobile and gradually released from the sediments after deposition[82]. However, since Se and Hg show similar trends in ARD sediments (Fig. 2) the formation of more stable HgSe (tiemannite) and CdSe, which is an end product of biochemical detoxification, is possible[83–87]. Fluorine is also naturally exceptionally high in krill, whales, fish and seabirds from the Southern Ocean and Antarctica[88] (*e.g.*, F in Krill: muscle = 50 ppm, exoskeleton = c. 3,000–5,500 ppm[79,84,89]; F in Adélie penguin bones: up to c. 7,000–10,000 ppm[79,90]; F in guano = 4,300 ppm[84]). Coping mechanisms include the storage of nearly all additional F in krill-chitin by penguins in their bones[19,79,90]. The respiratory effect of a combination of fine, abrasive volcanic glass containing a highly-toxic combination of additional trace and heavy metals is unknown, but prolonged exposure would more-than-likely adversely affect the otherwise well-adapted physiological processes (*i.e.*, digestion, respiration, immune function) of penguins and their entire predator-prey food-chain.

Supplementary Methods

Lake sediment sedimentology

Field measurements, the main changes in lithology (defined by μ-XRF geochemistry, gamma-density, loss-on-ignition (LOI), Magnetic Susceptibility (MS), grain size and geochemical data), the position of visible moss deposits, and other marker horizons, were used to stack core sections (Supplementary Figs. 3, 9) into composite sequences. Precise alignment of core overlaps was undertaken using μ-XRF and GEOTEK scanning data. Whole core gamma (bulk) density (2 mm aperture gamma ray attenuation sensor), magnetic susceptibility (MS) (Bartington Instruments MS2G 1 cm loop sensor, 10 s) and P-wave velocity were measured with a GEOTEK core logger using standard calibration procedures[91]. To measure fine-scale changes in bulk *as-deposited* (*i.e.*, wet) sediment geochemistry, contiguous μ-XRF geochemical core scanning was undertaken on flat half-core wet sediment surfaces using an ITRAX™ core scanner at Aberystwyth University fitted with a Molybdenum (Mo) X-ray tube. X-radiography settings were 50 kV, 50 mA, 200 ms; μ-XRF_{Mo} settings: 30 kV, 50 mA, count time 10 seconds, 200 μm or 2 mm interval with 50- or 5-point moving average for 1 cm subsample interval equivalence. Machine and sample calibration was undertaken using a synthetic glass standard and XRF fused glass discs from the YAN and ARD core at the start and end of each core-site run. Raw count per second (cps) data were analysed using the Q-spec spectra matching software (Cox Analytical). As we were interested in primarily patterns of downcore variability, data are presented as total scatter-normalisation ratios and percentages of the total scatter-normalised sum, following established total-scatter normalisation procedures[62,92], rather than equivalent whole-rock percentages. This avoids creating spurious values for elements that cannot be measured (*e.g.*, Na, Mg) or have low count values (*e.g.*, Al, Si) when using an ITRAX™ Mo-tube[62]. Since large variations in grain-size can affect μ-XRF scans and other datasets[93], very coarse-fractions (>8 mm and >2 mm) were removed, weighed and classified[46]. Particle size analysis was undertaken on the <2 mm (sand–silt–clay) fraction at 2–4 cm intervals using a Coulter Particle Size Analyser LS230 (range 0.4 μm–2 mm) following pre-treatment with dilute HCl and H_2O_2 to remove carbonates and organics. Grain-size parameters were modelled using the Fraunhofer model and Udden–Wentworth geometric grain size divisions and interpolation of data was undertaken using v.3.01 of the Coulter LS Control software with statistical analysis undertaken in GRADISTAT v5[94]. Contiguous 1 cm interval subsamples were also used to determine dry mass (g), organic and carbonate content based on % weight loss-on-ignition after combustion at 550 °C for 2 h (to estimate changes in organic carbon) and 950 °C for 4 h (to estimate changes in carbonate)[95]. Dry mass accumulation rates were calculated using standard procedures

(*i.e.*, by multiplying the dry bulk density (g cm^{-3}), the sedimentation rate (cm a^{-1}) and the percentage concentration[96,97]).

Lake sediment chronology

We used a combination of radiocarbon, Pb-210 and Cs-137 dating to construct chronological profiles for the ARD and YAN lake cores. Aquatic mosses dominate the sedimentation style of the upper 2 cm of the YAN core, and the very slow sedimentation rate in both cores during the late Holocene meant Pb-210 and Cs-137 dating were unsuccessful, with data obtained from only the top 1 cm of the ARD core. Hence, all cores are based on radiocarbon dating of, in order of preference, aquatic and terrestrial mosses (identifiable to species level), other discrete macrofossils and identifiable fossils, organic-rich sediments and bulk inorganic sediments[48].

Macrofossils were hand-picked from frozen bulk material after overnight defrosting at 5 °C; then immersed in ultra-pure (18.2 MΩ) water, sealed and placed in an ultrasonic bath for an hour, sieved to remove fine particles, and picked under a binocular microscope and dried/refrozen and sent to either the NERC Radiocarbon Facility (East Kilbride), the Oxford Radiocarbon Accelerator Unit or Beta Analytic (Miami, Florida) for accelerator mass spectrometry (AMS) radiocarbon dating. Samples submitted to the NERC Radiocarbon Facility (East Kilbride) were prepared to graphite by digesting the sample in 0.5 M HCl (room temperature for 16 hours) for moss macrofossil samples or 2 M HCl (80 ºC for 8 hours) for all other samples, rinsed in deionised water, until all traces of acid had been removed, dried in a vacuum oven and homogenised. The total carbon in a known weight of all pre-treated samples was recovered as CO_2 by heating with CuO in a sealed quartz tube. The CO_2 was converted to graphite by Fe/Zn reduction. Graphite targets were passed to the Scottish Universities Environmental Research Centre AMS Laboratory (East Kilbride) for ^{14}C measurement (5MV NEC AMS) and ^{14}C results were corrected to $\delta^{13}C_{VPDB}$‰ -25 using ^{13}C values measured on a duel inlet stable isotope mass spectrometer (VG Optima) which were representative of the original pre-treated material and which are shown in Supplementary Table 2.

Samples dated by Beta Analytic were leached with a 0.5 M to 1.0 M HCl bath to remove carbonates and heated to 70 °C for 4 hours. Leaching was repeated until no carbonate remained, followed by rinsing to neutral 20 times with deionised water, then placed in 0.5% to 2% solution of NaOH for 4 hours at 70 °C and rinsed to neutral 20 times with deionised water. The process was repeated until no additional reaction (typically indicated by a colour change in the NaOH liquid) was observed. Samples were then leached again in a 0.5 M to 1.0 M HCl bath to remove any CO_2 absorbed from the atmosphere by the NaOH soakings and to ensure initial carbonate removal was complete, and then dried at 70 °C in a gravity oven for 8–12 hours.

To determine if combined radiocarbon dating and DNA analysis was achievable, we extracted and dated collagen from three (of 11) bones at 124–126 cm, 143–147 cm and 289–292 cm depth. Since the extraction and dating process is destructive, thus far, we have dated bones only from parts of the core where duplicate bones samples exist. We dated the collagen fraction because it is least likely to be contaminated by post-depositional bacterial activity and thought to produce more 'reliable' ages. The possibility of extracting fossil DNA increases if bone-collagen can be extracted, and we are currently investigating further DNA analysis of penguin bones and sediments. Bone-collagen was extracted and dated by Beta Analytic using standard bone treatment procedures described on their website (www.radiocarbon.com). Analytical procedures for Anvers Shelf core samples sent to the Oxford Radiocarbon Accelerator Unit followed established methods[98].

Radiocarbon age data are reported as conventional radiocarbon years BP (^{14}C a BP) ± 1σ, and as two-sigma (2σ, 95.4%) calibrated age ranges, mean ± 1σ, and median calibrated ages (cal BP relative to 1950 CE) (Supplementary Table 2). Calibrated age data in Supplementary Table 2 were rounded to the nearest 5 years where measured radiocarbon age errors were less than ± 50 radiocarbon years and to the nearest 10 years where measured radiocarbon age errors were greater than ± 50 radiocarbon years. For freshwater samples, calibration of terrestrial ^{14}C ages shown in black in Supplementary Table 2 was carried in OXCAL v. 4.2[99] using the SHCal13 Southern Hemisphere atmosphere dataset[100,101]. Absolute percentage of modern carbon (pMC) data were corrected according to ^{13}C/^{12}C isotopic ratios from measured pMC, where a "modern" pMC value is defined as 100% (1950 CE). Post-bomb (>1950 CE) samples were corrected according to ^{13}C/^{12}C isotopic ratios from measured pMC, where the 'present day' pMC value is defined as 107.5% (2010 CE), and calibrated using the SHCal13 SH Zone 1-2 Bomb curve using CALIBomb[102,103].

Age-depth modelling was undertaken using BACON v.2.2 (Bayesian) age-depth modelling software by inputting uncalibrated conventional radiocarbon age data in R (^{14}C a BP ± 1σ error, Supplementary Table 2)[104,105]. BACON is an age-depth modelling program run in R, which requires the as-measured, radiocarbon age and its one-sigma measured error as input data[104]. It reconstructs Bayesian accumulation histories in 5 cm (or smaller) segments progressively upcore by calibrating radiocarbon ages and combining this with other 'prior' information. The priors, such as the starting and overall mean accumulation rate are defined so that, in essence, sediments cannot be deposited backwards in time. Outlying calibrated ages are automatically ignored as the program runs several million Markov Chain Monte Carlo (MCMC) age-depth modelling iterations, which estimate the accumulation rate in each 5 cm segment and how it relates to the 5 cm section above, after the as-measured radiocarbon age data inputted has been calibrated. Varying the prior settings apart from the acc.mean from default settings has no significant effect on age-depth profiles produced, mainly because the default BACON settings encompass a wide range of most likely encountered scenarios. All ages and years are rounded to the nearest 10 years. Interpolated modelled ages quoted in the text were derived from the 'best-fit' age of the BACON age depth model (weighted mean age), with minimum to maximum 95% confidence ages always shown in square brackets. While it was necessary to calculate ages for some proxy data (*e.g.*, sub-cm resolution scanning data) to enable plot proxy data against time, ages in the main text interpolated from the age-depth model were rounded to the nearest 10 years and, where appropriate, interpolated 95% confidence interval calibrated age ranges shown in brackets (also rounded to the nearest 10 years). Smoothed 100-year interval datasets better reflect the level of precision for all dating methods used in this study. Terrestrial samples were calibrated using the SHCal13 (SHZ1-2) calibration curve during the modelling process, producing the same results as shown in Supplementary Table 2. The MARINE13 calibration curve[102,106,107] was used to calibrate marine or marine influenced sediments (including guano-phase bulk sediments), bone-collagen samples by applying the standard Antarctic Peninsula penguin-bone 1,100 ± 50 year total marine reservoir effect (MRE) (ΔR = 700 ± 50 plus the global MRE of 400 years). Prior settings for the ARD age-depth models runs were: acc.shape = 1.5, acc.mean = 50 a cm^{-1}, mem.strength = 20, mem.mean = 0.4, segment thickness = 5 cm. We investigated the effect of varying the starting mean accumulation rate setting in the first 5 cm (basal) segment using three accumulation mean settings acc.mean = 10, 20 and 50 a cm^{-1}. The choice of mean accumulation rate makes minimal difference in models ARD M1–M4, but starting accumulation rate of 50 a cm^{-1} produces the best fit for the sample age data in the ARD-M5 model due to the large age difference between the two

near-basal ages and the additional 700-year local MRE applied offset to the second to last bulk sediment age sample (age 17) within guano-phase GP-1 (see below and Supplementary Note 7 for results).

The setup conditions for ARD-M1, M4 and M5 age-depth model runs shown in Supplementary Figure 5 were as follows: *(a) ARD-M1: Fixed SH13.* Original age-depth model, first developed in 2013, using lake sediment data only with no reservoir effects included. This is an entirely terrestrial sediment/lacustrine macrofossil dataset with no bone-collagen ages included; *(b) ARD-M4: Fixed marine reservoir value of 700 ± 50 applied to bone-collagen only.* Lake (SH13) and penguin bone data (MARINE13) with $\Delta R = 700 \pm 50$ for all penguin bone data. This model assumes that the penguin bone-collagen ages and the $\Delta R = 700 \pm 50$ are correct and that bulk sediment ages have a purely terrestrial source. Results show that bone-collagen ages in Guano Phase 4 (ages 9/10) are all significantly younger than their bracketing lacustrine bulk sediment ages (ages 4, 8, 11, 13) by c. 700 years. This constant c. 700-year age-offset from the ARD-M4 weighted mean age-depth model seems to rule out the more variable ageing effect associated with 'old-carbon' contamination. Moreover, the strong correlation between carbon and $F_{o.sed.}$ implies the assumption that carbon in all bulk sediments has a purely terrestrial source is incorrect. For these reasons, the ARD-M4 model was not considered accurate; *(c) ARD-M5: Fixed marine reservoir value of 700 ± 50 applied to bone-collagen and bulk sediment ages from guano-influenced phases.* ARD-M5 model assumed that carbon in bone-collagen and bulk sediments in guano-influenced phases was derived from marine sources. We used this age-depth model in this study; *(d) ARD-M5: setup as (c), but with a MRE also applied to bulk sediment age no. 11 from a predominantly brackish section of the core.*

Bulk sediment geochemistry

For bulk sediment ICP-MS and G-AAS analyses acid digestions were performed on selected samples. 100 mg of each sample was given in a closed polytetrafluoroethylene (PTFE) vessel system[108] and pre-oxidised with 0.5 mL $HClO_4$ (70%, suprapur®, Merck, Germany) at 180 °C for 2 hours. After 3 mL HF (40%, suprapur®, Merck, Germany) have been added samples were heated at 180 °C for 6 hours. The acids were then evaporated at 180 °C on heating blocks while the residuals (200 µL) were re-dissolved with 3 mL 6-N HCl (subboiled) for three times. Subsequently 1 mL HNO_3 (65%, subboiled) and 10 mL ultra-pure (18.2 MΩ) water were added to the residuals and samples were simmered at 60 °C for one hour. Concluding the solutions were again heated at 180 °C in closed vessels for another 8 hours to achieve a total digestion and diluted to 25 mL afterwards. At least 7-fold measurements of several carefully selected in-house (*PS-S, UT-S, TW-TUC, Loess, BB-TUC, ICBM-B*) and international reference standards (AGV-1, GSS-6, GSD-2, BE-N, NIST-1515 Apple Leaves) were conducted in order to determine the precision and accuracy of the geochemistry measurements. To determine the degree of statistical spread and therefore the precision of each method the relative standard deviation (RSD) was used[109]. In case of TC, TS, TN, and XRF analyses multiple determinations of reference material were performed to calculate the pooled relative standard deviation (RSD_{pooled})[109]. Accuracy of the method was constrained by the relative error *f* representing the variation from the certified value[109]. Precision was better than 1.5% for major elements (Si, Ti, Al, Fe, Mn, Mg, Ca, K, P) and <5% for bulk parameters (TS, TN, TC, TIC) and minor elements (Ba, Co, Hg, Sr, V, Y, Zr, Zn, REE). Exceptions are given by As, Cu and Ni (RSD_{pooled} <8%). In general, accuracy ranges from -3.1 to 4.5% (major element oxides and bulk parameters) and -8.2 to 5.1% (minor elements), except for Na_2O (9.2%), MgO (7.7%), Cu (7.8%), and Gd (-10.8%). Where f >5% (major element oxides) and f > 10% (trace

elements) correction factors were calculated based on the reference samples in order to minimise systematic errors.

Anvers Trough record

During RRS Discovery cruise D172 (1987–1988) a gravity core (GC047, 380 cm) and a trigger core (TC046, 110 cm) were recovered in the Anvers Trough (64° 35.298 S, 64° 48.300 W) from a water depth of 536 m (Fig. 1b). Both marine cores TC046 and GC047 were spliced together using their magnetic susceptibility records. The spliced record comprises the top 0.4 m of TC046 together with the whole of GC047 resulting in a master core with a total composite sediment depth of 420 cm. All depths for GC047 are the original core depth plus 40 cm. Radiocarbon data from the Anvers Shelf record were calibrated using MARINE13 (100%) calibration curve, with a ∆R value of 1,470 ± 70 years, which represents the 1,870 ± 70 core-top surface age obtained for this core minus the global marine reservoir of 400 years. The BACON Bayesian age-depth model based on four AMS radiocarbon dates is straightforward and shows the composite core spans 11,785 to 106 cal a BP with a minor age reversal between 400 and 419 cmcd (minimum reversal 262 years, maximum 714 years). There are no major changes in sedimentology in this core; hence the smaller number of radiocarbon ages in its uppermost sediments shown here are appropriate, as is the use of a larger reservoir effect at this location because it is a deeper and more open water locality than Maxwell Bay.

Slides for diatom assemblage analyses were prepared following the method of Scherer[110]. Samples taken at 8 cm intervals throughout core TC36/GC47 were dried thoroughly in a warm oven at c. 30 °C; 5–20 mg of bulk sediment were sub-sampled into 30 mL vials; samples were cleaned and disaggregated using 3 mL of hydrogen peroxide (30%), 1 mL dilute hydrochloric acid (5%) and Calgon® solution for a minimum of 12 hours in a water bath at c. 50 °C; settled through a >10 cm water column onto cover slips for at least 4 hours; cover slips were allowed to dry before being mounted onto microscope slides using Norland Optical Adhesive (refractive index = 1.56). At least 300 diatom specimens were counted at x1,000 magnification, using an Olympus BH2 Light Microscope with x10 magnification eye pieces and an Olympus S Plan x100 oil immersion lens. Species identifications were based on descriptions by Tomas[111] and Scott & Marchant[112]. Relative abundances of the Antarctic sea-ice diatom *Fragilariopsis curta* and the open ocean diatom *Fragilariopsis kerguelensis* were calculated and used in a ratio to represent a gradient from sea-ice to open ocean environments to infer changes in the dominant oceanic conditions over time. The key Anvers Shelf core marine diatom species ecological preferences are as follows: *Fragilariopsis kerguelensis* (O'Meara) Hustedt is a pelagic, marine pennate diatom, most abundant in the open ocean zone south of the Polar Front. *F. kerguelensis* is considered endemic to Southern Ocean waters. Reports from the surface waters show *F. kerguelensis* dominates the assemblages of the open ocean zone south of the Polar Front. Highest abundances are observed in locations where summer open ocean conditions exist and little, if any, sea-ice occurs. *Thalassiosira lentiginosa* (Janisch) Fryxell is a pelagic, marine centric diatom, highest abundances occur from between the maximum winter sea-ice edge, across the Polar Front to the Sub Antarctic Front. Maximum occurrences of the *Thalassiothrix* spp. group have been reported in surface waters of the South Atlantic, both north and south of the Polar Front in Antarctic and sub-Antarctic waters. Very low occurrences are found in the seasonal sea–ice zone and the Antarctic coastal regions. The distribution of the *Thalassiothrix* spp. group against modern summer SST shows a maximum abundance between 1 and 3 °C and a general drop toward warmer temperatures. As the *Thalassiothrix* spp. group specimens are very long and narrow, valves are frequently broken into many parts. The *F. curta/F. kerguelensis* ratio relates to two sea-ice proximal species and is thought to reflect the

amount of UCDW on the continental shelf in the Anvers Trough core location. It represents the dominant control on diatom production associated with the open ocean (low values) versus productivity blooms associated with increased sea-ice cover at a particular site. The ratio has been used previously in several studies[113–116] and provides a useful amalgamation of three proxies for sea-ice extent (winter, spring, summer sea–ice proxies), which largely reflect the length of sea-ice cover season.

Tephrochronology - Electron Probe Micro-Analysis (EPMA)

EPMA of 165 glass-shards from the most prominent T4–T7 tephra layers in the YAN, ARD and Beak1 Lake records (Beak Island; Layers $T_{a–e}$[34]) was used to determine the eruption characteristics and source of mid-late Holocene eruptions that likely had the biggest environmental impact. Results were compared with glass-shard analyses from age-equivalent tephra layers in marine cores PC460/461 from the Scotia Sea (new data; this study) and similarly analysed data in Moreton and Smellie[59] from the Scotia Sea, northern Weddell Sea and Boyd Strait, and in Fretzdorff and Smellie[58] from the Bransfield Basin (Supplementary Figs. 1, 19, and Supplementary Table 7). Samples for EPMA were prepared by wet sieving through 250–63 µm, 125–63 µm and 63–10 µm disposable meshes, with organic and mineral residues removed by floatation in sodium polytungstate with densities of 2 or 2.45 g cm^{-3}. Oven-dried 250–63 µm and 125–63 µm volcanic glass-rich concentrates were mounted, polished and carbon-coated and analysed at the School of Geosciences, University of Edinburgh using a Cameca SX100 electron probe analyser, operating at 15 kV with a beam diameter of 5 µm, analysing of Na, Mg, Al, Si, K, and Ca at 2 nA and F, P, S, Cl, Ti, Mn, and Fe at 80 nA[117]. For all elements, the Kα X-ray line was measured and counting time on peak was 20 s and on background positions 10 s, except for F (50 s+40 s), Mn (60 s+50 s) and Fe (50 s+40 s). Spectrometer calibration used natural jadeite (Na), wollastonite (Si, Ca), apatite (P), barite (S), orthoclase (K) and synthetic glass (BIR-1G, Al), spinel (Mg), MnRb fluoride (F), halite (Cl), rutile (Ti), fayalite (Fe) and pure metallic Mn. Beam current was determined by the insertion of a Faraday cup into the path of the beam, and analyses made from a single point in the thickest part of each shard. Where possible, 10 and 30 shards were analysed per sample[118]. The number of analyses (n) represents the number of individual shards analysed, with one analysis per shard undertaken to avoid the effects of beam induced element mobilisation from adjacent locations. Na and K were measured during the first counting period to minimise the possibility of beam-induced mobilisation. Iron data is presented as total iron(II) oxide sum (FeO_{tot}). Counter dead time was corrected for and a ZAF correction applied to account for the effects of atomic number (Z), absorption (A) and fluorescence (F)[119] via Cameca's PeakSight software (version 4.2) using the X-Phi data processing algorithm. Calibration to volcanic glass standards (USGS fused basaltic standard BHVO-2G and the Lipari Obsidian) was performed at the start and end of each day. New sample data was normalised to 100% on a water-free basis ($H_2O_{diff.}$ = 100-%Total) to enable robust correlation to published data analysed using different setup[120]. Comparison to shard-specific tephra data from the Scotia Sea and Boyd Strait in Moreton and Smellie[59] (microprobe setup: 20 kV accelerating voltage, 15 nA beam current, 1 µm focussed beam, 10 second count time) and data from the Bransfield Strait[58] shows Deception Island is the source for all new data collected, and that eruptions T7 and T9 have a bi-modal basaltic-andesitic and tracydacitic-rhyolitice composition. Total percentages for all data shown in Supplementary Table 10 and in Supplementary Fig. 21 were >95%. Additional elements P_2O_2, SO_2, F and Cl were measured in the Beak Island and Moreton and Smellie[59] analysis. SO_2 was not included in Fretzdorff and Smellie[58] analysis. In total, these elements consistently constitute <0.5% of total percentages (Basic-Intermediate: 0.47 ± 0.12%; Trachydacitic-rhyolitic: 0.28 ± 0.19%) and, therefore, have minimal impact on correlation.

Analytical Software

Hierarchical R-mode cluster analyses were conducted with the R package 'Pvclust' version 1.2-2[121]. Downcore cluster-zonation was undertaken on ecologically-grouped diatom percentage data and square-root transformed and standardised (mean-1σ) geochemical data using the R packages Vegan[122,123] and Rioja[124,125]. Downcore trends in diatom and geochemical datasets using Principal Components Analysis (PCA) were summarised using C2 software[124] and square-root normalised standardised (mean-1σ) percentage XRF data and µ-XRF (Total Scatter Normalised (TSN)) percentage geochemical data and percentage diatom count data. Additional statistical analysis and plotting of data was undertaken in RStudio v. 0.98.939 using R v.3.0.1 and Community Ecology Packages Vegan v.2.0-10[123], Rioja v.0.8-5[126], C2 v.1.7.6[124], XLStat v.2010.3.09, SigmaPlot v.12.5, and AnalySeries v.2.0.4 software[127].

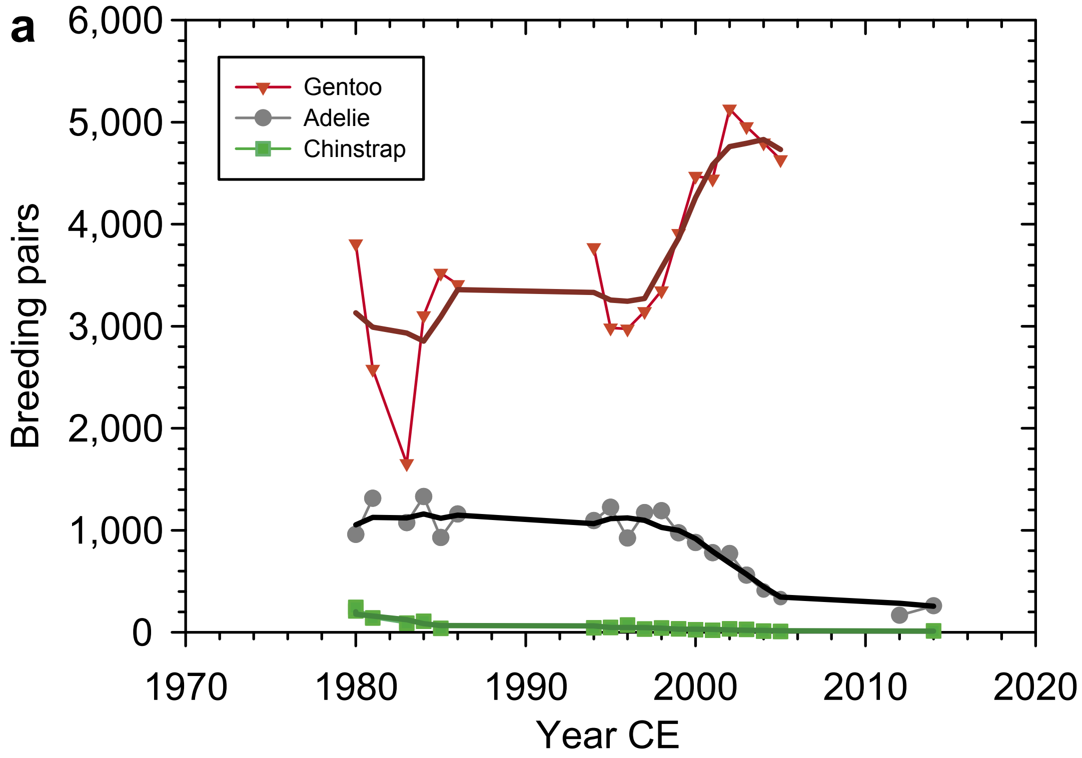

b

Mann-Kendall trend test (Two-tailed)

Species	Gentoo	Adélie	Chinstrap	ALL
Observations	18	20	19	**18**
Years CE	1980–2005	1980–2014	1980–2014	**1980–2005**
Minimum	1,656	166	9	**2,821**
Maximum	5,131	1,331	244	**5,937**
Mean	3,703	876	66	**4,724**
Std. deviation	917	357	66	**712**
Kendall's tau	0.556	-0.653	-0.778	**0.451**
S	85	-124	-133	**69**
p-value (Two-tailed)	0.001	< 0.0001	< 0.0001	**0.009**
alpha	0.05	0.05	0.05	**0.05**
No trend risk	<0.09%	<0.01%	<0.01%	**<0.85%**

c

MA Smoothing: Goodness of fit statistics

Statistic	Gentoo	Adélie	Chinstrap	ALL
Samples	18	20	19	**17**
Years	1980–2005	1980–2014	1980–2014	**1980–2005**
SSE	3,157,332	222,031	8,537	**3,618,162**
MSE	175,407	11,102	449	**212,833**
RMSE	419	105	21	**461**
MAPE	0	0	0.000	**0.000**
MPE	-3.354	-3.948	-12.084	**-1.793**
MAE	305	81	15	**323**
R^2	0.779	0.908	0.892	**0.577**

Supplementary Figure 1. Modern observational data for penguin population variations over the last c. 30 years on Ardley Island. (a) Summary plot showing gentoo, Adélie and chinstrap breeding pair data and results of 5-point running mean trend analysis (thick lines). **(b, c)** Statistical trend analysis and tabulated results for individual species and all species combined. Data downloaded from MAPPPD: Mapping Application for Penguin Populations and Projected Dynamics (www.penguinmap.com/mapppd).

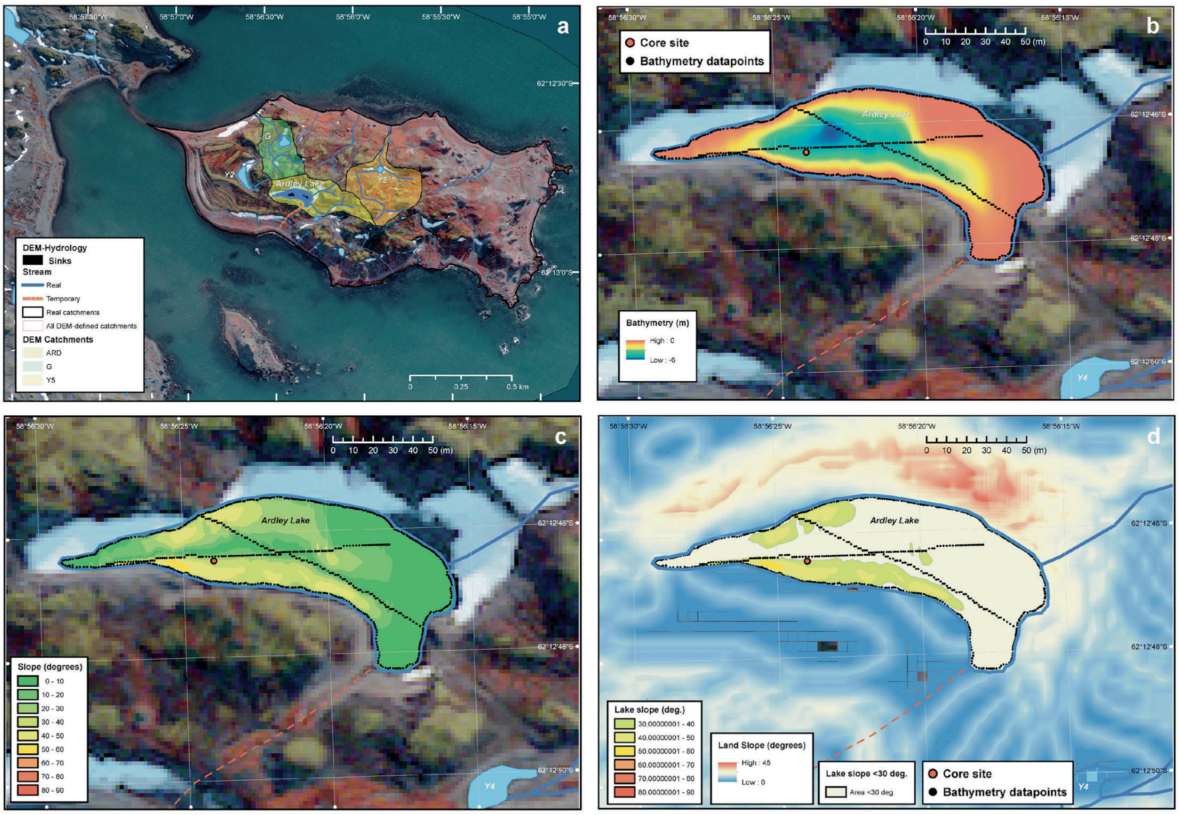

Supplementary Figure 2. Summary hydrological analysis results for Ardley Island. (a) Only three 'real' catchment areas exist on the island. Real is defined as catchments that have meltwater stream systems contained entirely within the ARC-GIS boundaries. All other catchment areas defined are shown in red. **(b)** Bathymetric data for Ardley Lake highlighting the deepest part of the basin >3 m depth in the central-eastern area of the lake. The main ARD core was taken from the most stable ice-covered platform at the southern boundary of the deepest part of the lake. The central deepest part of the basin was partially-moated and considered dangerous to work off. **(c)** Summary results of slope analysis for the Ardley Lake basin data. **(d)** Catchment slope and Ardley Lake basin accumulation areas with slope angle less than 30 degrees. This figure includes material © Digital-Globe, Inc., All Rights Reserved, used with permission under a NERC-BAS educational license and not included in the Creative Commons license for the article.

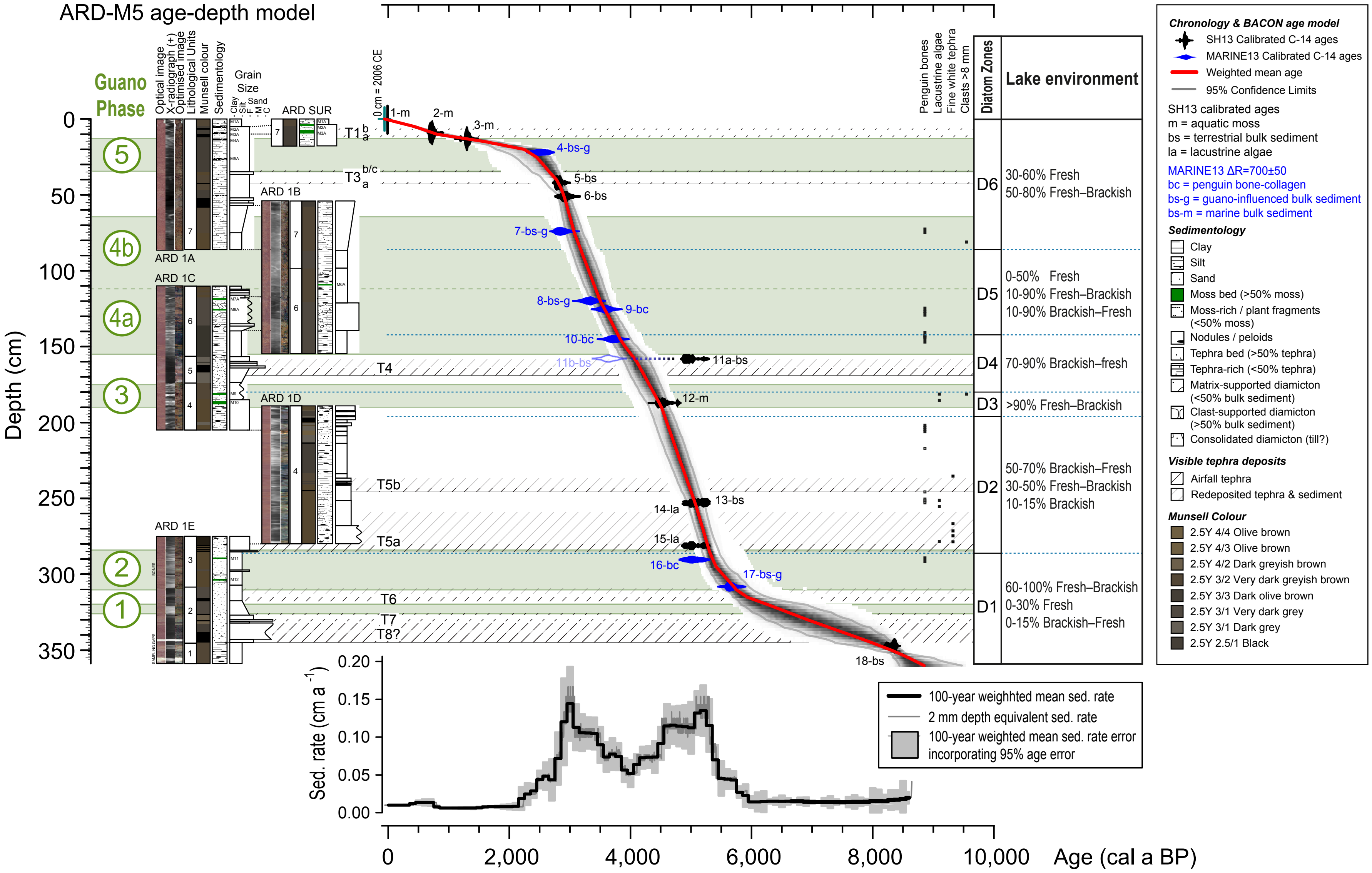

Supplementary Figure 3. Lithology & age-depth model for the Ardley Lake sediment core record. Lithology, age-depth model, summary ecology of diatom zones and sedimentation rates for the Ardley Lake sediment record. Visible tephra deposits T1-8 are shown by a diagonal line shading with parts of the core containing >50% reworked tephra deposits shaded in dark grey. Age depth modelling (ARD-M5) was undertaken in BACON v. 2.2 using SHCal13 calibration curves and prior settings: acc. shape 1.5; acc. rate mean 50 a cm^{-1}; mem. mean 0.4; mem. strength 20 producing 73 5-cm sections between 0–360 cm; airfall tephra deposits >5 cm thick were assumed to have been instantaneously deposited; all age data was included and the type of material dated is as shown in the legend and as described in Supplementary Table 2. The shaded grey area is a density plot of every 375th accepted iterations of 16.5 million iterative curve fits; the dotted black line is the 95% confidence interval limit. The red line is the weighted mean age used to plot proxy data though time. The mean 95% confidence age range is 410 years, with a minimum 95% confidence error of 0.6 years at 0 cm, and a maximum 95% confidence error of 1,430 years at 335 cm.

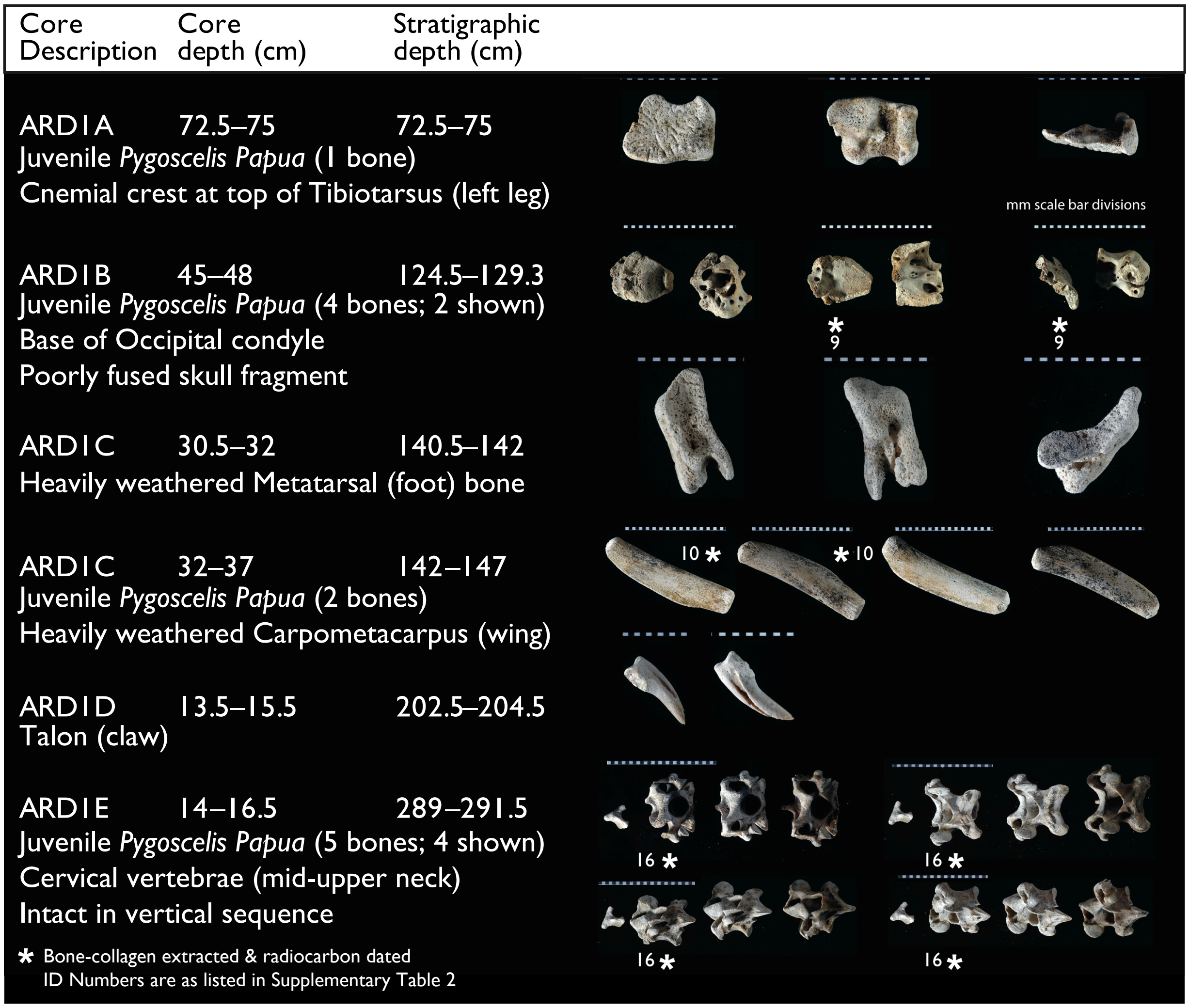

Supplementary Figure 4. Penguin bones in the Ardley Lake sediment record. Penguin bones present in the Ardley Lake record. Bone-collagen extraction and radiocarbon dating was undertaken on bones marked with an asterisk.

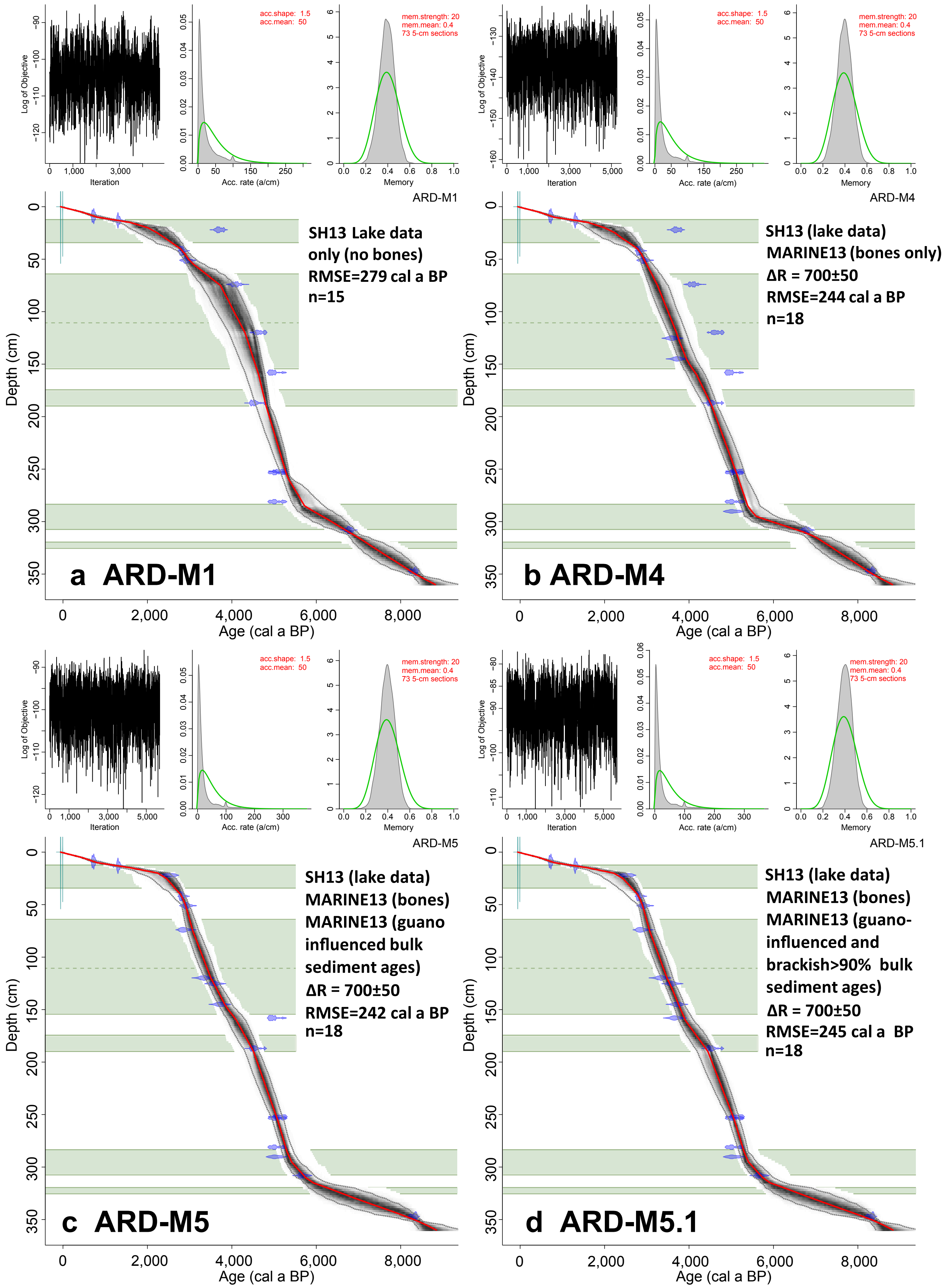

Supplementary Figure 5. Comparison of Ardley Lake age depth models ARD-M1 to ARD-M5. We investigated five age-depths modelling scenarios (ARD M1-M5) of which ARD-M1, ARD-M4 and two variations of ARD-M5 are shown. Overall, all age-depth models produce similar minimum-maximum 95% confidence basal age ranges of 8,410-9,230 cal a BP, with an average weighted mean basal age: 8,730 ± 380 cal a BP (95% confidence error).

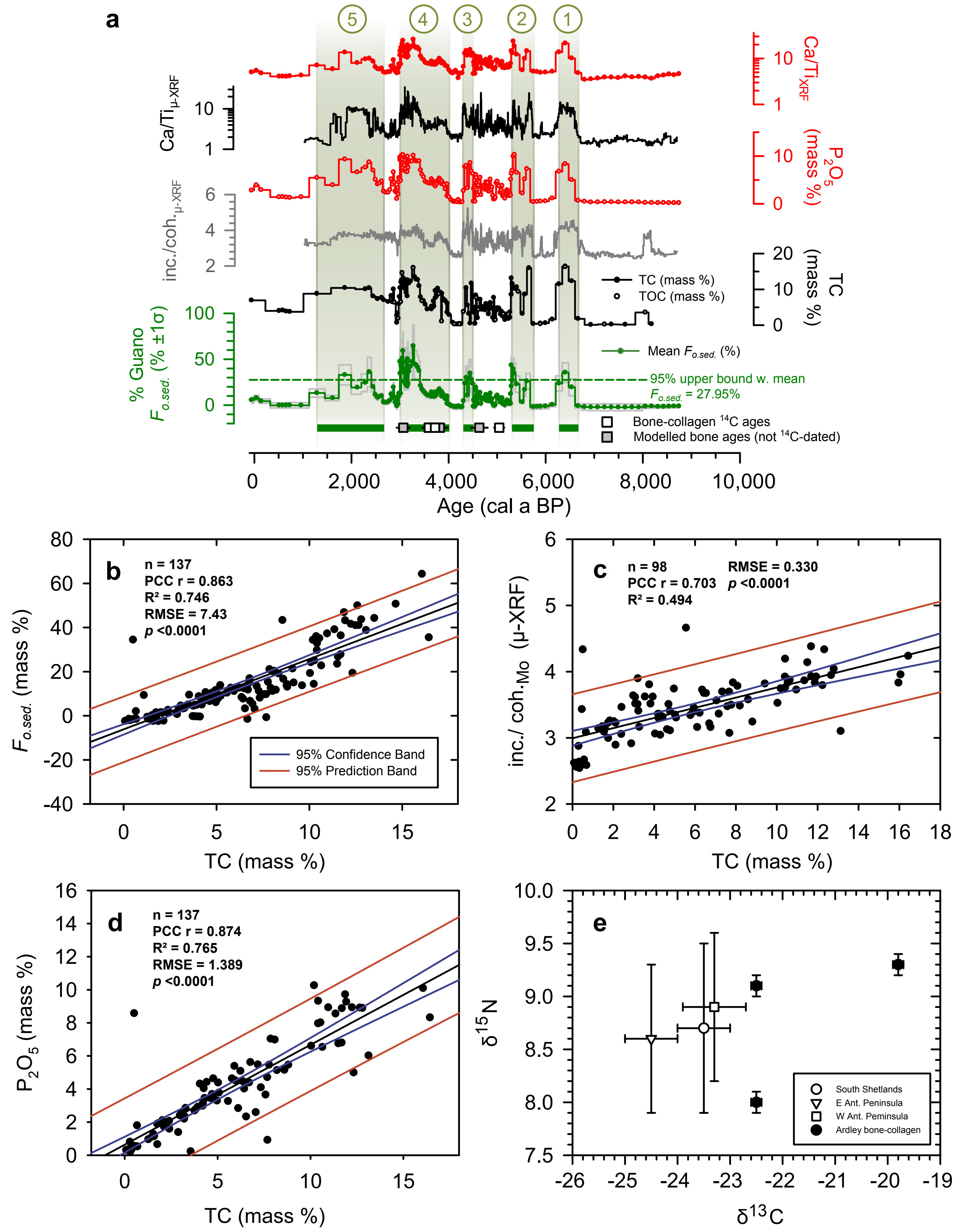

Supplementary Figure 6. Total carbon, $F_{o.sed.}$ and carbon isotope data from the ARD record. (a) Downcore comparison of (from the bottom) $F_{o.sed.}$ (green) with %C (black), inc./coh. scatter ratio, Phosphorous (as P_2O_5, red), and Ca/Ti from XRF scanning (μ-XRF) and XRF sub-sample data. **(b–d)** Regression analysis of **(b)** average $F_{o.sed.}$ (%), **(c)** μ-XRF scanning incoherent: coherent scatter ratio (inc./coh.$_{Mo}$), **(d)** P_2O_5 (mass %) and %TC data in the ARD record. **(e)** Comparison of stable Nitrogen and Carbon isotope ARD record bone-collagen data with published isotopic data from the Antarctic Peninsula[49].

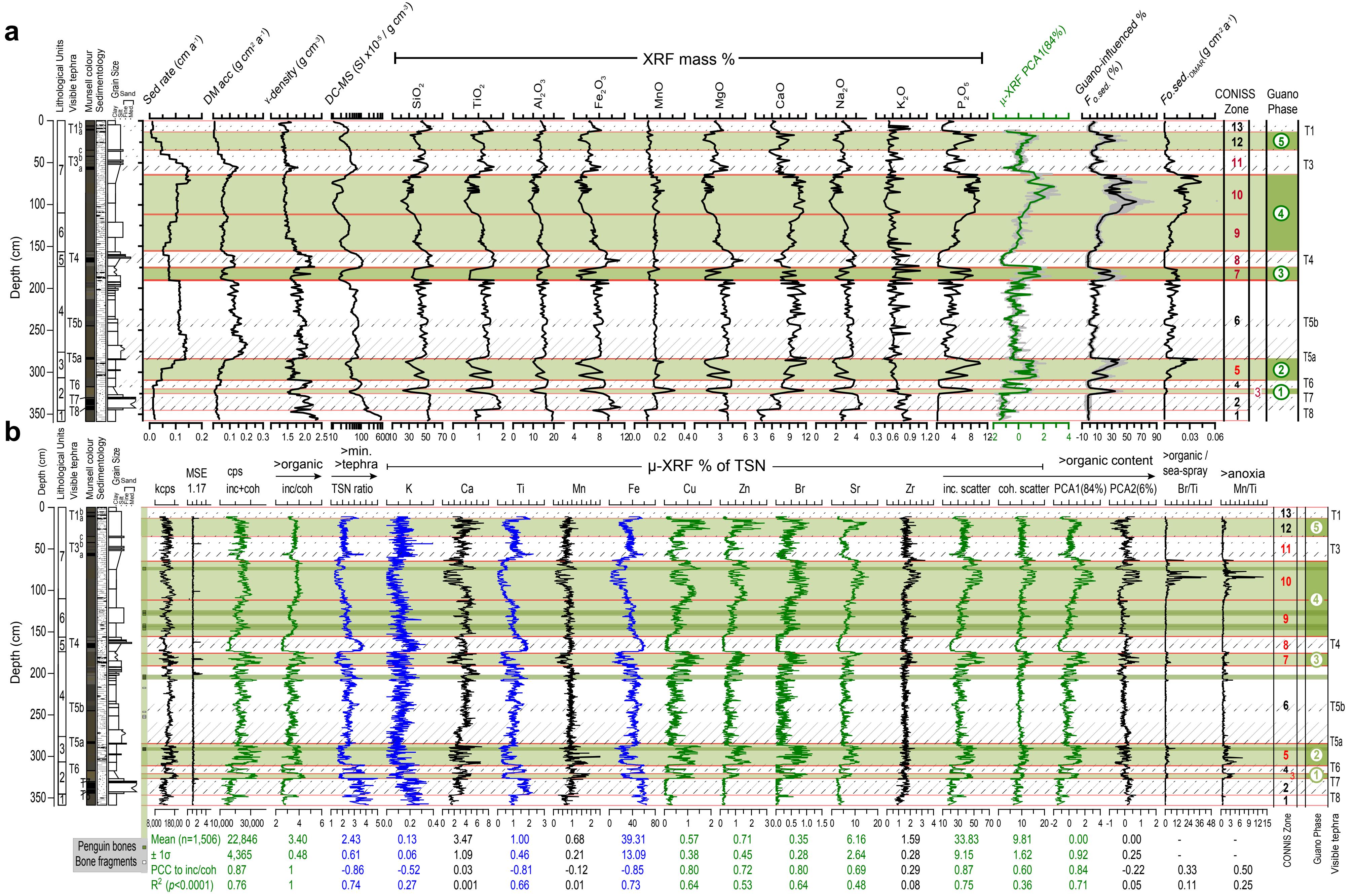

Supplementary Figure 7 (caption overpage).

Supplementary Figure 7 (previous page). Summary geochemical data for Ardley Lake sediment core record. Key variations in **(a)** XRF, GEOTEK and µ-XRF data alongside CONISS-defined guano phases. **(b)** µ-XRF data with summary interpretations. DM = dry mass accumulation rate; γ-density = GEOTEK γ-density; DC-MS = γ-density-corrected magnetic susceptibility; cps = count per second; MSE = Mean Square Error, lower values indicate better fit to theoretical spectra; inc = incoherent scatter; coh = coherent scatter; TSN = Total Scatter Normalised ratio is calculated as the sum of Element/inc+coh + inc./inc.+coh and coh./inc.+coh cps (counts per second) ratios. Since this simplifies to (Element/inc+coh) +1, it converts the influence of scatter from wet sediment scanning analysis into a constant value, and enables more effective comparison with sub-sample XRF data. Greater TSN values indicate increased mineral or tephra content of sediment and, along with coh/inc ratios, broadly correspond to changes in dry mass accumulation in most lake sediment cores[61,125]; 1-σ measurement and running mean errors are not shown for clarity, but are typically <10%; visible tephra layers are shown as diagonal lines. Increased anoxia is inferred from the Mn data[129, 130]. CONISS cluster zones are as shown in Supplementary Fig. 8, and guano-phases GP-1 to GP-5 are defined as zones with weighted mean *Fo.sed.* percentage values >10%.

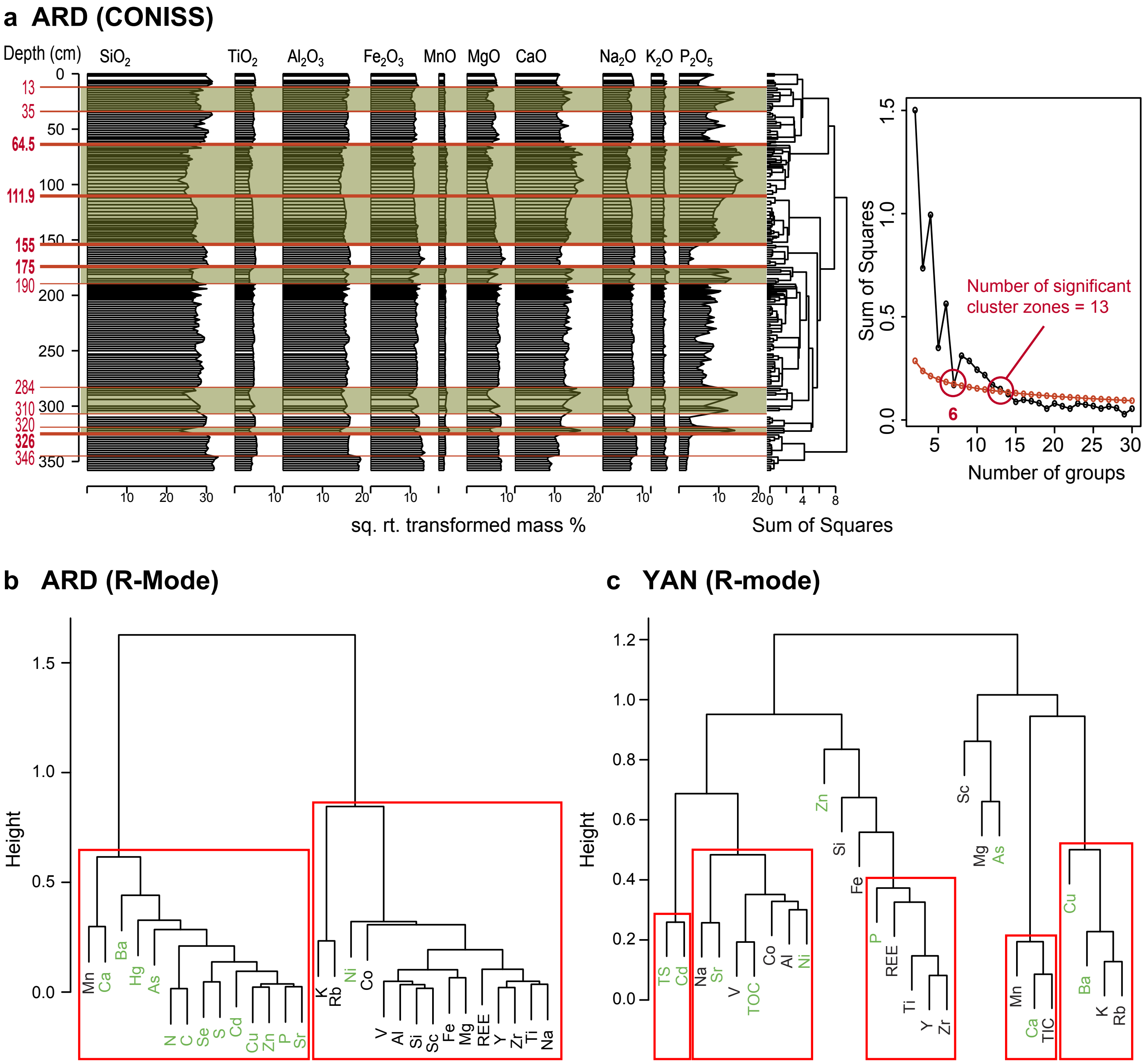

Supplementary Figure 8. CONISS and R-mode cluster analysis of the ARD geochemical record and R-mode cluster analysis of the YAN geochemical record. Results of **(a)** CONISS with broken-stick analysis applied to square root transformed XRF subsample data. Red lines show the position of the 13 significant zones with depths of the 6 most significant zones shown in bold type; **(b–c)** R-mode cluster analysis for measured major and trace elements for **(b)** the ARD sediment record and **(c)** the YAN sediment record. Clusters with *p*-values larger than 99% are highlighted by red rectangles. Major and trace elements known to be enriched in guano and ornithogenic soils are shown in green. CONISS cluster results for the YAN record are shown in Supplementary Figure 10.

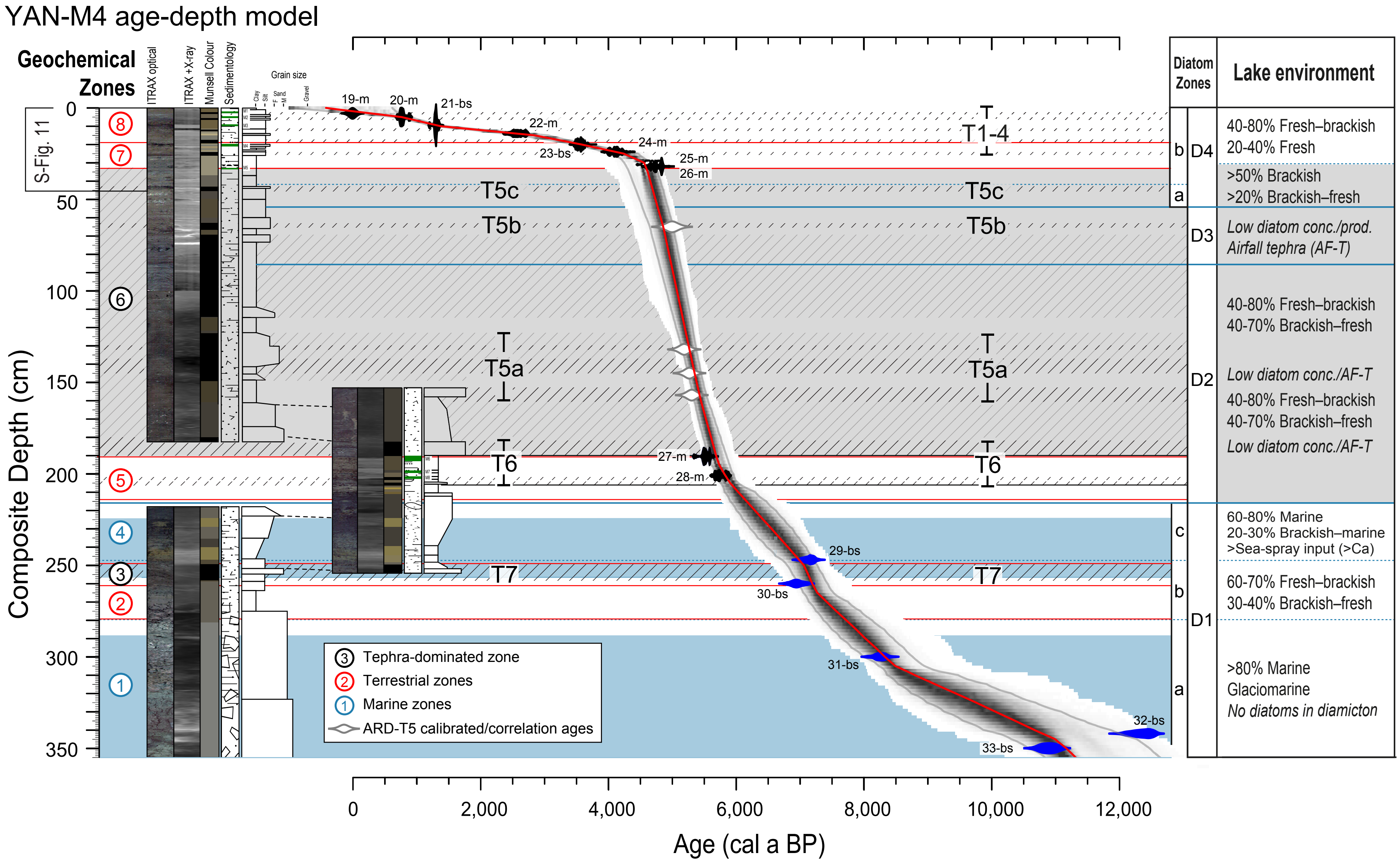

Supplementary Figure 9. Lithology & age-depth model for Yanou Lake sediment record. Lithology, age-depth model and diatom ecology zones for the Yanou Lake sediment record. Visible tephra deposits T1–7 are shown by a diagonal line shading with parts of the core containing > 50% reworked tephra deposits shaded in dark grey. Age depth modelling was undertaken in BACON v. 2.2 using SHCal13 calibration curves and run in two sections representing before (M2) and after (M1) 43–150 cm tephra deposit using the following settings for M4 (acc. shape 1.5; acc. rate mean 50 a cm^{-1}; mem. mean 0.4; mem. strength 20, producing 73 5-cm sections between 0–355 cm). The type of material dated is as described in Supplementary Table 2. The shaded grey area is a density plot of every 375thaccepted iterations of 2.4 to 16.5 million iterative curve fits; the dotted black line is the 95% confidence interval limit. The red line is the weighted mean age used to plot proxy data though time. The mean 95% confidence age range is 600 years, with a minimum 95% confidence error of 160 years at 7.7 cm, and a maximum 95% confidence error of 1,990 years at 340 cm. Legend as Supplementary Figure 3.

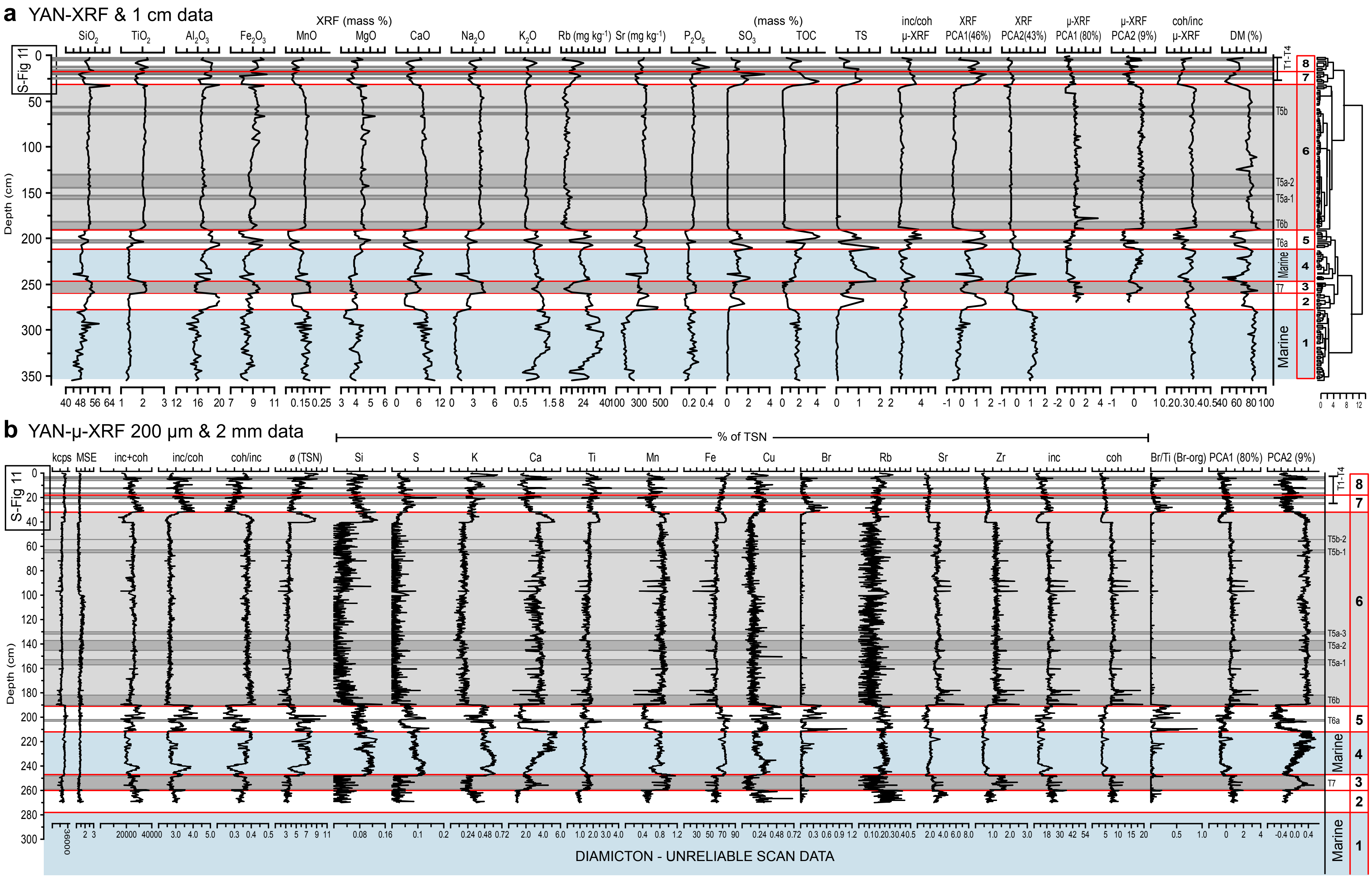

Supplementary Figure 10. Summary geochemical data for the Yanou Lake sediment core record. Key variations in **(a)** XRF and μ-XRF data alongside CONISS-defined geochemical zones. **(b)** Combined 200 μm and 2 mm μ-XRF scan data. kcps = count per second x10^3; visible tephra layers are shown as grey shaded zones and lines; see Supplementary Fig. 7 for other definitions.

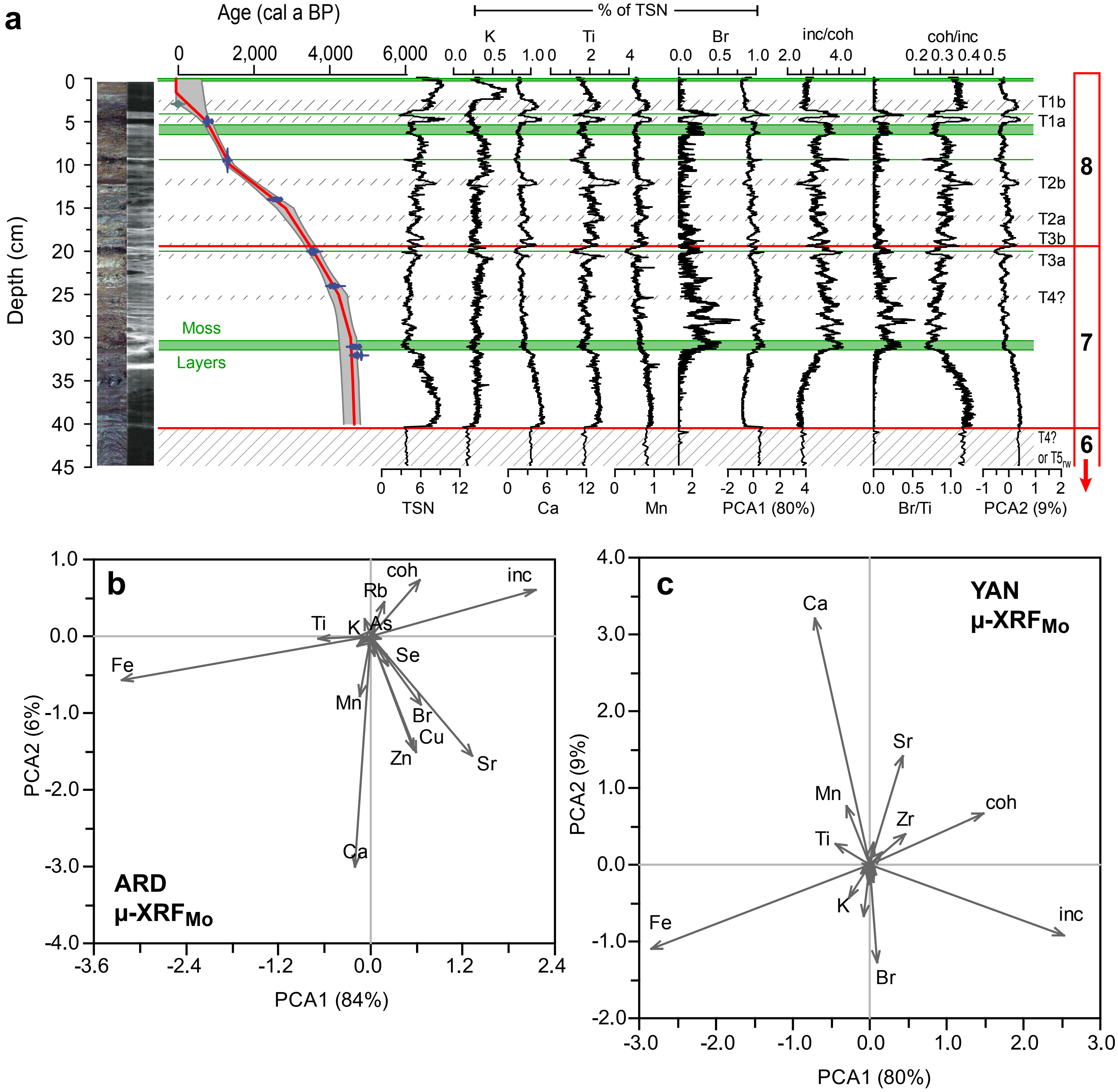

Supplementary Figure 11. Summary geochemical data for the Yanou Lake 0–45 cm sediment core record. (a) Variations in key 200 μm μ-XRF elements and ratios used to identify the position of airfall tephra deposits. PCA plots for the **(b)** ARD μ-XRF dataset and **(c)** the YAN μ-XRF dataset highlighting the main minerogenic-organic trends along the first PCA1 axis in both cores and opposite trends in, for example, Ca along PCA2 driven principally by hydroxylapatite ($Ca_5(PO_4)_3(OH)$) formation in guano-influenced sediments of the ARD record showing an affinity with biogeochemical (ornithogenic) elements Cu, Se, and Sr.

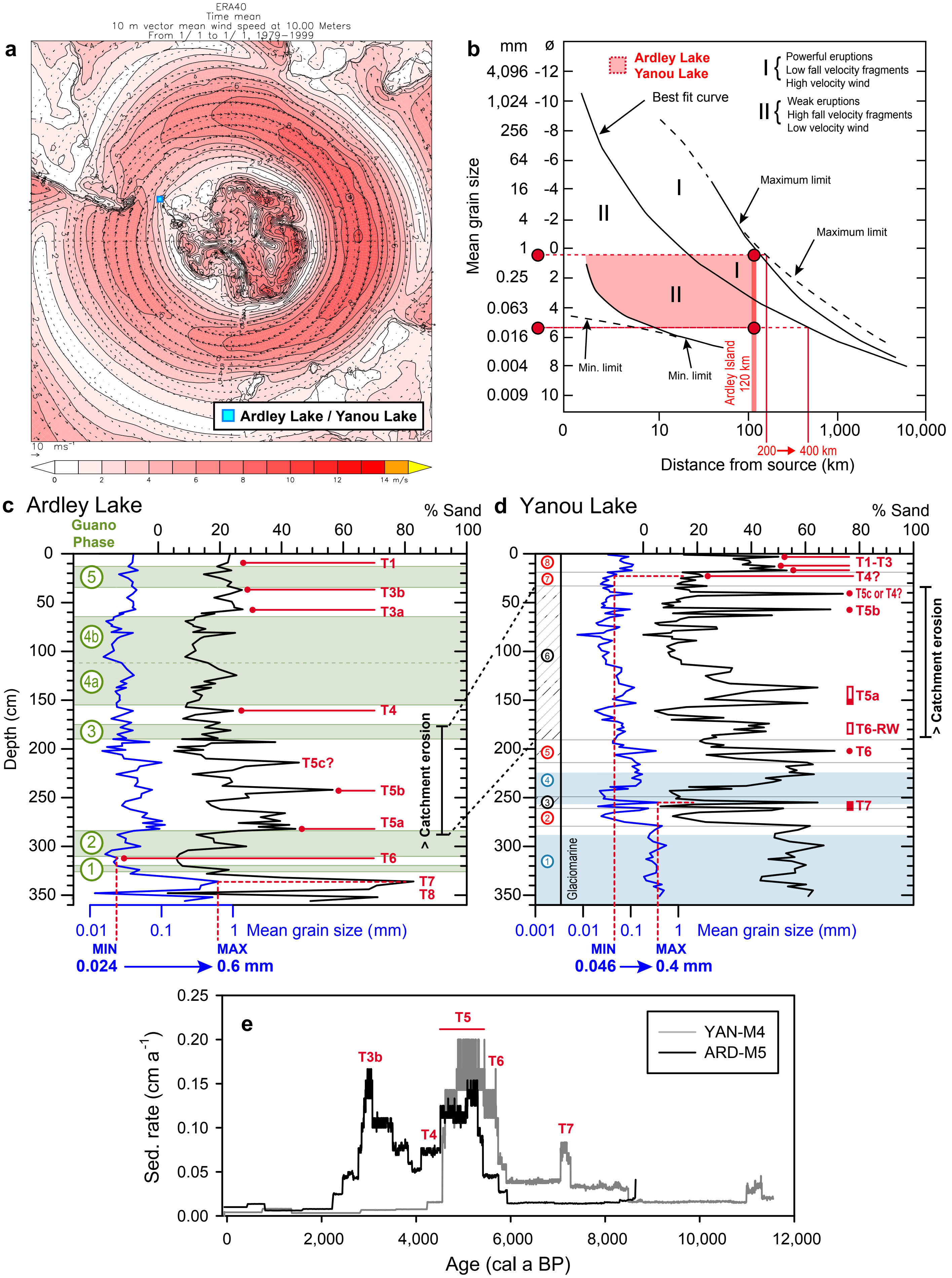

Supplementary Figure 12. Southern Hemisphere Wind circulation, eruption characteristics and grain size data for the ARD and YAN records. (a) Southern Hemisphere Westerly Wind (SWW) circulation and the location of ARD/YAN currently on the southern edge of the SWW core-belt. **(b)** Median grain size vs distance from source for various tephra deposits (adapted from[131]). **(c–d)** Grain-size variations in the ARD and YAN cores. **(e)** Comparison of 2 mm interval sedimentation rate changes in the ARD-M5 and YAN-M4 age-depth models. The red shaded area in (b) highlights that grain size of coarse-sand deposits in the ARD and YAN cores are from powerful eruptions and/or associated with high velocity winds.

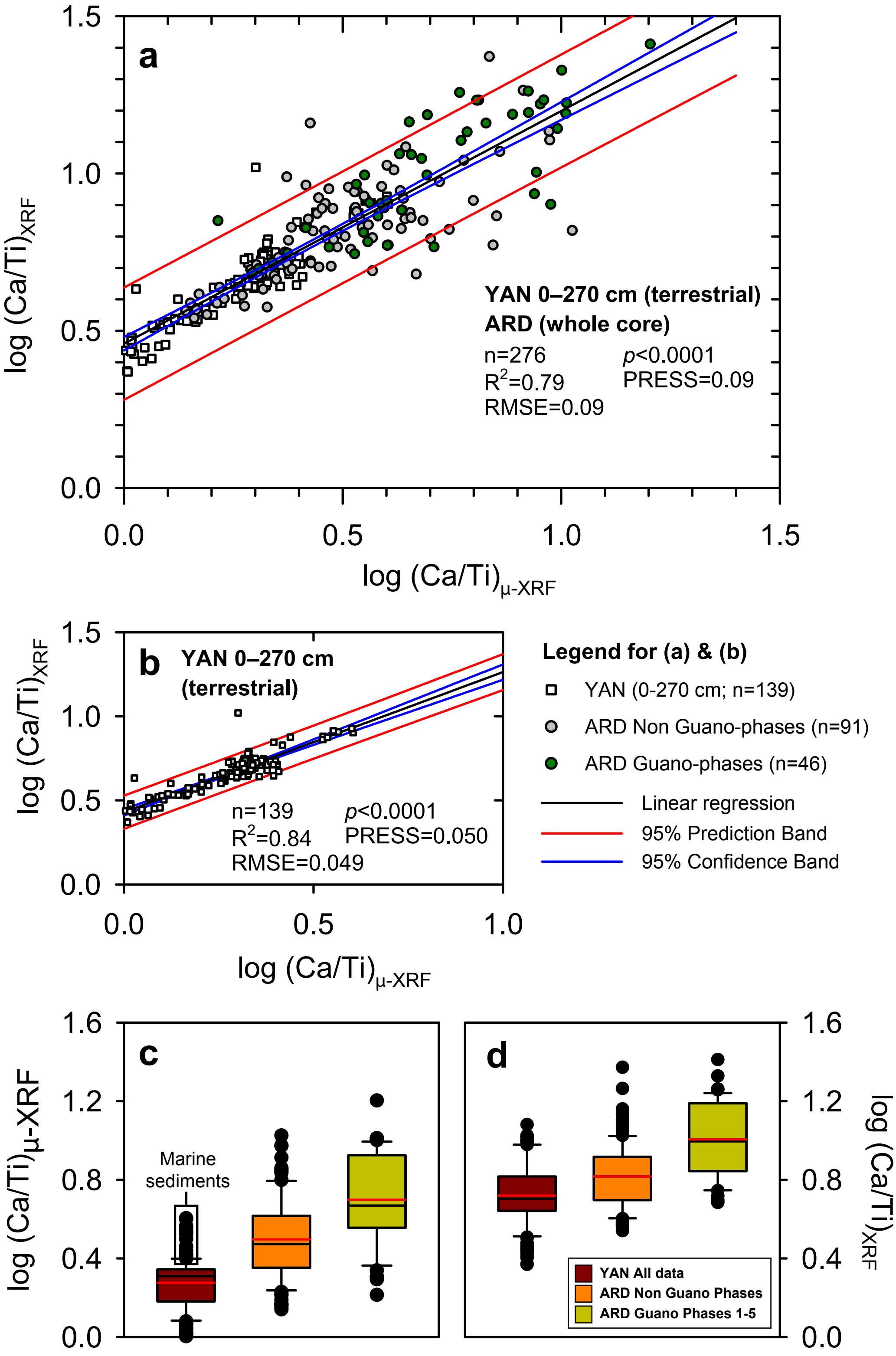

Supplementary Figure 13. Comparisons of Ca/Ti XRF and μ-XRF data from the Ardley Lake and Yanou Lake sediment records. (a, b) Regression analysis of Ca/Ti (XRF) and Ca/Ti (μ-XRF) ratio sediment geochemistry for the Yanou Lake (YAN) 0–270 cm (terrestrial sediment only) and Ardley Lake (ARD) records, highlighting the strong linear relationship between the two XRF measurement techniques used in this paper and the elevated Ca/Ti values of the ARD record. **(c, d)** Box-plots for YAN whole record (terrestrial and marine sediments), and ARD guano versus non-guano Ca/Ti ratio data for **(c)** μ-XRF and **(d)** XRF datasets. This plot shows that guano-phase sediments have significantly higher Ca/Ti ratios than YAN terrestrial and marine sediments and higher mean Ca/Ti values than non-guano phase sediments for both XRF techniques. For **(c)** and **(d)**, box boundaries represent the 25th and 75th percentile in respective datasets, error bars indicate the 10th and 90th percentiles, black lines within the boxes are median values and red lines are mean values, filled black circles are outliers beyond the 10th and 90th percentiles.

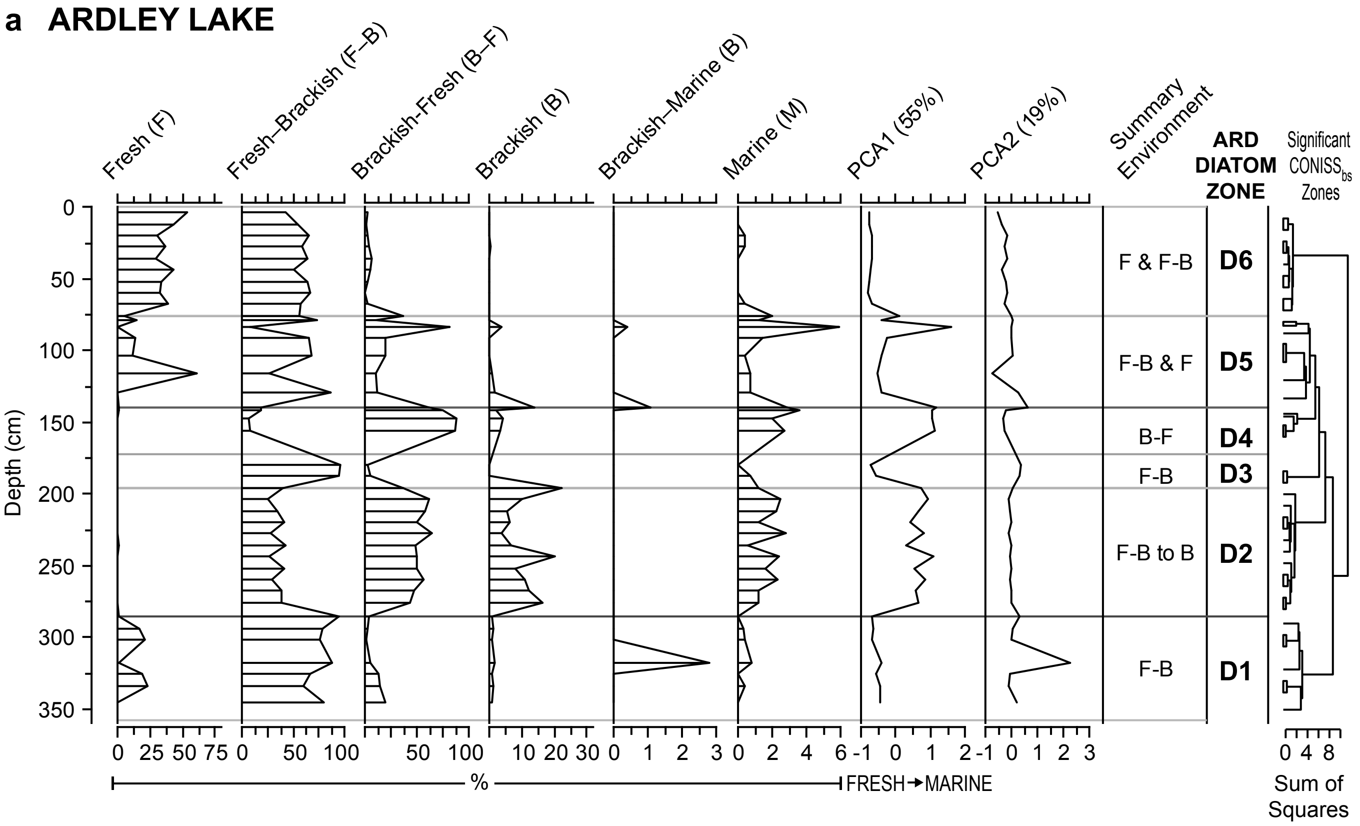

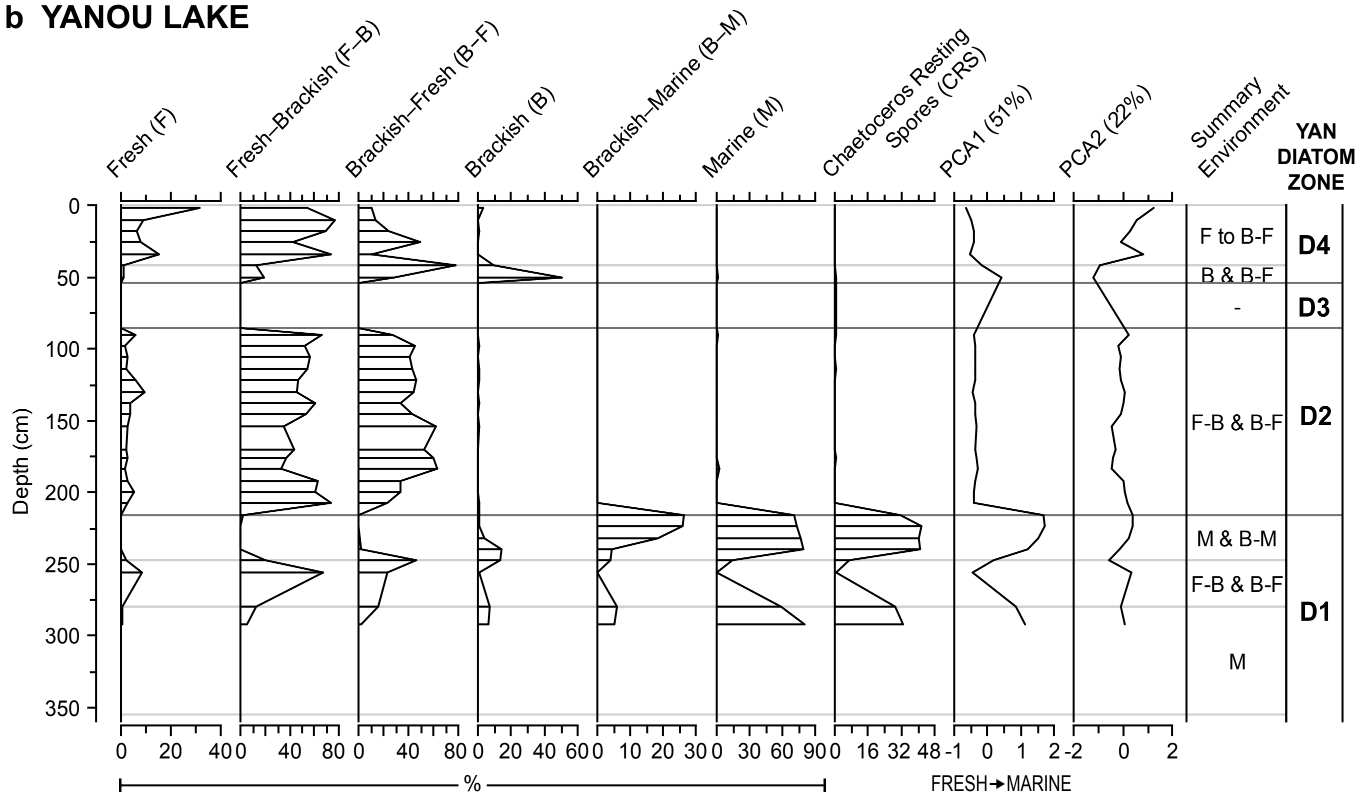

Supplementary Figure 14. Summary percentage ecological diatom assemblage preference diagrams with CONISS-defined significant zones defined for the Ardley Lake and Yanou Lake sediment records.

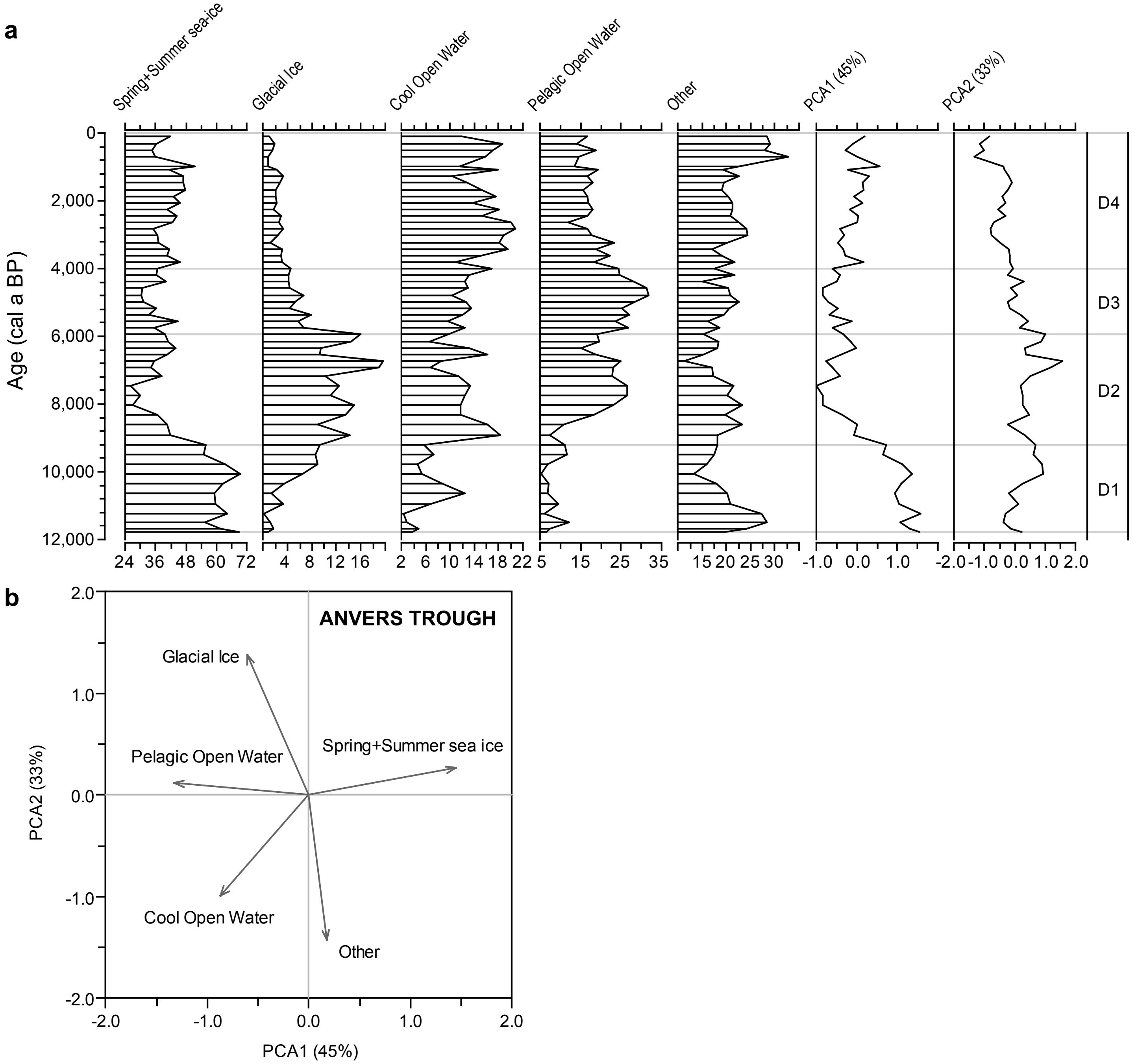

Supplementary Figure 15. Summary of Anvers Trough diatom ecological preferences. (a) Summary ecological diatom assemblages with CONISS-broken stick-defined significant zonation. **(b)** PCA plot showing the relationship between the main diatom ecological preference groups.

Supplementary Figure 16 (overpage). Proxies of penguin occupation from the Ardley Lake record compared with records of volcanic activity and reconstructed mean summer air temperature from the Yanou Lake record. In order from top: **(a)** Yanou Lake (YAN) sediment core: summary environmental reconstruction; fresh-marine diatom assemblage environmental conditions are summarised by the Principal Component Analysis PCA 1st axis plot (51% variance explained) (blue line); silt dry mass accumulation rate (DMAR), reconstructed glycerol dialkyl glycerol tetraether-mean summer average temperature (GDGT-MSAT) data (grey line), showing RMSE errors in grey shading and 100-year interval negative exponential smoothing with outliers removed (thick red line) and the upper limit (10 ^{o}C) of the calibration dataset; Ca/Ti ratio plots for XRF subsample (red line) and µ-XRF scan data (black line), 200 µm µ-XRF PCA2, (9% variance explained) (black line) and inc./coh. (organic proxy) ratio data (grey line) compared with XRF PCA1 (47% variance explained) (red line); sand DMAR data - elevated values correspond with main visible tephra deposits shown as black diagonally hatched zones; eruption events are shown as circles with 95% min.–max. confidence interval range (grey bars). **(b)** Ardley Lake (ARD) sediment core: numbered and green shaded guano phases GP-1 to GP-5, the calculated fractions of ornithogenic soils in the Ardley Lake record (*Fo.sed.* %), thick black line is the *Fo.sed.* DMAR, white squares beneath mark the location of radiocarbon-dated penguin bones (collagen extracts), grey squares are modelled ages; 2 mm µ-XRF PCA1, (84% variance explained) (black line) and inc./coh. (organic proxy) ratio data (grey line) compared with XRF PCA1 (88% variance explained) (red line); DMAR of *Pinnularia austroshetlandica*, a diatom species commonly found in shallow pools in penguin colonies (red line); fresh-brackish diatom assemblage environmental conditions are summarised by the PCA1 axis (55% variance explained) (blue line); silt dry mass accumulation rate (DMAR).

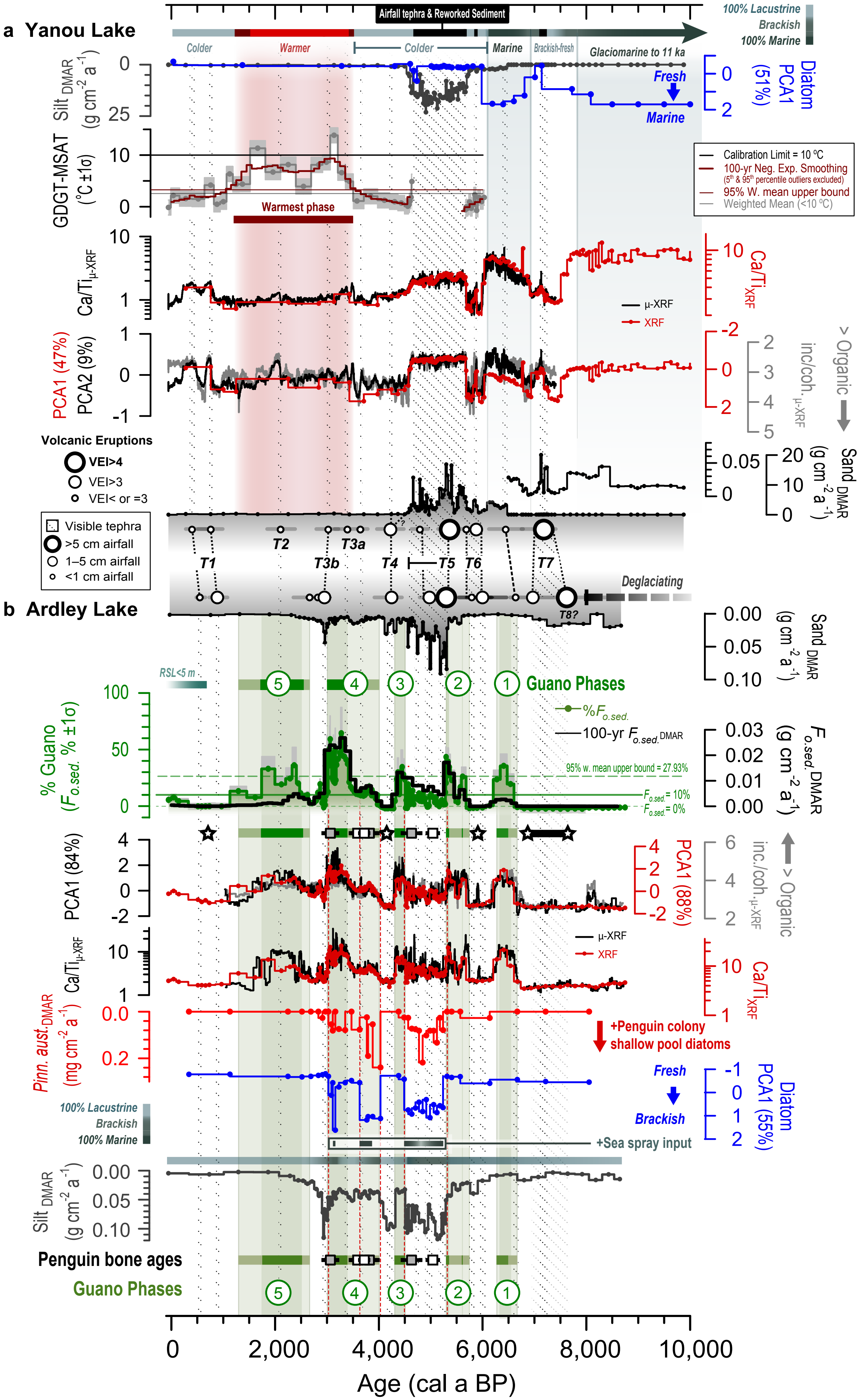

Supplementary Figure 16 (caption previous page).

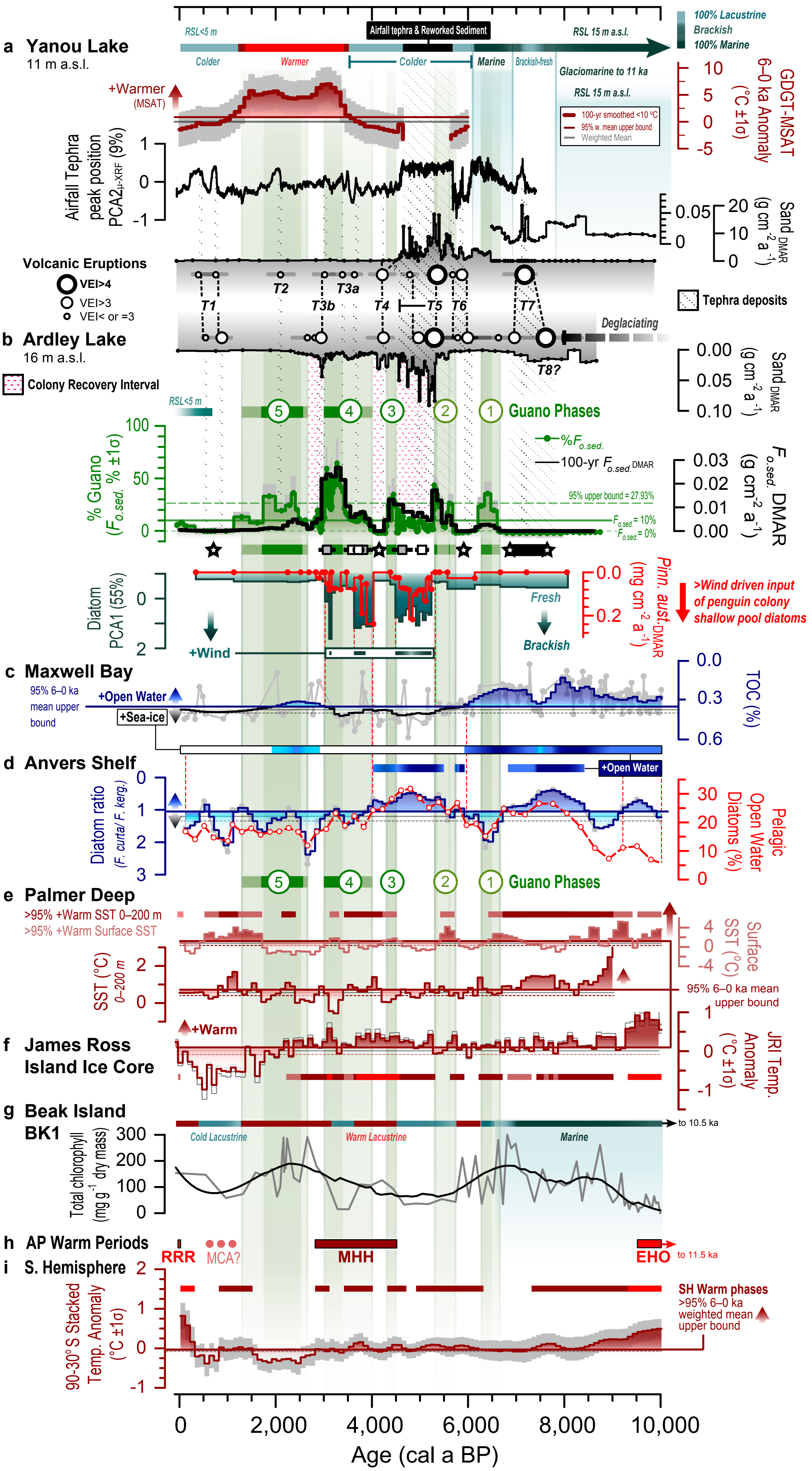

Supplementary Figure 17 (caption overpage).

Supplementary Figure 17 (previous page). Proxies of penguin occupation on Ardley Island and climate records from the Antarctic Peninsula and the Southern Hemisphere for the last 10,000 years (extended version of Figure 4). (a–f, h) as described in Figure 4 with the addition in **(b)** fresh-brackish diatom assemblage environmental conditions summarised by the PCA1 (55% variance explained) axis plot and the DMAR of *Pinnularia austroshetlandica*, a diatom species commonly found in shallow pools in penguin colonies, shown as an overlaid red line. The latter provides independent verification of penguin presence in the Ardley Lake catchment during guano phases GP-2, GP-3 and GP-4; **(g)** Beak Island Lake BK1 Total chlorophyll record[35]; **(i)** Southern Hemisphere 30–90 °S Holocene stacked temperature record[132]. See Figure 4 for full legend.

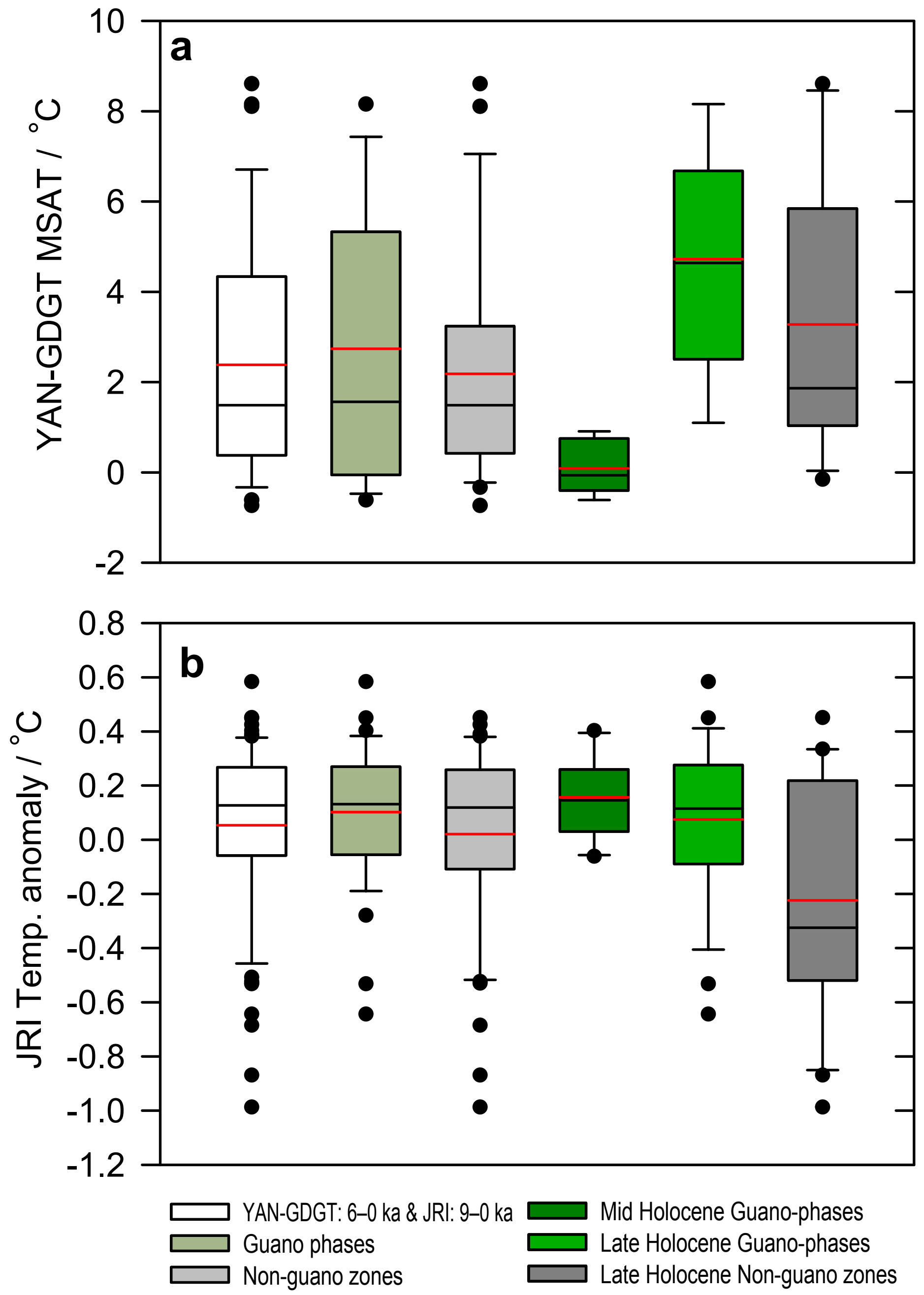

Supplementary Figure 18. Box-plots showing temperature and guano versus non-guano hypothesis testing scenarios. (a) Yanou Lake sediment reconstructed GDGT mean summer air temperature (MSAT); (b) James Ross Island ice core temperature anomaly data . For YAN-GDGT in (a), data outside of the >10 °C calibration upper limit was not included. Box boundaries represent the 25th and 75th percentile in respective datasets, error bars indicate the 10th and 90th percentiles (-0.3 °C and +8.1 °C, respectively), black lines within the boxes are median values and red lines are mean values, filled black circles are outliers beyond the 10th and 90th percentiles.

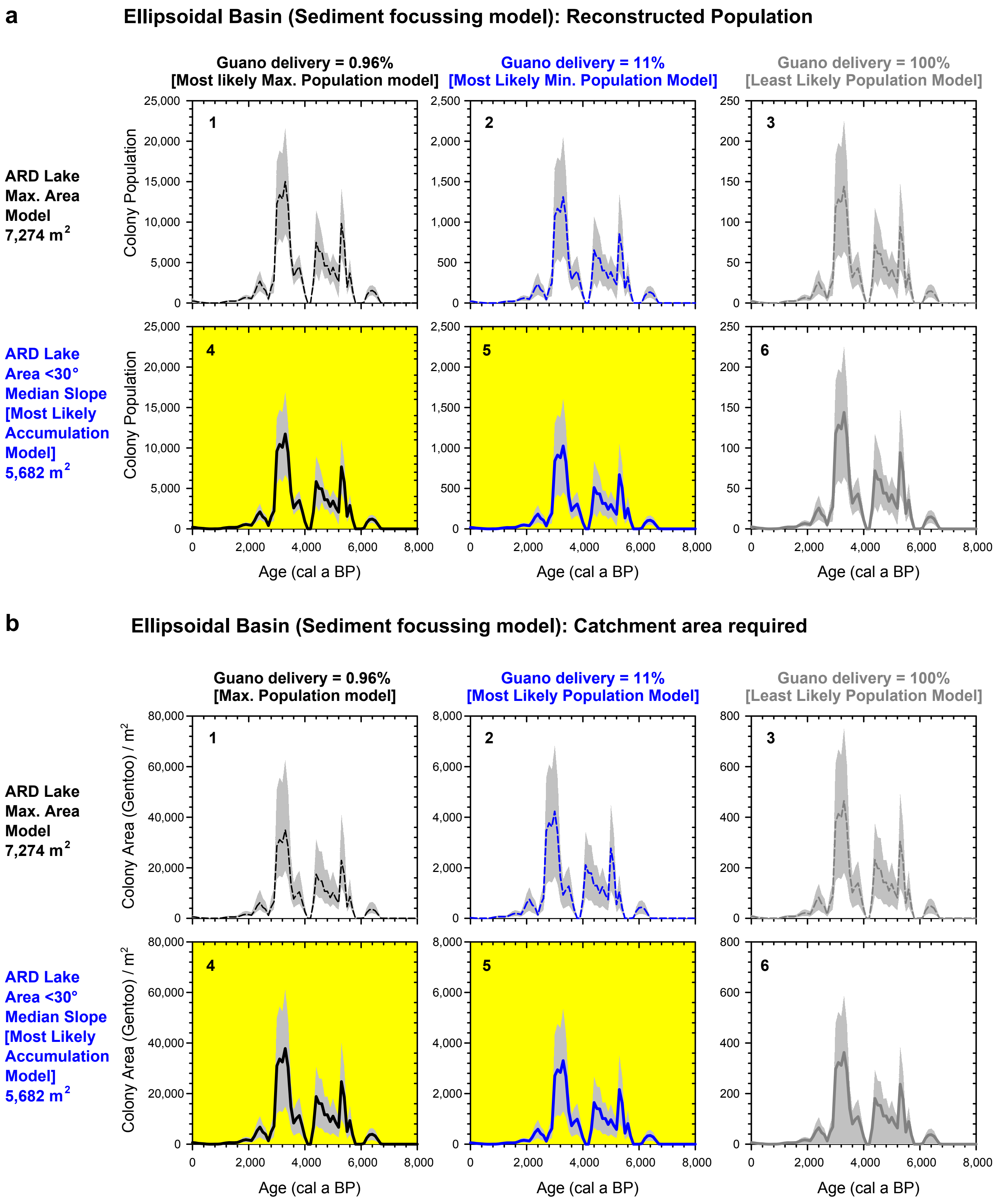

Supplementary Figure 19. Summary of key population modelling scenario results for the Ardley Lake. (a) Modelled colony population**. (b)** Corresponding area scenarios based on published gentoo penguin nesting density values. See Methods for full description of modelling and definition of terms shown in this diagram. See Supplementary Table 9 for a summary of modelling parameters.

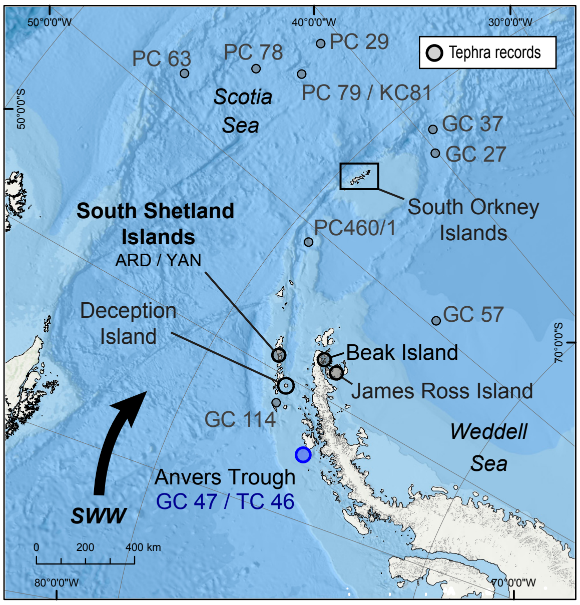

Supplementary Figure 20. Location map for Deception Island tephra analyses. Glass shards analysed from lake and marine sediment core locations in this study (YAN, ARD, Beak Island and PC460/1) were compared with published data[59] (all other sites) associated with the large late Pleistocene/early Holocene 'caldera -forming' eruption event(s). SWW indicates the main flow direction of the Southern Hemisphere Westerly Wind circulation (Supplementary Data 7, 8).

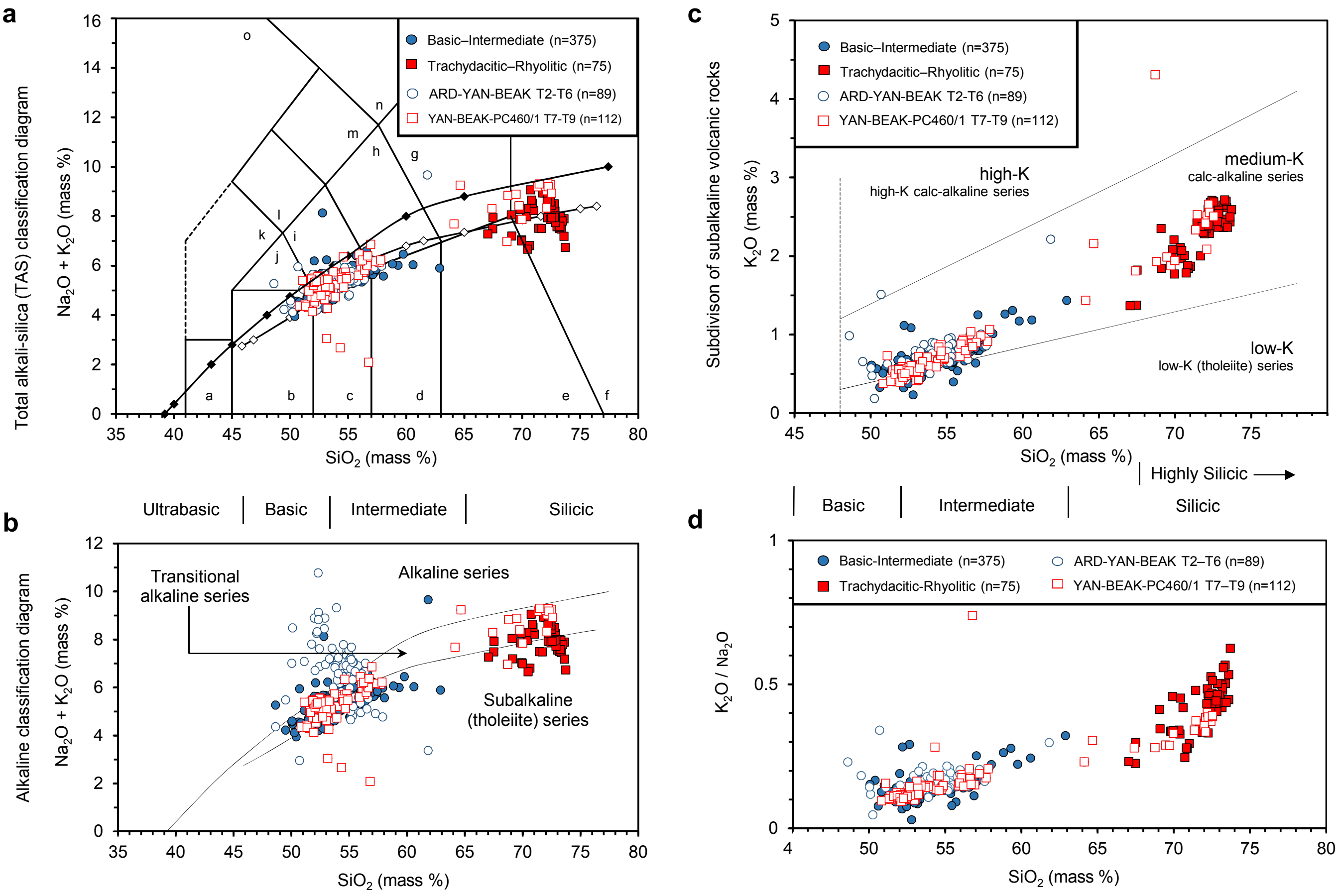

Supplementary Figure 21. Tephra-shard major element data classification diagrams. (a) Total alkali-silica (TAS) classification diagram of new shard-specific analyses comparing the major element chemical composition of T2–T6 (n=112) with T7–T9 eruptions (n=89). New data are from ARD, YAN, the Beak 1 Lake (Beak Island) and marine cores PC460/1. Published data from the Scotia Sea and Boyd Strait from Moreton and Smellie[58] were included in the underlying division Deception Island tephra into two geochemically distinct groups: Basic-Intermediate (n=375) and Trachydacitic-Rhyolitic (n=75) (Total n=450 analyses). Chemical classification and nomenclature of volcanic rocks[133] a = Picrobasalt; b = Basalt; c = Basaltic andesite; d = Andesite; e = Dacite; f = Rhyolite; g = Trachyte (normative quartz <20%) or Trachydacite (normative quartz >20%); h = Trachyandesite; i = Basaltictrachyandesite; j = Trachybasalt; k = Tephrite (normative olivine <10%) or Basanite (normative olivine >10%); l = Phonotephrite; m = Tephriphonolite; n = Phonolite; o = Foidite. General basaltic-rhyolitic magma evolution trend (solid diamond)[134]; basaltic magma evolution trend (open diamond; both based on hard-rock, rather than glass-shard data)[135]. **(b)** Subdivision of volcanic rocks into alkaline, transitional alkaline and sub-alkaline (tholeiitic) on a TAS diagram. Upper line after Irvine and Baragar[134], lower line after Kuno[135]. **(c)** Classification binary plot of K_2O vs SiO_2. Diagram is based on the subdivsion of subalkaline volcanic rocks of Le Maitre et al.[133] and Rickwood[136]. While K_2O is a mobile element in volcanic glass and this subdivision was not originally intended for this purpose, this diagram demonstrates glass-phase magma evolution relationships. **(d)** K_2O / Na_2O ratio plot. All data has been recalculated to 100% on a H_2O and CO_2 free basis. See Supplementary Methods for further experimental details and Supplementary Table 10 data summary and individual eruption data.

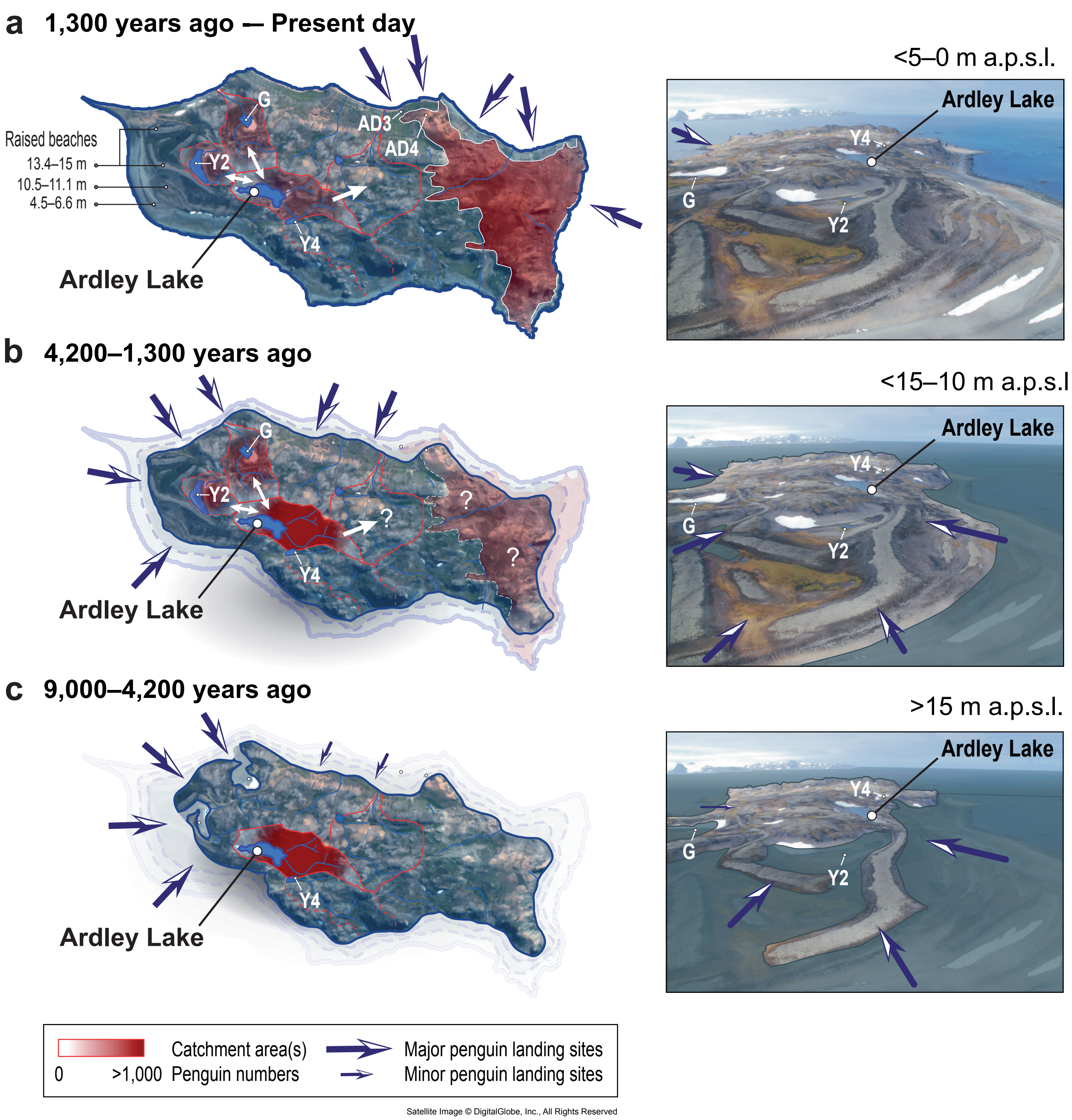

Supplementary Figure 22. Summary diagram of changes in land availability and colony population on Ardley Island. Extended version of Figure 6, with oblique angle visualisations of reconstructed sea level for stages (a)-(c). This figure includes material © DigitalGlobe, Inc., All Rights Reserved, used with permission under a NERC-BAS educational license and not included in the Creative Commons license for the article.

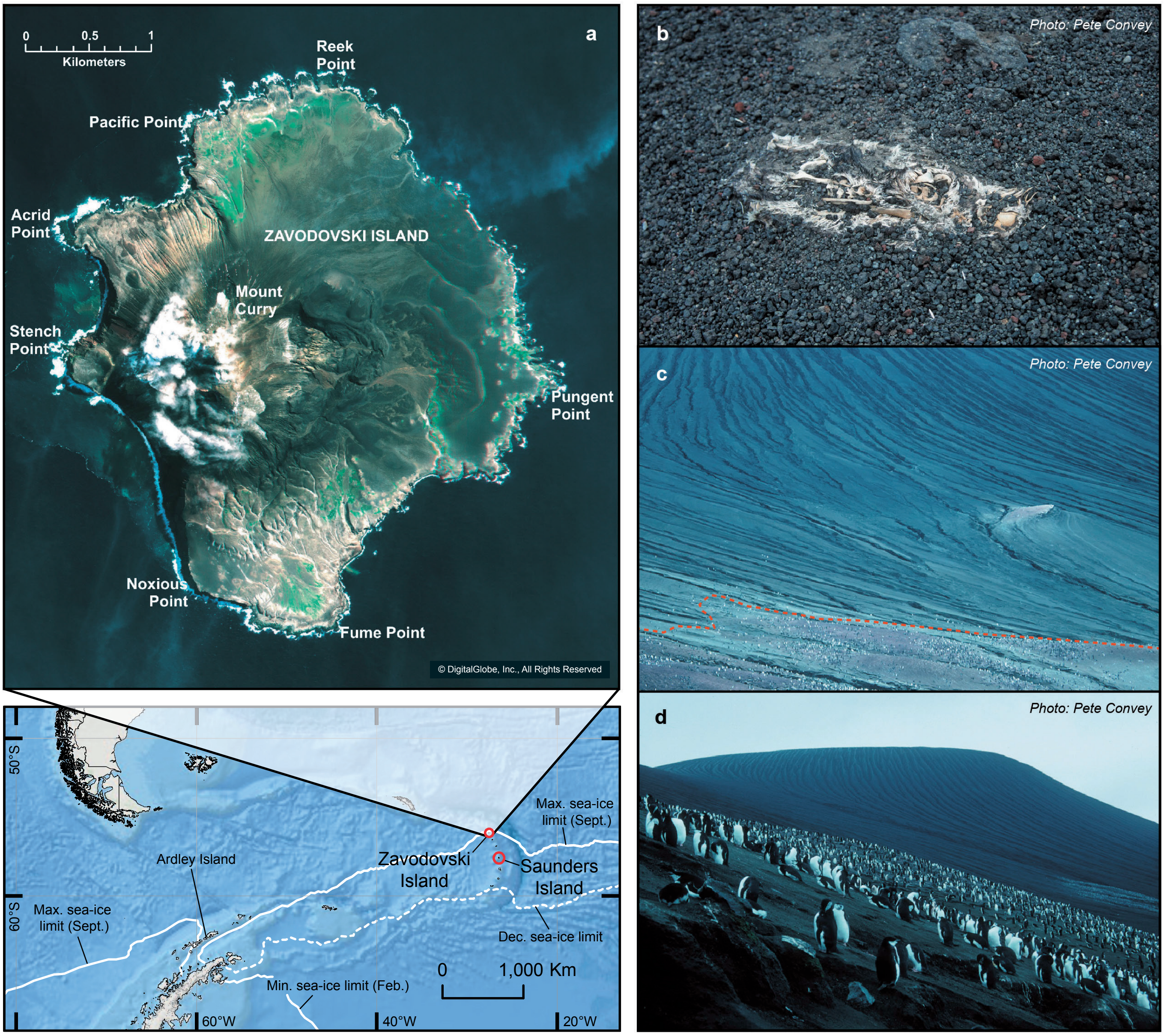

Supplementary Figure 23. Possible evidence for post-eruption impact of tephra fallout on the penguin colonies on Zavodovski Island and Saunders Island, South Sandwich Islands. (a) Location map and recent satellite image (March 4th 2011) showing proximal ash deposition on Zavodovski Island. Most of the chinstrap penguin colonies are located on the peripheries of the island, away from ash covered slopes. Sea-ice limits on the location map are as defined in Figure 1. **(b)** Remains of a chinstrap penguin in the fallout zone on Zavodovski Island (cause of death is unknown). **(c)** Chinstrap penguins on Saunders Island. The red dotted line marks the approximate boundary between penguins and recently ash-covered slopes with virtually no penguins. **(d)** Close-up of penguins in (c) also showing their absence from the most recently ash-covered slopes. This figure includes material © DigitalGlobe, Inc., All Rights Reserved, used with permission under a NERC-BAS educational license and not included in the Creative Commons license for the article. Pictures (b-d) were taken by Pete Convey in January 1997, used with permission.

Supplementary Table 1. Bioelements present in guano and guano-influenced sediments from Antarctica. Comparison of bioelements found in ornithogenic sediments and penguin guano around Antarctica. *n.d.*=not determined.

Location	Sample type	Cu	P	S	As	Se	Zn	Cd	F	Sr	Ca	Mg	Ni	Ba	Hg
Lake Boeckella, Hope Bay[5]	ornithogenic sediments	x	x			*n.d.*	x	x	*n.d.*	x	x				*n.d.*
Seabee Hook, Cape Hallett, northern Victoria Land[6]	penguin guano	x	x	x	x		x	x			x	x		*n.d.*	*n.d.*
Zolotov Island, Vestfold Hills[2]	ornithogenic sediments	x	x	x	*n.d.*	*n.d.*		x		*n.d.*		x		*n.d.*	*n.d.*
Gardner Island, Vestfold Hills[7]	ornithogenic sediments	x	x	x	x	x		*n.d.*	x	x					*n.d.*
Beaufort Island & Cape Bird, Ross Sea region[1]	ornithogenic sediments	x	x	x	x	x	x	x	*n.d.*						*n.d.*
Lake Y2, Ardley Island, South Shetland Islands[3]	ornithogenic sediments	x	x	x		x	x	*n.d.*	x	x	x			x	*n.d.*
Lake Y4, Ardley Island, South Shetland Islands[16]	ornithogenic sediments	x	x	x		x	x	*n.d.*	x	x	x				*n.d.*
Mochou Lake, Larsemann Hills, East Antarctica[8]	ornithogenic sediments	x	x		x	x	x	x	x				x		
Ardley Lake, Ardley Island, South Shetland Islands (this study)	ornithogenic sediments	x	x	x	x	x	x	x	*n.d.*	x	x				x

Supplementary Table 2. Radiocarbon age data for sediment cores analysed in this study. Radiocarbon ages for **(a)** the Ardley Lake sediment core, **(b)** the Yanou Lake sediment core and **(c)** marine cores TC046/GC047 from the Anvers Trough referred to in this paper, showing conventional radiocarbon ages, marine reservoir corrected ages (in blue) and 2σ calibrated age data. Pre-treatment: A = acid; AAA = acid-alkali-acid; AW = acid washes; * = estimated isotopic values due to small samples size; Calibration curves: A = SHCal13(SHZ1-2); B = SHCal13; C = MARINE13 with ΔR = 700 ± 50 ^{14}C a (100% marine); D = MARINE13 with ΔR = 1,470 ± 10 ^{14}C a (100% marine) (see Supplementary Methods for details).

ID	Laboratory & Sample Codes	Core section: Core depth (cm)	Stratigraphic depth (cm) Min - Max	CONISS-Strat. Zone	Guano Zone	Material dated & carbon source	Pre-treatment	Carbon content (%)	$\delta^{13}C_{VPDB}$ (‰ ± 0.1)	Measured [Absolute] pMC (% modern ± 1σ)	Conventional Radiocarbon Age (^{14}C years BP ± 1σ)	Calibrated age (cal a BP) [A-B: SH13; C-D: MAR13] 95.4% (2σ-error) Max. - Min. range	Calibrated Mean±1σ	Calibarted Median	Cal. Curve
**(a) Ardley Lake, South Shetland Islands**															
1	SUERC-22305	ARD-SUR: 0-1	0 – 1	13	-	Living *Drepanocladus longifolius* (Mitt.) Paris aquatic moss	AAA	13.2	-23.8	104.23 ± 0.48 [103.49 ± 0.48]	modern	-6.3 – -7.8 [1956.3-1957.8 CE]	-7.1 ± 1.06	-7	A
2	BETA-338226	ARD-SUR: 9-10	9 – 10	13		*Drepanocladus longifolius* (Mitt.) Paris aquatic moss layer	AAA	-	-25.6	89.85 ± 0.34	860 ± 30	775 – 675	725 ± 30	725	B
3	BETA-338227	ARD1A:13-14	13 – 14	12	5	*Drepanocladus longifolius* (Mitt.) Paris aquatic moss strands	AAA	-	-25.0*	83.59 ± 0.42	1,440 ± 40	1,370 – 1,190	1,305 ± 35	1,305	
4	BETA-271284	ARD1A:22-23	22 – 23	12	5	Organic-rich sediment (moss free)	AW	10.4*	-25.7	64.84 ± 0.32	3,480 ± 40	2,690 – 2,344	2,520 ± 97	2,521	C
5	BETA-271285	ARD1A:42-43	42 – 43	11	-	Organic-rich sediment (moss free)	AW	8.2*	-23.6	71.01 ± 0.35	2,750 ± 40	2,920 – 2,745	2,815 ± 40	2,810	B
6	BETA-271287	ARD1A:51-52	51 – 52	11	-	Organic-rich sediment (moss free)	AW	-	-24.0	70.04 ± 0.35	2,860 ± 40	3,065 – 2,795	2,925 ± 65	2,920	B
7	BETA-271286	ARD1A:74-75	74 – 75	10	4	Organic-rich sediment (moss free)	AW	-	-25.8	62.47 ± 0.31	3,780 ± 40	3,022 – 2,727	2,862 ± 77	2,855	C
8	SUERC-22315	ARD1B:42-43(B)	119.8 – 121.4	9	4	Mineral-rich sediment (no macrofossils present in zone)	AW	4.4	-26.4	59.61 ± 0.26	4,155 ± 36	3,476 – 3,160	3,325 ± 79	3,329	C
9	BETA-380310	ARD1B:45-46(B)	124.5 – 126.1	9	4	Bone collagen extracted	A	-	-22.5	58.00 ± 0.20	4,380 ± 30	3,765 – 3,435	3,593 ± 81	3,592	C
10	BETA-380311	ARD 1C 33-37	143.0 – 147.0	9	4	Bone collagen extracted	A	-	-22.5	57.30 ± 0.20	4,480 ± 30	3,875 – 3,563	3,720 ± 80	3,720	C
11a	SUERC-18922	ARD1C:48-49	158 – 159	8	-	Mineral-rich sediment (no macrofossils) - SH13 calibrated	AW	0.6	-25.9	57.59 ± 0.27	4,433 ± 37	5,270 – 4,850	4,975 ± 95	4,960	B
11b						Deposited in >90% brackish-fresh lake - MAR13 calibrated						3,830 – 3,488	3,663 ± 86	3,660	C
12	SUERC-22307	ARD1C:77-78	187 – 188	7	3	Unidentifiable moss strands - terrestrial origin	AW	30.8	-23.9	60.09 ± 0.27	4,091 ± 36	4,800 – 4,420	4,545 ± 95	4,525	B
13	SUERC-22316	ARD1D:63-64	252 – 253	6	-	Mineral-rich sediment (paired bulk-macrofossil ages)	AW	9.0	-9.2	56.99 ± 0.26	4,517 ± 37	5,300 – 4,970	5,140 ± 100	5,145	B
14	SUERC-22308	ARD1D:63.5-64.5	253.5 – 254.5	6	-	Yellow-green lacustrine algae - terrestrial origin	AW	47.2	-22.3	57.23 ± 0.27	4,484 ± 38	5,285 – 4,875	5,085 ± 115	5,060	B
15	SUERC-22309	ARD1D:92-93	281 – 282	6	-	Yellow-green lacustrine algae - terrestrial origin	AW	48.5	-20.5	57.33 ± 0.27	4,469 ± 38	5,280 – 4,865	5,050 ± 120	5,020	B
16	BETA-378927	ARD1E:14-16.5(B)	289 – 291.5	5	2	Bone collagen extracted	A	-	-19.8	50.60 ± 0.20	5,470 ± 30	5,235 – 4,858	5,043 ± 103	5,035	C
17	SUERC-18948	ARD1E:33-34	308 – 309	4	2	Organic-rich sediment (no macrofossils)	AW	13.3	-25.5	47.44 ± 0.21	5,990 ± 35	5,840 – 5,540	5,659 ± 70	5,653	C
18	SUERC-22317	ARD1E:72-73	347 – 348	1		Mineral-rich sediment (no macrofossils)	AW	4.1	-18.2	39.06 ± 0.19	7,552 ± 39	8,405 – 8,200	8,320 ± 55	8,335	B
**(b) Yanou Lake, South Shetland Islands**															
19	SUERC-22325	YANSC:1.5-3	1.5 – 3	8		Living *Drepanocladus longifolius* aquatic moss	AAA	1.7	-16.7	104.53 ± 0.46 [103.79 ± 0.46]	modern	-6.3 – -7.8 [1956.3-1957.8 CE]	-7.1 ± 1.1	-7	A
20	SUERC-18934	YAN8A-1:5-5.5	5 – 5.5	8		*Drepanocladus longifolius* aquatic moss	AW	10	-25.0*	89.32 ± 0.41	907 ± 37	905 – 680	775 ± 50	765	B
21	BETA-316286	YAN8A-1:9.5-10	9.5 – 10	8		*Drepanocladus longifolius* aquatic moss	AAA	-	-25.3	83.69 ± 0.31	1,430 ± 30	1,355 – 1,265	1,300 ± 25	1,300	B
22	BETA-271289	YAN8A-1:14-15	14 – 15	8		Bulk organic sediment (lake)	AW	-	-15	72.89 ± 0.36	2,540 ± 40	2,745 – 2,380	2,580 ± 90	2,580	B
23	BETA-316287	YAN8A-1:20-20.5	20 – 20.5	7		*Drepanocladus longifolius* aquatic moss	AAA	-	-29	65.74 ± 0.25	3,370 ± 30	3,640 – 3,455	3,555 ± 50	3,560	B
24	BETA-271290	YAN8A-1:24-25	24 – 25	7		Bulk organic sediment (lake)	AW	-	-16.6	62.39 ± 0.31	3,790 ± 40	4,245 – 3,970	4,105 ± 75	4,105	B
25	BETA-316288	YAN8A-1:31-31.5	31 – 31.5	7		*Campylium polygamum* moss layer	AAA	-	-23.2	59.50 ± 0.22	4,170 ± 30	4,820 – 4,525	4,675 ± 85	4,670	B
26	SUERC-18935	YAN8A-1:32-32.5	32 – 32.5	7		*Campylium polygamum* moss layer	AW	20	-25.0*	58.75 ± 0.27	4,273 ± 37	4,870 – 4,620	4,760 ± 75	4,750	B
27	SUERC-22310	YAN9B-1:37.5-38	190.5 – 191	6		*Campylium polygamum* ? moss layer	AAA	39.5	31.9	54.69 ± 0.25	4,847 ± 37	5,645 – 5,330	5,525 ± 55	5,520	B
28	SUERC-22311	YAN9B-1:48-49	201 – 202	5		*Campylium polygamum* ? moss strands	AAA	13.6	-26.5	53.33 ± 0.25	5,051 ± 38	5,895 – 5,645	5,760 ± 75	5,745	B
29	SUERC-22326	YAN8B-1:29-30	247 – 248	4		Bulk organic sediment (marine-influenced)	AW	1.7	-20.7	40.17 ± 0.19	7,326 ± 37	7,285 – 6,981	7,144 ± 78	7,153	C
30	SUERC-22327	YAN8B-1:42-43	260 – 261	3		Bulk organic sediment (marine)	AW	0.3	-27.1	41.04 ± 0.19	7,155 ± 38	7,135 – 6,785	6,951 ± 90	6,949	C
31	BETA-271291	YAN8B-1:82-83	300 – 301	1		Bulk organic sediment (marine)	AW	-	-25.9	34.71 ± 0.30	8,500 ± 70	8,421 – 8,053	8,258 ± 89	8,261	C
32	BETA-271292	YAN8B-2:24-25	342 – 343	1		Mineral-rich sediment (marine)	AW	-	-24.2	23.69 ± 0.21	11,570 ± 70	12,595 – 12,064	12,350 ± 146	12,367	C
33	BETA-271293	YAN8B-2:32-33	350 – 351	1		Mineral-rich sediment (marine)	AW	-	-25.3	26.63 ± 0.23	10,630 ± 70	11,125 – 10,667	10,893 ± 122	10,894	C
**(c) Anvers Shelf marine cores**															
34	OxA-3249_0	TC04601: 0	0 – 1	-		Bulk sediment (marine)	AW	-	-24.8	-	1,870 ± 70	250 – -	110 ± 80	90	D
35	OxA-3250_281	GC04702: 241-242	281 – 282	-		Bulk sediment (marine)	AW	-	-34.9	-	7,780 ± 90	7,070 – 6,480	6,780 ± 140	6,780	D
36	SUERC-15254_400	GC04703: 360-361	400 – 401	-		Bulk sediment (marine)	AW	0.76	-22.8	20.04 ± 0.19	12,768 ± 76	13,010 – 12,590	12,790 ± 100	12,780	D
37	OxA-3251_419	GC04704: 379-380	419 – 420	-		Bulk sediment (marine)	AW	-	-22.8	-	12,280 ± 150	12,670 – 11,650	12,210 ± 260	12,240	D

Supplementary Table 3. Eruption ages for visible ash layers in the Ardley Lake and Yanou Lake sediment records compared to published ages. Ages in this study were primarily compared with eruption ages from the James Ross Island (JRI) ice core[28] and composite ages in recent publications [34, 35, 47, 52, 59, 60, 137].

AGE BRACKET (cal ka BP)	Ash layer		ARD-M5 Age (cal a BP)		YAN-M4 Age (cal a BP)		JRI & AP Airfall tephras: most likely			AP VEI>3
	ID	Sub-eruption	95% conf. min. – max.	Weighted mean	Weighted mean	95% conf. min. – max.	ID	W. Mean Age cal a BP	95% CI Age min. – max.	Composite Age*
0–1	**T1**	b	480 – 656	549	398	277* – 757	JRI-6?	691	686 – 791	450
		a	730 – 1,083	886	759	512 – 990	JRI-8	910	810 – 1,010	750
1–2	**T2**				2,105	1,846 – 2,385	JRI-15	2,330	2,130 – 2,530	2,100/2,250
2–3	**T3**	b	2,351 – 2,826	2,655						2,500
			2,600 – 2,901	2,815	3,024	2,792 – 3,222	JRI-16	2,670	2,470 – 2,890	
			2,833 – 3,074	2,950						2,700
		a			3,393	3,147 – 3,651	JRI-17	3,300	3,080 – 3,500	3,500
					3,676	3,591 – 3,786	JRI-18	3,800	3,600 – 4,000	
3–4	**T4**		3,993 – 4,444	4,230	4,222	3,961 – 4,481	JRI-19<br>JRI-20	3,910<br>4,100	3,710 – 4,110<br>3,900 – 4,600	-
		MOSS	4,620 - 4,870	4,760	4,760	4,620 – 4,870				
4–5	**T5**	b	4,787 – 5,141	4,974	4,822	4,588 – 4,950	JRI-21 GB-3 BB-1 AP14 Bk-Td	5,040	4,720 – 5,150	4,700
		a-2	5,056 – 5,410	5,218	5,302	5,141 – 5,452				5,200
		a-1	5,176 – 5,460	5,301	5,413	5,301 – 5,534		5,550	5,350 – 5,750	
		MOSS	5,330 – 5,645	5,525	5,525	5,330 – 5,645				
5–6	**T6**	b	5,515 – 6,145	5,791	5,687	5,563 – 5,853	MS-1	5,550	5,350 – 5,750	5,550
		MOSS	5,645 – 5,895	5,760	5,760	5,645 – 5,895				
		a	5,692 – 6,461	5,997	5,878	5,720 – 5,916	BB-1	-	5,000 – 6,000	6,000
6–7	**T7**	c	6,012 – 7,357	6,637	6,445	6,120 – 6,780	JRI-?	No airfall tephra 7–10 ka		Glacially-reworked?
		b	6,241 – 7,683	6,968	7,094	6,750 – 7,412				
7–8	**T7a or T8?**		6,535 – 8,374	7,621	**Glaciomarine Sediments**		MS-2	8,285	8,085 – 8,485	8,700?

Supplementary Table 4. Geochemical correlation matrix for the Ardley Lake sediment core. Correlation coefficients between the elements in Ardley Lake sediments. Bold values mark correlation coefficients r > 0.9 and r < -0.9; ** Correlation is significant at the 0.01 level (2-tailed); * Correlation is significant at the 0.05 level (2-tailed).

	Si	Ti	Al	Fe	Mn	Mg	Ca	Na	K	P	As	Ba	Cd	Co	Cu	Hg	Ni	Sc	Se	Sr	V	Y	Zn	Zr	∑REE	TC	TS	TN
**Si**	1	0.87 **	**0.95** **	0.84 **	-0.44 **	0.87 **	-0.64 **	0.88 **	0.49 **	**-0.96** **	-0.73 **	-0.43 **	**-0.91** **	0.69 **	**-0.95** **	-0.78 **	0.62 **	**0.98** **	**-0.98** **	**-0.95** **	**0.93** **	0.82 **	**-0.94** **	0.81 **	0.83 **	**-0.94** **	-0.85 **	**-0.95** **
**Ti**		1	0.78 **	0.86 **	-0.16 *	0.87 **	-0.35 **	**0.96** **	0.27 **	-0.87 **	-0.63 **	-0.72 **	-0.86 **	0.63 **	**-0.92** **	-0.77 **	0.65 **	0.73 **	-0.84 **	-0.84 **	0.85 **	**0.96** **	-0.86 **	**0.96** **	**0.91** **	**-0.92** **	-0.75 **	-0.85 **
**Al**			1	0.86 **	-0.39 **	0.89 **	-0.62 **	0.82 **	0.49 **	**-0.94** **	-0.70 **	-0.35 **	-0.84 **	0.75 **	**-0.90** **	-0.79 **	0.67 **	**0.98** **	**-0.94** **	-0.90 **	**0.95** **	0.73 **	**-0.90** **	0.72 **	0.79 **	**-0.94** **	-0.85 **	**-0.93** **
**Fe**				1	-0.05	**0.93** **	-0.41 **	0.87 **	0.39 **	**-0.90** **	-0.51 **	-0.55 **	-0.84 **	0.70 **	-0.87 **	-0.80 **	0.70 **	**0.93** **	-0.89 **	-0.84 **	**0.92** **	0.82 **	-0.85 **	0.85 **	0.68 **	-0.85 **	-0.69 **	-0.84 **
**Mn**					1	-0.09	0.70 **	-0.11	-0.15 *	0.33 **	0.53 **	-0.12	0.23	-0.12	0.29 **	0.41 **	0.03	-0.39	0.28	0.37 **	-0.27 **	-0.07	0.31 **	-0.05	-0.39	0.30 **	0.36 **	0.34 **
**Mg**						1	-0.33 **	0.88 **	0.34 **	**-0.91** **	-0.62 **	-0.63 **	-0.89 **	0.73 **	**-0.91** **	-0.79 **	0.84 **	**0.95** **	**-0.92** **	-0.85 **	**0.92** **	0.81 **	-0.88 **	0.84 **	0.79 **	-0.90 **	-0.83 **	-0.89 **
**Ca**							1	-0.33 **	-0.38 **	0.63 **	0.50 **	-0.10	0.45	-0.37 **	0.55 **	0.48 **	-0.03	-0.73 **	0.70 **	0.70 **	-0.57 **	-0.37 **	0.59 **	-0.28 **	-0.62 **	0.47 **	0.41 **	0.54 **
**Na**								1	0.40 **	-0.88 **	-0.62 **	-0.64 **	-0.85 **	0.64 **	-0.89 **	-0.79 **	0.69 **	0.87 **	-0.88 **	-0.84 **	0.86 **	**0.93** **	-0.85 **	**0.95** **	0.89 **	**-0.92** **	-0.79 **	-0.86 **
**K**									1	-0.40 **	-0.17 *	0.28 **	-0.42	0.31 **	-0.32 **	-0.18	0.30 **	0.67 **	-0.57 **	-0.40 **	0.43 **	0.30 **	-0.36 **	0.31 **	0.43	-0.41 **	-0.47 **	-0.43 **
**P**										1	0.67 **	0.51 **	0.90 **	-0.66 **	**0.97** **	0.80 **	-0.64 **	**-0.97** **	**0.95** **	**0.98** **	**-0.95** **	-0.82 **	**0.97** **	-0.82 **	-0.81 **	0.89 **	0.81 **	**0.91** **
**As**											1	0.43 **	0.83 **	-0.49 **	0.68 **	0.51 **	-0.46 **	**-0.92** **	**0.95** **	0.64 **	-0.62 **	-0.57 **	0.63 **	-0.54 **	-0.84 **	0.74 **	0.72 **	0.63 **
**Ba**												1	0.38	-0.30 **	0.61 **	0.64 **	-0.59 **	-0.14	0.40	0.46 **	-0.43 **	-0.67 **	0.53 **	-0.71 **	-0.32	0.57 **	0.53 **	0.42 **
**Cd**													1	-0.75 **	**0.94** **	0.48 **	-0.66 **	-0.89 **	**0.91** **	**0.91** **	**-0.91** **	-0.77 **	**0.91** **	-0.81 **	-0.76 **	0.50 **	0.77	0.68 *
**Co**														1	-0.63 **	-0.42 **	0.63 **	0.82 **	-0.80 **	-0.61 **	0.72 **	0.60 **	-0.56 **	0.60 **	0.73 **	-0.73 **	-0.51 **	-0.71 **
**Cu**															1	0.79 **	-0.64 **	**-0.91** **	**0.96** **	**0.95** **	**-0.93** **	-0.86 **	**0.98** **	-0.87 **	-0.78 **	0.87 **	0.82 **	0.90 **
**Hg**																1	-0.63 **	-0.67	0.74	0.72 **	-0.75 **	-0.71 **	0.75 **	-0.76 **	-0.28	0.72 **	0.74 **	0.73 **
**Ni**																	1	0.76 **	-0.70 **	-0.56 **	0.65 **	0.61 **	-0.61 **	0.66 **	0.67 **	-0.76 **	-0.62 **	-0.69 **
**Sc**																		1	**-0.96** **	**-0.96** **	**0.98** **	0.73 **	**-0.92** **	0.66 *	0.82 **	-0.71 *	**-0.98** **	-0.78 **
**Se**																			1	**0.97** **	**-0.94** **	-0.82 **	**0.94** **	-0.75 **	-0.80 **	0.66 *	**0.95** **	0.74 **
**Sr**																				1	**-0.92** **	-0.81 **	**0.96** **	-0.79 **	-0.89 **	0.85 **	0.78 **	0.89 **
**V**																					1	0.79 **	**-0.93** **	0.79 **	0.77 **	**-0.92** **	-0.79 **	**-0.93** **
**Y**																						1	-0.81 **	**0.98** **	**0.92** **	-0.87 **	-0.73 **	-0.80 **
**Zn**																							1	-0.81 **	-0.76 **	0.83 **	0.79 **	0.87 **
**Zr**																								1	0.86 **	-0.90 **	-0.73 **	-0.81 **
**∑REE**																									1	-0.74 **	**-0.99** **	-0.65 **
**TC**																										1	**0.91** **	**0.98** **
**TS**																											1	0.87 **
**TN**																												1

Supplementary Table 5. Geochemical correlation matrix for the Yanou Lake sediment record. Correlation coefficients between the elements in Yanou Lake sediments. Bold values mark correlation coefficients r > 0.9 and r < -0.9; ** Correlation is significant at the 0.01 level (2-tailed); * Correlation is significant at the 0.05 level (2-tailed).

	Si	Ti	Al	Fe	Mn	Mg	Ca	Na	K	P	As	Ba	Cd	Co	Cu	Ni	Sc	Sr	V	Y	Zn	Zr	∑REE	TC	TS
**Si**	1	0.65 **	-0.05	0.29 **	0.54 **	0.46 **	0.57 **	0.67 **	-0.49 **	0.37 **	-0.49 **	-0.49 **	-0.68 *	0.27 **	-0.54 **	0.32 **	0.31	0.74 **	-0.34 **	0.44 **	0.07	0.58 **	0.18	-0.55 **	-0.50 **
**Ti**		1	-0.62 **	0.50 **	0.88 **	0.48 **	0.82 **	**0.90** **	-0.77 **	0.71 **	-0.74 **	-0.76 **	-0.45	0.21 *	-0.84 **	0.35 **	0.48	0.82 **	-0.57 **	0.75 **	0.04	**0.92** **	0.53	-0.88 **	-0.72 **
**Al**			1	-0.12	-0.75 **	-0.23 *	-0.71 **	-0.65 **	0.65 **	-0.20 *	0.62 **	0.79 **	-0.16	0.24 *	0.73 **	-0.44 **	-0.27	-0.51 **	0.75 **	-0.57 **	0.12	-0.73 **	-0.45	0.55 **	0.49 **
**Fe**				1	0.34 **	0.48 **	0.15	0.31 **	-0.18	0.48 **	0.04	-0.16	-0.63	0.56 **	-0.24 *	0.00	-0.16	0.21 *	0.08	0.25 *	0.19 *	0.38 **	-0.50	-0.53 **	-0.39 **
**Mn**					1	0.66 **	**0.94** **	**0.94** **	-0.71 **	0.37 **	-0.81 **	**-0.93** **	-0.63 *	0.02	**-0.91** **	0.61 **	0.34	0.85 **	-0.79 **	0.74 **	-0.05	**0.96** **	0.35	**-0.91** **	-0.89 **
**Mg**						1	0.53 **	0.59 **	-0.30 **	0.09	-0.36 **	-0.55 **	-0.63	0.34 **	-0.50 **	0.50 **	-0.15	0.50 **	-0.30 **	0.38 **	0.10	0.56 **	-0.37	-0.59 **	-0.72 **
**Ca**							1	**0.94** **	-0.80 **	0.28 **	-0.82 **	**-0.95** **	-0.51	-0.10	**-0.94** **	0.61 **	0.30	**0.92** **	-0.85 **	0.66 **	-0.09	0.90 **	0.33	-0.88 **	-0.83 **
**Na**								1	-0.80 **	0.41 **	-0.83 **	**-0.90** **	-0.61	0.05	**-0.93** **	0.49 **	0.37	**0.93** **	-0.73 **	0.68 **	-0.02	**0.93** **	0.30	**-0.93** **	-0.85 **
**K**									1	-0.43 **	0.60 **	0.80 **	0.14	-0.10	0.84 **	-0.28 **	-0.60	-0.80 **	0.60 **	-0.50 **	0.05	-0.71 **	-0.70 *	0.76 **	0.58 **
**P**										1	-0.30 **	-0.19	-0.21	0.41 **	-0.38 **	-0.12	0.44	0.35 **	0.01	0.40 **	0.12	0.45 **	0.52	-0.42 **	-0.17
**As**											1	0.77 **	0.33	0.16	0.79 **	-0.49 **	-0.43	-0.79 **	0.75 **	-0.62 **	0.06	-0.79 **	-0.48	0.86 **	0.82 **
**Ba**												1	0.41	0.12	**0.93** **	-0.60 **	-0.22	-0.87 **	0.87 **	-0.63 **	0.10	-0.88 **	-0.22	0.84 **	0.81 **
**Cd**													1	-0.28	0.54	-0.24	-0.07	-0.55	0.09	0.15	-0.31	-0.52	0.34	**0.96** **	**0.93** **
**Co**														1	-0.04	-0.16	0.28	0.04	0.34 **	-0.04	0.23 *	0.03	0.13	-0.14	-0.03
**Cu**															1	-0.47 **	-0.35	**-0.91** **	0.80 **	-0.57 **	0.07	-0.88 **	-0.29	**0.92** **	0.83 **
**Ni**																1	0.17	0.47 **	-0.66 **	0.44 **	-0.08	0.58 **	0.07	-0.55 **	-0.60 **
**Sc**																	1	0.36 **	-0.08	0.36	0.33	0.37	0.74 *	-0.50	-0.16
**Sr**																		1	-0.75 **	0.58 **	-0.03	0.84 **	0.32	-0.88 **	-0.80 **
**V**																			1	-0.58 **	0.17	-0.76 **	-0.20	0.71 **	0.68 **
**Y**																				1	0.00	0.81 **	0.87	-0.56 **	-0.51 **
**Zn**																					1	-0.06	-0.25	0.01	0.02
**Zr**																						1	0.41	-0.89 **	-0.81 **
**∑REE**																							1	-0.18	0.10
**TC**																								1	0.89 **
**TS**																									1

Supplementary Table 6. Correlation matrices for Mean $F_{o.sed.}$ data versus (a) key 1 cm interval XRF and µ-XRF data (b) CNS (mass%) sub-sample data. TSN = Total Scatter Normalised ratio is calculated as the sum of Element/inc+coh + inc./inc.+coh and coh./inc.+coh cps (counts per second) based ratios (which simplifies to (Element/inc+coh) +1). Greater TSN values indicate increased mineral or tephra content of sediment and, along with coh/inc ratios broadly correlate with changes in dry mass accumulation in most lake sediment cores[62,128].

**a)** Variables (1 cm sub-sample interval matches to 5-pt running mean µ-XRF scan data)	n	min. –max.	Mean ± 1σ	**Mean $F_{o.sed.}$ (mass%)**	**TC (mass%)**	**TN (mass%)**	**TS (mass%)**	**P_2O_5 (mass%)**	**Ca/Ti XRF**	**inc/coh (µ-XRF)**	**TSN ratio (µ-XRF)**	**PCA1 (84.2%)**	**Br/Ti (µ-XRF)**	**Mn/Ti (µ-XRF)**	**Ca/Ti (µ-XRF)**
				**Correlation matrix (Pearson r-values) (all p<0.0001):**											
**Mean $F_{o.sed.}$ (mass %)**	143	-2.41 – 64.38	11.02 ± 14.09	**1**	**see b**	**see b**	**see b**	**0.694**	**0.667**	**0.564**	**-0.501**	**0.593**	**0.617**	**0.486**	**0.584**
**TC (mass%)**	98	0.07 – 16.43	5.75 ± 4.23	**see b**	**1**	**0.985**	**0.916**	**0.874**	**0.830**	**0.703**	**-0.666**	**0.696**	**0.633**	**0.629**	**0.727**
**TN (mass%)**	100	0.07 – 2.14	0.76 ± 0.49	**see b**	**0.970**	**1**	**0.894**	**0.899**	**0.864**	**0.718**	**-0.688**	**0.734**	**0.678**	**0.612**	**0.748**
**TS (mass%)**	64	0.12 – 2.03	0.69 ± 0.49	**see b**	**0.839**	**0.799**	**1**	**0.849**	**0.791**	**0.646**	**-0.539**	**0.604**	**0.563**	**0.660**	**0.619**
**P_2O_5 (mass%)**	137	0.19 – 10.27	3.83 ± 2.83	**0.482**	**0.765**	**0.808**	**0.721**	**1**	**0.904**	**0.735**	**-0.736**	**0.809**	**0.701**	**0.589**	**0.748**
**Ca/Ti XRF**	137	3.47 – 25.80	8.45 ± 4.33	**0.446**	**0.689**	**0.746**	**0.626**	**0.816**	**1**	**0.651**	**-0.622**	**0.714**	**0.709**	**0.673**	**0.769**
**inc/coh (µ-XRF)**	143	2.52 – 4.66	3.40 ± 0.46	**0.318**	**0.494**	**0.516**	**0.417**	**0.541**	**0.423**	**1**	**-0.887**	**0.880**	**0.653**	**0.642**	**0.691**
**TSN ratio (µ-XRF)**	143	1.45 – 4.19	2.44 ± 0.58	**0.251**	**0.443**	**0.473**	**0.291**	**0.541**	**0.387**	**0.787**	**1**	**-0.941**	**-0.662**	**-0.572**	**-0.646**
**PCA1 (84.2%)**	143	-1.54 – 2.22	-0.03 ± 0.85	**0.352**	**0.484**	**0.539**	**0.365**	**0.655**	**0.510**	**0.774**	**0.886**	**1**	**0.832**	**0.683**	**0.774**
**Br/Ti (µ-XRF)**	143	0.00 – 5.31	0.62 ± 0.84	**0.381**	**0.400**	**0.459**	**0.317**	**0.491**	**0.502**	**0.426**	**0.439**	**0.692**	**1**	**0.763**	**0.789**
**Mn/Ti (µ-XRF)**	143	0.37 – 3.34	0.88 ± 0.58	**0.236**	**0.395**	**0.374**	**0.435**	**0.347**	**0.452**	**0.412**	**0.327**	**0.466**	**0.582**	**1**	**0.831**
**Ca/Ti (µ-XRF)**	143	1.38 – 15.98	4.37 ± 2.64	**0.341**	**0.529**	**0.560**	**0.383**	**0.560**	**0.591**	**0.477**	**0.417**	**0.599**	**0.622**	**0.690**	**1**
				**Coefficients of determination (R^2):**											

(values in bold are different from 0 with a significance level α=0.05; whole core $F_{o.sed.}$ % correlation to CNS % is shown in (b)

**b)** % Mean $F_{o.sed.}$ vs CNS (1 cm sub-sample interval data)	n	min. –max.	Mean ± 1σ	**TC (mass%)**	**TN (mass%)**	**TS (mass%)**	**Mean $F_{o.sed.}$ (mass %)**	**TC (mass%)**	**TN (mass%)**	**TS (mass%)**	**Mean $F_{o.sed.}$ (mass %)**
				**Correlation matrix (Pearson r-values):**				***p*-values**			
**TC (mass%)**	137	0.0746 – 16.43	6.23 ± 4.04	**1**	**0.982**	0.133	**0.863**	**0**	**< 0.0001**	0.217	**< 0.0001**
**TN (mass%)**	132	0.0967 – 2.14	0.87 ± 0.49	**0.965**	**1**	**0.911**	**0.895**		**0**	**< 0.0001**	**< 0.0001**
**TS (mass%)**	88	0.1173 – 34.11	1.17 ± 3.59	0.018	**0.830**	**1**	-0.007			**0**	0.951
**Mean $F_{o.sed.}$ (mass %)**	133	-2.384 – 64.38	13.39 ± 14.67	**0.746**	**0.801**	0.000	**1**				**0**
				**Coefficients of determination (R^2):**							

(values in bold are different from 0 with a significance level α=0.05)

Supplementary Table 7 (below). Summary statistics for the five Guano Phases GP1–GP5 in the Ardley Lake sediment record compared with key Antarctic Peninsula terrestrial temperature datasets. Summary statistics for the equivalent time periods in the Yanou Lake GDGT-MSAT (mean summer (DJF) air temperature) record (this study) and the James Ross Island ice core temperature anomaly record[28] are shown. $F_{o.sed.}$ percentages >95 % upper mean bound on ARD record are shown in bold green. Temperatures and temperature anomalies >95% upper mean bound on the respective 6–0 ka record weighted mean value are shown in bold red. For YAN-GDGT, data outside of the >10 °C MSAT calibration upper limit was not included.

ARDLEY LAKE PENGUIN GUANO RECORD								ANTARCTIC PENINSULA TEMPERATURE RECORDS: YANOU LAKE			ANTARCTIC PENINSULA TEMPERATURE RECORDS: JAMES ROSS ISLAND		
ARDLEY CONISS Zone	**ARDLEY Guano Phase**	Core Depth min - max (cm)	Weighted Mean Age (cal a BP): Weighted Mean Min. – Max.	Weighted Mean Age (cal a BP): 95% Conf. Age Interval Min. – Max.	n	% Guano $F_{o.sed.}$ Weighted Mean (mass % ± 1σ)	% Guano $F_{o.sed.}$ 95% WM Min.–Max. (mass %)	n	GDGT Temp. MSAT W. mean (°C ± 1σ)	GDGT-MSAT Temp. 95% WM Min.-Max. (°C)	JRI Temp. Anom. 100-yr Means (°C ± uncertainty)	JRI Temp. Anom. 95% Mean Min.–Max. Temp. (°C)	
13		0 – 13	-60 – 1,290	-60 – 1,400	8	2.08 ± 2.87	-0.68 – 4.85	9	2.38 ± 2.13	0.74 – 4.01	-0.434 ± -0.092	-0.614 – -0.254	late Holocene
12	**5**	13 – 35	1,290 – 2,670	1,160 – 2,830	11	23.57 ± 10.74	16.35 – 30.79	4	**3.66 ± 2.54**	-0.39 **– 7.70**	-0.059 ± -0.012	-0.221 – 0.104	late Holocene
11		35 – 65	2,670 – 3,000	2,370 – 3,130	18	6.98 ± 2.86	3.98 – 9.98	2	**8.36 ± 0.35**	**5.18 – 11.54**	**0.188 ± 0.040**	**-0.121 – 0.498**	late Holocene
9	**4**	65 – 112	3,000 – 3,430	2,880 – 3,580	26	**43.66 ± 15.37**	**39.47 – 47.85**	4	**5.79 ± 2.00**	**2.62 – 8.97**	**0.262 ± 0.056**	**-0.156 – 0.680**	late Holocene
10		112 – 155	3,430 – 4,010	3,270 – 4,230	26	12.95 ± 2.76	11.41 – 14.49						late Holocene
8		155 – 175	4,010 – 4,310	3,810 – 4,500	10	-1.51 ± 2.54	-2.09 – -0.92	2	1.10 ± 0.21	-0.75 – 2.94	**0.346 ± 0.073**	**0.099 – 0.594**	mid Holocene
7	**3**	175 – 190	4,310 – 4,510	4,090 – 4,640	7	26.33 ± 12.16	18.92 – 33.74	1	0.70	-0.75 – 2.15	**0.323 ± 0.068**	**-0.344 – 0.990**	mid Holocene
6		190 – 284	4,510 – 5,300	4,310 – 5,450	58	5.65 ± 1.98	4.33 – 6.96	5	1.64 ± 1.92	-0.74 – 4.03	**0.247 ± 0.052**	**0.150 – 0.344**	mid Holocene
5	**2**	284 – 310	5,300 – 5,750	5,180 – 6,020	9	**29.38 ± 9.90**	**21.95 – 36.82**	5	-0.03 ± 0.57	-0.74 – 0.68	**0.107 ± 0.023**	**-0.140 – 0.355**	mid Holocene
4		310 – 320	5,750 – 6,270	5,530 – 6,920	5	-1.32 ± 2.53	-2.41 – -0.23	7	0.88 ± 1.32	-0.18 – 1.94	0.059 ± 0.012	-0.195 – 0.312	mid Holocene
**3**	**1**	320 – 326	6,270 – 6,670	5,820 – 7,360	3	26.77 ± 9.49	5.31 – 48.23	Marine-Glaciomarine Sediments			**0.140 ± 0.030**	**0.062 – 0.217**	mid Holocene
2		326 – 346	6,670 – 8,050	6,080 – 8,390	10	-2.21 ± 3.53	-2.47 – -1.95				**0.130 ± 0.027**	**0.042 – 0.217**	
1		346 – 358	8,050 – 8,750	7,550 – 9,230	7	-1.45 ± 1.68	-1.60 – -1.31				**0.209 ± 0.044**	**0.102 – 0.315**	
**Notes:** Temperature values in bold are greater than the 95% upper bound on 6–0 ka weighted mean or mean. GDGT-MSAT data >10 °C calibration limit were not included in statistical analysis.			**Summary**		198	25.31 ± 9.41	22.69 – **27.93**	39	2.39 ± 2.67	1.52 – 3.25	0.011 ± 0.002	-0.080 – **0.102**	
			**Guano Phases**		82	**34.19 ± 12.19**	**31.06 – 37.32**	14	**2.74 ± 2.98**	**1.02 – 4.46**	**0.102 ± 0.022**	**0.017 – 0.188**	
			**Non-Guano Phases**		116	3.56 ± 2.49	2.42 – 4.69	25	2.19 ± 2.51	1.15 – 3.22	0.021 ± 0.004	-0.073 – 0.114	
Guano % values in bold are are greater than the 95% upper bound on 8–0 ka weighted mean value.			**Late Holocene Guano Phases**		62	**36.57 ± 12.73**	**32.73 – 40.40**	8	**4.73 ± 2.40**	**2.71 – 6.74**	0.075 ± 0.016	-0.047 – 0.197	
			**Mid Holocene Guano Phases**		20	27.77 ± 10.72	23.66 – 31.88	6	0.09 ± 0.59	-0.53 – 0.71	**0.157 ± 0.033**	**0.061 – 0.013**	

Supplementary Table 8 (overpage). Summary statistics and two-population comparison statistics for guano versus non-guano hypothesis test scenarios. Scenarios A, B, C applied to reconstructed temperature datasets from Yanou Lake (YAN-GDGT), the James Ross Island (JRI) ice core and the Palmer Deep Sea Surface Temperature (PD-SST, 0–200 m) records. Scenarios D, E, F were applied to open water proxy datasets from the Maxwell Bay (%TOC) and shelf-edge Anvers Trough marine sediment records. See Methods & Results for set up conditions, definitions and references: In summary, A = Temperatures of 1) Guano phases were warmer than 2) Non-Guano phases for the Ardley Lake record timespan (to 9 ka); B = Temperatures of 1) Mid Holocene guano phases were warmer than 2) Late Holocene; C = Temperatures of Late Holocene 1) Guano phases were warmer than 2) Non-guano phases; D = More open water existed during 1) Guano phases than 2) Non-Guano phases Ardley Lake record timespan (to 9 ka); E = More open water existed during 1) Mid Holocene guano phases than during 2) Late Holocene guano phases; F = More open water existed during Late Holocene 1) Guano phases than during 2) Non-guano phases; *Null hypothesis: the ratio between the variances is equal to 1 (the variances are similar at 5% or 10% level); **Null hypothesis: The difference of location between the samples = 0 (no significant difference in temperature at 5% or 10% level). The % risk to reject the null hypotheses when it is in fact true = *p*-value expressed as a percentage. For YAN-GDGT scenario testing, data outside of the >10 °C calibration upper limit was not included.

Supplementary Table 8 (caption previous page).

	Temperature Scenarios	YAN-GDGT Temp. (^{o}C)			JRI Temp. Anomaly (^{o}C)			PD–SST(0–200 m)(^{o}C)		
		A	***B***	***C***	***A***	***B***	***C***	***A***	***B***	***C***
**1**	**No. observations**	14	6	8	36	12	24	28	12	16
	**Weighted Mean or Mean ± 1σ**	**2.74 ± 2.98**	**0.09 ± 0.59**	**4.73 ± 2.40**	**0.102 ± 0.022**	**0.157 ± 0.033**	**0.075 ± 0.016**	**0.48 ± 0.56**	**0.60 ± 0.50**	**0.39 ± 0.59**
	**95% mean bound range**	**1.02–4.46**	**-1.24**	**2.71–6.74**	**0.017–0.188**	**0.061–0.013**	**-0.047–0.197**	**0.26–0.69**	**0.23–0.91**	**0.08–0.71**
	**Median**	**1.57**	**-0.06**	**4.64**	**0.131**	**0.290**	**0.115**	**0.49**	**0.400**	**0.55**
	Normality Test (Shap.-Wilk.) *p*-value	0.09 (pass)	0.476 (pass)	0.865 (pass)	0.060 (pass)	0.805 (pass)	0.304 (pass)	0.874 (pass)	0.138 (pass)	0.172 (pass)
**2**	**No. of observations**	25	8	12	53	24	20	56	16	12
	**Weighted Mean or Mean ± 1σ**	**2.19 ± 2.51**	**4.73 ± 2.40**	**3.28 ± 3.01**	**0.021 ± 0.004**	**0.075 ± 0.016**	**-0.224 ± 0.425**	**0.97 ± 0.76**	**0.39 ± 0.59**	**0.84 ± 0.60**
	**95% mean bound range**	**1.15–3.22**	**2.71–6.74**	**1.37–5.19**	**-0.073–0.114**	**-0.047–0.197**	**-0.422– -0.025**	**0.76–1.17**	**0.08–0.71**	**0.46–1.22**
	**Median**	**1.49**	**4.64**	**1.87**	**0.119**	**0.115**	**-0.325**	**0.910**	**0.55**	**0.58**
	Normality Test (Shap.-Wilk.) *p*-value	<0.001 (fail)	0.865 (pass)	0.085 (pass)	<0.001 (fail)	0.304 (pass)	0.284 (pass)	<0.0001 (fail)	0.172 (pass)	0.135 (pass)
	Fisher's F-test Variance Ratio	1.41	0.06	0.64	0.552	0.27	0.47	0.535	0.71	0.97
	p-value (Two-tailed)*	0.451	0.0070	0.564	0.066	0.0283	0.082	0.079	0.5712	0.939
	Mann-Whitney (U)	182	0	65	1023	166	334	488	105	122
	p-value (Two-tailed)**	0.58	0.0003	0.91	0.57	0.48	0.03	0.01	0.69	0.25
	**Accept/reject null hypothesis**	**Accept**	**Reject 2<1**	**Accept**	**Accept**	**Accept**	**Reject 2>1**	**Reject 2<1**	**Accept**	**Accept**
	**Summary interpretation (signifcantly warmer tested at α = 0.05 (darker red shading) and 0.1 level. YAN GDGT-MSAT data >10 °C calibration limit were not included in statistical analysis**	**No significant difference in temperatures between guano and non-guano phases**	**Late Holocene guano phases were significantly warmer than mid Holocene guano phases**	**Late Holocene guano phases were not signifcantly warmer than late Holocene non-guano phases**	**No significant difference in temperatures between guano and non-guano phases**	**Late Holocene guano phases were not significantly warmer than mid Holocene guano phases**	**Late Holocene guano phases were significantly warmer than non-guano phases**	**Non-guano phases were significantly warmer than guano phases**	**Late Holocene guano phases were not significantly warmer than mid Holocene guano phases**	**Late Holocene guano phases were not signifcantly warmer than late Holocene non-guano phases**

	Open Water (OW) Scenarios	Maxwell Bay >OW (<%TOC)			Anvers Trough Pelagic OW Diatoms (>%)		
		D	***E***	***F***	***D***	***E***	***F***
**1**	**No. observations**	37	12	25	17	5	12
	**Weighted Mean or Mean ± 1σ**	**0.35 ± 0.05**	**0.33 ± 0.07**	**0.36 ± 0.03**	**19.21 ± 4.29**	**22.54 ± 5.52**	**17.82 ± 2.91**
	**95% mean bound range**	**0.34–0.37**	**0.28–0.37**	**0.35–0.38**	**17.00–21.41**	**15.69–29.40**	**15.97–19.67**
	**Median**	**0.37**	**0.37**	**0.37**	**18.03**	**23.54**	**17.75**
	Normality Test (Shap.-Wilk.) *p*-value	<0.001 (fail)	0.04 (pass 10%)	<0.001 (fail)	0.167 (pass)	0.614 (pass)	0.306 (pass)
**2**	**No. of observations**	54	25	17	26	12	10
	**Weighted Mean or Mean ± 1σ**	**0.32 ± 0.08**	**0.36 ± 0.03**	**0.38 ± 0.01**	**17.17 ± 7.91**	**17.82 ± 2.91**	**17.92 ± 3.90**
	**95% mean bound range**	**0.30–0.34**	**0.35–0.38**	**0.38–0.39**	**14.53–19.80**	**15.97–19.67**	**15.13–20.71**
	**Median**	**0.34**	**0.37**	**0.39**	**16.8**	**17.75**	**16.75**
	Normality Test (Shap.-Wilk.) *p*-value	<0.001 (fail)	<0.001 (fail)	0.002 (fail)	0.845 (pass)	0.306 (pass)	0.132 (pass)
	Fisher's F-test Variance Ratio	0.389	6.68	5.15	0.48	3.60	0.55
	p-value (Two-tailed)*	0.004	0.0001	0.001	0.14	0.08	0.35
	Mann-Whitney (U)	1140	111	129	174	46	62
	p-value (Two-tailed)**	0.26	0.22	0.03	0.25	0.10	0.92
	**Accept/reject null hypothesis**	**Accept**	**Accept**	**Reject 2<1**	**Accept**	**Reject 2>1**	**Accept**
	**Summary interpretation (signifcantly more open water at α = 0.05 (darker blue shading) and 0.1 level (lighter blue shading)**	**Open water conditions in Maxwell Bay during guano and non-guano phases were not significantly different**	**Open water conditions in Maxwell Bay during the mid and Late Holocene guano phases were not significantly different**	**Significantly more open water existed in Maxwell Bay in Late Holocene guano phases than in non-guano phases**	**Open water conditions at Anvers Shelf in guano and non-guano phases were not significantly different**	**More open water existed at Anvers Shelf in Mid Holocene guano phases GP1–2 than in Late Holocene guano phases GP3–5**	**Open water conditions at Anvers Shelf in Late Holocene guano and non-guano phases were not significantly different**

Supplementary Table 9. Input parameters for the penguin population modelling exercise. Terms used are as defined in Methods and Supplementary Note 8.

**1) Guano production - catchment erosion data**		**Value ±**	**Error**		**% err**
	Guano produced per penguin per day (g)[4]	84.5 ±	21.1	g	25
	Whole year occupation time (days)	365.25 ±	0.25	days	0.07
	Guano produced per penguin per year (whole year occupation)	30,864 ±	7,716	g a^{-1} penguin^{-1}	25
	Mean occupation time per year (days)[4]	91.3 ±	22.8	days	25
	Guano produced per penguin per year (mean year occupation)	7,716 ±	2,728	g a^{-1} penguin^{-1}	35
	Ardley Lake catchment area (5 m KGI-DEM)	6.62E+08 ±	6.62E+07	cm^2	10
	Ardley Lake area (5 m KGI-DEM)	7.27E+07 ±	7.27E+06	cm^2	10

**2) Guano-influenced sediment delivery (yield) scenarios**					
	100% guano-delivery scenario	7,716 ±	2,728	g a^{-1} penguin^{-1}	35
	11% guano-delivery scenario (lake: catchment area ratio[54])	847 ±	300	g a^{-1} penguin^{-1}	35
	0.96% guano-delivery scenario (Exponential lake area delivery function[54])	74 ±	26	g a^{-1} penguin^{-1}	35

**3) Lake area data for Fos DMAR influx calculations**					
A) MAX	1) ARD Lake MAX area	7.27E+07 ±	7.27E+06	cm^2	10
C) MIN	2) ARD Lake area >-3 m depth - stable deep platform	1.72E+07 ±	1.72E+06	cm^2	10
	3) ARD Lake area <45 degrees	7.07E+07 ±	7.07E+06	cm^2	10
B) MOST LIKELY	4) ARD Lake area <30 degrees (based on median slope of 27 degrees)	5.68E+07 ±	5.68E+06	cm^2	10
	5) ARD Lake area <15 degrees	3.74E+07 ±	3.74E+06	cm^2	10
	6) ACCUMLATION ZONE area calculation (after[56])	16.6 ±	12.0	mean slope (degrees)	72
	Accumulation Zone (%) (%ZA = 49.92(±3.37) - 2.50(±0.31) * (mean slope))	8.5 ±	6.2	%	73
	ZA Accumulation Zone area for Ardley Lake	6.18E+06 ±	3.86E+05	cm^2	6

**4) Basin modelling input parameters[57]**		**Water**	**Sed. Core**	**z mean**	**z max (cm)**
	Mean water depth (cm)	126	178	303	
	Standard deviation (n-1)	78	251	263	
	Upper 1σ	48	74	40	
	Lower 1σ	78	251	263	
	z_m (max)		570	355	925
	z(mean):z(max)	0.327654046			
	z_a				974

**5) Penguin densities (for area occupied calculations; based on[42])**	**Density ±**	**Error**		**% err**
Gentoo	0.31 ±	0.19	per m^2	61
Adelie	1.48 ±	1.08	per m^2	73
Chinstraps	0.53 ±	0.33	per m^2	62
ARD Partitioned Density	0.57 ±	0.53	per m^2	93
(according to[4]: Ardley population distribution: 74% Gentoo; 21% Adelie; 5% Chinstraps)				

Supplementary Table 10. Glass shard major element and selected trace element electron microprobe analysis data for the most prominent T2–T9 tephra layers in the YAN, ARD, Beak1 and PC460/1 records. (a) Mean and 1σ error summary geochemistry for glass shards with a Trachydacitic-rhyolitic composition. **(b)** Mean and 1σ error summary geochemistry for glass shards with a basic-intermediate composition. For comparison with YAN and ARD tephra deposits we include data from the Beak 1 Lake record (Beak Island; Layers Ta–Te[34]), T7 age-equivalent tephra layers in marine cores PC460/461 (this study) and EPMA data from the Bransfield (Boyd) Strait, Scotia Sea and northern Weddell Sea[59] (see Supplementary Fig. 21). Units for element oxides and $H_2O_{diff.}$ are mass %; * indicates additional elements not always included.See Supplementary Data 7 for all data.

a) Group 1: Trachydacitic–rhyolitic

Eruption	Core & Depth	Strat. depth (cm)	Mean Age (cal a BP)	n	SiO_2	TiO_2	Al_2O_3	FeO_{tot}	MnO	MgO	CaO	Na_2O	K_2O	P_2O_5	SO_2*	F (mg kg^{-1})*	Cl (mg kg^{-1})*	TOTAL	$H_2O_{diff.}$
**T7b**	PC461_63-66.5cm	350–353.5	7,020	1	63.97	1.24	15.66	5.54	0.19	1.46	3.41	6.22	1.43	0.27	0.10	890	1549	99.72	0.28
**T7b**	YAN8B-1 41-41.5	259–259.5	7,180	9	70.23	0.60	14.35	3.74	0.17	0.51	1.26	6.71	2.19	0.10	0.03	1,123	2,226	100.22	0.22
				± 1σ	2.56	0.24	0.60	0.95	0.02	0.28	0.57	0.32	0.26	0.07	0.01	224	282	0.80	0.45
**T7a**	PC460 73.5-74	598.5–599	7,990	2	67.96	0.55	13.74	4.10	0.17	0.43	1.36	4.63	3.21	0.08	0.01	621	2,032	96.51	3.49
				± 1σ	5.52	0.22	0.01	1.48	0.03	0.27	0.87	3.04	1.14	0.06	0.00	642	604	4.64	4.64
**T9**	Beak1D_70.5-88	135.5–136	10,560	7	71.13	0.44	14.05	3.56	0.16	0.29	0.92	6.48	2.36	0.07				99.46	0.63
				± 1σ	1.68	0.14	058	0.76	0.03	0.19	0.53	0.17	0.30	0.05				0.99	0.91
**T7–T9: Summary**				**18/11***	**70.33**	**0.53**	**14.16**	**3.71**	**0.16**	**0.41**	**1.14**	**6.39**	**2.37**	**0.08**	**0.03**	**1,032**	**2,191**	**99.51**	**0.74**
				**± 1σ**	**2.61**	**0.21**	**0.58**	**0.88**	**0.02**	**0.25**	**0.57**	**1.01**	**0.49**	**0.06**	**0.01**	**350**	**326**	**1.80**	**1.64**
**Bransfield (Boyd) Strait, Scotia Sea & northern Weddell Sea[58]**				**58**	**70.14**	**0.48**	**14.39**	**3.40**	**0.14**	**0.31**	**0.96**	**5.55**	**2.27**					**97.64**	**2.36**
				**± 1σ**	**1.90**	**0.16**	**0.78**	**0.35**	**0.04**	**0.23**	**0.53**	**0.62**	**0.33**					**1.09**	**1.09**

b) Group 2: Basic–Intermediate

Eruption	Core & Depth	Strat. depth (cm)	Mean Age (cal a BP)	n	SiO_2	TiO_2	Al_2O_3	FeO_{tot}	MnO	MgO	CaO	Na_2O	K_2O	P_2O_5	SO_2*	F (mg kg^{-1})*	Cl (mg kg^{-1})*	TOTAL	$H_2O_{diff.}$
**T2**	Beak1E_32-33cm	32.5–33	2,330	24	53.85	2.26	14.35	10.02	0.20	4.35	8.16	4.60	0.75	0.33				98.87	1.17
				± 1σ	1.94	0.37	0.89	1.15	0.02	0.75	1.19	0.61	0.33	0.05				0.85	0.79
**T4**	ARD1C 53-54 cm	163–164	4,150	25	53.73	2.24	14.82	10.00	0.19	4.29	7.85	4.71	0.73	0.31	0.04	672	826	99.05	1.04
				± 1σ	1.68	0.56	1.18	1.65	0.03	0.66	0.97	0.35	0.15	0.09	0.03	142	269	0.99	0.83
**T5b**	YAN8A-1: 58 cm	58–59	4,800	7	53.97	1.73	16.17	8.27	0.16	4.13	7.93	4.77	0.65	0.25	0.06	644	789	98.23	1.77
				± 1σ	1.12	0.32	0.45	0.78	0.02	0.16	0.29	0.11	0.07	0.06	0.02	77	130	0.54	0.54
**T5b**	Beak1E_58.5-5	58.5–59	4,860	25	53.30	2.48	14.97	10.38	0.19	4.50	7.74	4.53	0.76	0.37				99.20	0.80
				± 1σ	2.39	0.36	1.11	0.99	0.02	1.43	1.20	0.42	0.19	0.07				0.51	0.51
**T6**	YAN8A-2: 82 cm	182.5–183.5	5,630	7	54.17	1.59	16.20	8.06	0.16	4.77	8.39	4.58	0.60	0.22	0.04	522	762	98.91	1.11
				± 1σ	2.45	0.09	0.60	0.70	0.01	1.49	1.55	0.49	0.22	0.03	0.02	128	337	1.12	1.09
**T7c**	YAN 9B-1 36.5-37	226–226.5	6,450	25	54.38	2.17	14.87	9.53	0.19	4.00	7.51	4.63	0.76	0.32	0.03	763	812	98.54	1.50
				± 1σ	1.67	0.55	1.14	1.59	0.03	0.64	0.89	0.78	0.16	0.11	0.02	165	225	0.82	0.72
**T7b**	PC461_63-66.5cm	350–353.5	7,020	10	51.50	2.27	15.48	9.95	0.19	4.68	8.79	4.49	0.51	0.33	0.08	570	513	98.38	1.62
				± 1σ	0.52	0.27	0.55	0.64	0.02	0.79	0.96	0.31	0.08	0.05	0.03	197	132	0.69	0.69
**T7b**	YAN8B-1 41-41.5	259–259.5	7,180	17	52.21	2.34	15.74	10.02	0.19	4.24	8.25	4.83	0.55	0.36	0.11	622	575	98.97	1.10
				± 1σ	1.48	0.22	0.26	0.79	0.01	0.71	0.92	0.45	0.11	0.04	0.06	133	96	0.95	0.85
**T7a**	PC460 73.5-74	598.5–599	7,990	23	51.80	2.33	15.64	10.29	0.19	4.64	8.84	4.34	0.51	0.36	0.07	601	500	99.12	0.92
				± 1σ	0.97	0.23	0.50	0.92	0.02	0.63	0.79	0.53	0.09	0.05	0.03	218	87	0.66	0.56
**T9**	Beak1D_70.5-89	135.5–136	10,560	18	52.33	2.52	15.64	10.19	0.20	3.88	7.73	4.71	0.59	0.43				98.21	1.79
				± 1σ	2.02	0.34	0.76	0.96	0.02	0.42	0.80	0.75	0.15	0.07				0.91	0.91
**T2–T9: Summary**				**183/115***	**53.21**	**2.26**	**15.20**	**9.88**	**0.19**	**4.30**	**8.03**	**4.62**	**0.67**	**0.34**	**0.06**	**652**	**693**	**98.83**	**1.21**
				**± 1σ**	**2.12**	**0.44**	**1.02**	**1.31**	**0.02**	**0.88**	**1.12**	**0.57**	**0.21**	**0.08**	**0.04**	**177**	**252**	**0.86**	**0.80**
**Bransfield (Boyd) Strait, Scotia Sea & northern Weddell Sea[59]**				191	52.14	2.18	15.70	9.61	0.18	4.12	8.13	4.54	0.60					97.20	2.85
				± 1σ	1.80	0.42	0.98	1.38	0.04	0.75	0.99	0.43	0.17					1.37	1.22

Supplementary References

1. Liu, X. D., Nie, Y., Sun, L. & Emslie, S. D. Eco-environmental implications of elemental and carbon isotope distributions in ornithogenic sediments from the Ross Sea region, Antarctica. *Geochim. Cosmochim. Acta* **117,** 99–114 (2013).
2. Huang, T., Sun, L., Wang, Y. & Kong, D. Late Holocene Adélie penguin population dynamics at Zolotov Island, Vestfold Hills, Antarctica. *J. Paleolimnol.* **45,** 273–285 (2011).
3. Sun, L., Xie, Z. & Zhao, J. A 3,000-year record of penguin populations. *Nature* **407,** 858 (2000).
4. Liu, X. D., Sun, L. G., Li, D. & Wang, Y. H. Rare earth elements in the ornithogenic sediments from the Maritime Antarctic: A potential new palaeoecology proxy. *Geochem. J.* **45,** 15–26 (2011).
5. Zale, R. Changes in size of the Hope Bay Adélie penguin rookery as inferred from Lake Boeckella sediment. *Ecography* **17,** 297–304 (1994).
6. Hofstee, E. H., Balks, M. R., Petchey, F. & Campbell, D. I. Soils of Seabee Hook, Cape Hallett, northern Victoria Land, Antarctica. *Antarct. Sci.* **18,** 473–486 (2006).
7. Huang, T., Sun, L., Wang, Y., Liu, X. & Zhu, R. Penguin population dynamics for the past 8500 years at Gardner Island, Vestfold Hills. *Antarct. Sci.* **21,** 571–578 (2009).
8. Liu, X. *et al.* A preliminary record of the historical seabird population in the Larsemann Hills, East Antarctica, from geochemical analyses of Mochou Lake sediments. *Boreas* **36,** 182–197 (2007).
9. Dearing, J. in *Handbook Of Holocene Palaeoecology and Palaeohydrology* (ed. Berglund, B.) 247–270 (Wiley, 1986).
10. Tatur, A. & Barczuk, A. in *Antarctic Nutrient Cycles and Food Webs* (eds. Siegfried, W. R., Condy, P. R. & Laws, R. M.) 163–168 (Springer, 1985).
11. Sauer, G., Wu, L., Iijima, M. & Wuthier, R. The influence of trace elements on calcium phosphate formation by matrix vesicles. *J. Inorg. Biochem.* **65,** 57–65 (1997).
12. Abraitis, P. K., Pattrick, R. A. D. & Vaughan, D. J. Variations in the compositional, textural and electrical properties of natural pyrite: a review. *Int. J. Miner. Process.* **74,** 41–59 (2004).
13. Tribovillard, N., Algeo, T. J., Lyons, T. & Riboulleau, A. Trace metals as paleoredox and paleoproductivity proxies: An update. *Chem. Geol.* **232,** 12–32 (2006).
14. Jarvis, I. *et al.* Phosphorite geochemistry: state-of-the-art and environmental concerns. *Eclogae Geol. Helv.* **87,** 643–700 (1994).
15. Watcham, E. P. *et al.* A new Holocene relative sea level curve for the South Shetland Islands, Antarctica. *Quat. Sci. Rev.* **30,** 3152–3170 (2011).
16. Sun, L. *et al.* A geochemical method for the reconstruction of the occupation history of a penguin colony in the maritime Antarctic. *Polar Biol.* **27,** 670–678 (2004).
17. Liu, X. D., Sun, L. G., Xie, Z. Q., Yin, X. B. & Wang, Y. H. A 1300-year record of penguin populations at Ardley Island in the Antarctic, as deduced from the geochemical data in the ornithogenic lake sediments. *Arct. Antarct. Alp. Res.* **37,** 490–498 (2005).
18. Liu, X. *et al.* A preliminary study of elemental geochemistry and its potential application in Antarctic Seal palaeoecology. *Geochem. J.* **39,** 47–59 (2005).
19. Emslie, S. D., Polito, M. J., Brasso, R., Patterson, W. P. & Sun, L. Ornithogenic soils and the paleoecology of pygoscelid penguins in Antarctica. *Quat. Int.* **352,** 4–15 (2014).
20. Royles, J. *et al.* Carbon isotope evidence for recent climate-related enhancement of CO2 assimilation and peat accumulation rates in Antarctica. *Glob. Chang. Biol.* **18,** 3112–3124 (2012).

21. Emslie, S. D., Polito, M. J. & Patterson, W. P. Stable isotope analysis of ancient and modern gentoo penguin egg membrane and the krill surplus hypothesis in Antarctica. *Antarct. Sci.* **25,** 213–218 (2013).
22. Foster, L. C. *et al.* Development of a regional glycerol dialkyl glycerol tetraether (GDGT)-temperature calibration for Antarctic and sub-Antarctic lakes. *Earth Planet. Sci. Lett.* **433,** (2016).
23. Simms, A. R., Milliken, K. T., Anderson, J. B. & Wellner, J. S. The marine record of deglaciation of the South Shetland Islands, Antarctica since the Last Glacial Maximum. *Quat. Sci. Rev.* **30,** 1583–1601 (2011).
24. Shevenell, A., Ingalls, A., Domack, E. W. & Kelly, C. Holocene Southern Ocean surface temperature variability west of the Antarctic Peninsula. *Nature* **470,** 250–254 (2011).
25. Barbara, L. *et al.* Environmental responses of the Northeast Antarctic Peninsula to the Holocene climate variability. *Paleoceanography* **31,** 131–147 (2016).
26. Etourneau, J. *et al.* Holocene climate variations in the western Antarctic Peninsula: evidence for sea ice extent predominantly controlled by changes in insolation and ENSO variability. *Clim. Past* **9,** 1431–1446 (2013).
27. Milliken, K. T., Anderson, J. B., Wellner, J. S., Bohaty, S. M. & Manley, P. L. High-resolution Holocene climate record from Maxwell Bay, South Shetland Islands, Antarctica. *Geol. Soc. Am. Bull.* **121,** 1711–1725 (2009).
28. Mulvaney, R. *et al.* Recent Antarctic Peninsula warming relative to Holocene climate and ice-shelf history. *Nature* **489,** 141–144 (2012).
29. Hughes, K. A., Ireland, L. C., Convey, P. & Fleming, A. H. Assessing the effectiveness of specially protected areas for conservation of Antarctica's botanical diversity. *Conserv. Biol.* **30,** 113–120 (2016).
30. Casanovas, P., Lynch, H. J. & Fagan, W. F. Multi-scale patterns of moss and lichen richness on the Antarctic Peninsula. *Ecography* **36,** 209–219 (2013).
31. Zhu, R. *et al.* Penguins significantly increased phosphine formation and phosphorus contribution in maritime Antarctic soils. *Sci. Rep.* **4,** 7055 (2014).
32. Wright, H. A Square-Rod Piston Sampler for Lake Sediments. *J. Sediment. Petrol.* **37,** 975–& (1967).
33. Ó Cofaigh, C. *et al.* Reconstruction of ice-sheet changes in the Antarctic Peninsula since the Last Glacial Maximum. *Quat. Sci. Rev.* **100,** 87–110 (2014).
34. Roberts, S. J. *et al.* Geological constraints on glacio-isostatic adjustment models of relative sea-level change during deglaciation of Prince Gustav Channel, Antarctic Peninsula. *Quat. Sci. Rev.* **30,** 3603–3617 (2011).
35. Sterken, M. *et al.* Holocene glacial and climate history of Prince Gustav Channel, northeastern Antarctic Peninsula. *Quat. Sci. Rev.* **31,** 93–111 (2012).
36. Emslie, S. D., Fraser, W., Smith, R. C. & Walker, W. Abandoned penguin colonies and environmental change in the Palmer Station area, Anvers Island, Antarctic Peninsula. *Antarct. Sci.* **70,** 257–268 (1998).
37. Emslie, S. D., Baumann, K. & van Tuinen, M. Late Holocene occupation of Gentoo penguins (Pygoscelis papua) on Byers Peninsula, Livingston Island, Antarctica. *Polar Biol.* **34,** 283–290 (2011).
38. Del Valle, R. A., Montalti, D. & Inbar, M. Mid-Holocene macrofossil-bearing raised marine beaches at Potter Peninsula, King George Island, South Shetland Islands. *Antarct. Sci.* **14,** 263–269 (2002).
39. Reimer, P. J. *et al.* INTCAL09 and MARINE09 radiocarbon age calibration curves, 0-50,000 years cal BP. *Radiocarbon* **51,** 1111–1150 (2009).
40. Emslie, S. D. Radiocarbon dates from abandoned penguin colonies in the Antarctic Peninsula region. *Antarct. Sci.* **13,** 289–295 (2001).
41. Pudsey, C. J., Barker, P. F. & Larter, R. D. Ice sheet retreat from the Antarctic

Peninsula shelf. *Cont. Shelf Res.* **14,** 1647–1675 (1994).
42. Waluda, C. M., Dunn, M. J., Curtis, M. L. & Fretwell, P. T. Assessing penguin colony size and distribution using digital mapping and satellite remote sensing. *Polar Biol.* **37,** 1849–1855 (2014).
43. Björck, S., Hjort, C., Ingolfsson, O. & Skog, G. in *Radiocarbon Dating: Recent Applications and Future Potential* (ed. Lowe, J.) 55–56 (Quaternary Proceedings. Quaternary Research Association, 1991).
44. Berkman, P. A. & Foreman, S. Pre-bomb radiocarbon and the reservoir correction for calcareous marine species in the Southern Ocean. *Geophys. Res. Lett.* **23,** 363–366 (1996).
45. Hall, B. L. Holocene relative sea-level changes and ice fluctuations in the South Shetland Islands. *Glob. Planet. Change* **74,** 15–26 (2010).
46. Roberts, S. J. *et al.* The Holocene history of George VI ice shelf, Antarctic Peninsula from clast-provenance analysis of epishelf lake sediments. *Palaeogeogr. Palaeoclimatol. Palaeoecol.* **259,** (2008).
47. Toro, M. *et al.* Chronostratigraphy of the sedimentary record of Limnopolar Lake, Byers Peninsula, Livingston Island, Antarctica. *Antarct. Sci.* **25,** 198–212 (2013).
48. Björck, S., Hjort, C., Ingolfsson, O. & Skog, G. in *Radiocarbon Dating: Recent Applications and Future Potential. Quaternary Proceedings No. 1* (ed. Lowe, J.) 55–65 (Quaternary Research Association, 1991).
49. Polito, M. J., Lynch, H. J., Naveen, R. & Emslie, S. D. Stable isotopes reveal regional heterogeneity in the pre-breeding distribution and diets of sympatrically breeding Pygoscelis spp. penguins. *Mar. Ecol. Prog. Ser.* **421,** 265–277 (2011).
50. Cherel, Y. & Hobson, K. A. Geographical variation in carbon stable isotope signatures of marine predators: a tool to investigate their foraging areas in the Southern Ocean. *Mar. Ecol. Prog. Ser.* **329,** 281–287 (2007).
51. Willmott, V., Domack, E. W., Canals, M. & Brachfeld, S. A high resolution relative paleointensity record from the Gerlache-Boyd paleo-ice stream region, northern Antarctic Peninsula. *Quat. Res.* **66,** 1–11 (2006).
52. Björck, S., Håkansson, H., Zale, R., Karlen, W. & Jónsson, B. L. A late Holocene lake sediment sequence from Livingston Island, South Shetland Islands, with palaeoclimatic implications. *Antarct. Sci.* **3,** 61–72 (1991).
53. Braun, M. *et al.* An improved topographic database for King George Island: compilation, application and outlook. *Antarct. Sci.* **13,** (2001).
54. Dearing, J. A. & Foster, I. in *Handbook Of Holocene Palaeoecology and Palaeohydrology* (ed. Berglund, B.) 67–89 (Wiley, 1986).
55. Zolitschka, B. A 14,000 year sediment yield record from western Germany based on annually laminated lake sediments. *Geomorphology* **22,** 1–17 (1998).
56. Blais, J. M. & Kalff, J. The influence of lake morphometry on sediment focusing. *Limnol. Oceanogr.* **40,** 582–588 (1995).
57. Lehman, J. Reconstructing the rate of accumulation of lake sediment: the effect of sediment focussing. *Quat. Res.* **5,** 541–550 (1975).
58. Fretzdorff, S. & Smellie, J. L. Electron microprobe characterization of ash layers in sediments from the central Bransfield basin (Antarctic Peninsula): evidence for at least two volcanic sources. *Antarct. Sci.* **14,** 412–421 (2003).
59. Moreton, S. G. & Smellie, J. L. Identification and correlation of distal tephra layers in deep-sea sediment cores, Scotia Sea, Antarctica. *Ann. Glaciol.* **27,** 285–289 (1998).
60. Bartolini, S., Geyer, A., Martí, J., Pedrazzi, D. & Aguirre-Díaz, G. Volcanic hazard on Deception Island (South Shetland Islands, Antarctica). *J. Volcanol. Geotherm. Res.* **285,** 150–168 (2014).
61. Pedrazzi, D., Aguirre-Díaz, G., Bartolini, S., Martí, J. & Geyer, A. The 1970 eruption on Deception Island (Antarctica): eruptive dynamics and implications for volcanic

hazards. *J. Geol. Soc. London.* **171,** 765–778 (2014).
62. Davies, S., Lamb, H. & Roberts, S. in *Micro-XRF Studies of Sediment Cores: Applications of a non-destructive tool for the environmental sciences* (eds. Croudace, I. W. & Rothwell, R. G.) 189–226 (Springer, 2015).
63. Mayer, L., Schick, L., Allison, M., Ruttenberg, K. & Bentley, S. Marine vs. terrigenous organic matter in Louisiana coastal sediments: The uses of bromine : organic carbon ratios. *Mar. Chem.* **107,** 244–254 (2007).
64. Cota, G. F. & Sturges, W. T. Biogenic bromine production in the Arctic. *Mar. Chem.* **56,** 181–192 (1997).
65. Kerfoot, W. C., Robbins, J. A. & Weider, L. J. A new approach to historical reconstruction: Combining descriptive and experimental paleolimnology. *Limnol. Oceanogr.* **44,** 1232–1247 (1999).
66. Turner, J. *et al.* Absence of 21st century warming on Antarctic Peninsula consistent with natural variability. *Nature* **535,** 411–415 (2016).
67. Heroy, D. C., Sjunneskog, C. & Anderson, J. B. Holocene climate change in the Bransfield Basin, Antarctic Peninsula: evidence from sediment and diatom analysis. *Antarct. Sci.* **20,** 69–87 (2008).
68. Bentley, M. J. *et al.* Mechanisms of Holocene palaeoenvironmental change in the Antarctic Peninsula region. *The Holocene* **19,** 51–69 (2009).
69. Martí, J., Geyer, A. & Aguirre-Diaz, G. Origin and evolution of the Deception Island caldera (South Shetland Islands, Antarctica). *Bull. Volcanol.* **75,** 732 (2013).
70. Smellie, J. L. The upper Cenozoic tephra record in the south polar region: a review. *Glob. Planet. Change* **21,** 51–70 (1999).
71. Smellie, J. L. Lithostratigraphy and volcanic evolution of Deception Island, South Shetland Islands. *Antarct. Sci.* **13,** 188–209 (2001).
72. Brown, S., MR, A. & Sparks, R. in *Global Volcanic Hazards and Risk* (eds. Loughlin, S. C., Sparks, S., Brown, S. K., Jenkins, S. F. & Vye-Brown, C.) 223–232 (Cambridge University Press, 2015).
73. Hobbs, L. K., Gilbert, J. S., Lane, S. J. & Loughlin, S. in *Global Volcanic Hazards and Risk* (eds. Loughlin, S. C., Sparks, S., Brown, S. K., Jenkins, S. F. & Vye-Brown, C.) 77–78 (Cambridge University Press, 2015).
74. Jenkins, S. *et al.* in *Global Volcanic Hazards and Risk* (eds. Loughlin, S., Sparks, S., Brown, S., Jenkins, S. & Vye-Brown, C.) 173–222 (Cambridge University Press, 2015).
75. Van Daele, M. *et al.* The 600 yr eruptive history of Villarrica Volcano (Chile) revealed by annually laminated lake sediments. *Geol. Soc. Am. Bull.* **126,** 481–498 (2014).
76. Smellie, J. L., Hofstetter, A. & Troll, G. Fluorine and boron geochemistry of an ensialic marginal basin volcano: Deception Island, Bransfield Strait, Antarctica. *J. Volcanol. Geotherm. Res.* **49,** 255–267 (1992).
77. Sparks, R. S. J. *Volcanic Plumes*. (Wiley, 1997).
78. Blong, R. *Volcanic hazards: a sourcebook on the effects of eruptions*. (Academic Press, 1984).
79. Yin, X. *et al.* Why do penguins not develop skeletal fluorosis? *Fluoride* **43,** 108–118 (2010).
80. Ancora, S., Volpi, V., Olmastroni, S., Focardi, S. & Leonzio, C. Assumption and elimination of trace elements in Adélie penguins from Antarctica: a preliminary study. *Mar. Environ. Res.* **54,** 341–344 (2002).
81. Rainbow, P. Copper, cadmium and zinc concentrations in oceanic amphipod and euphausiid crustaceans, as a source of heavy-metals to pelagic seabirds. *Mar. Biol.* **103,** 513–518 (1989).
82. Mercone, D., Thomson, J., Croudace, I. W. & Troelstra, S. R. A coupled natural immobilisation mechanism for mercury and selenium in deep-sea sediments.

Geochim. Cosmochim. Acta **63,** 1481–1488 (1999).
83. Magos, L. & Webb, M. The Interactions of Selenium with Cadmium and Mercury. *Crc Crit. Rev. Toxicol.* **8,** 1–42 (1980).
84. Tatur, A. Fluorine in ornithogenic soils and minerals on King George Island,West Antarctica. *Polish Polar Res.* **8,** 65–74 (1987).
85. Metcheva, R. *et al.* Trace and toxic elements accumulation in food chain representatives at Livingston Island (Antarctica). *Int. J. Biol.* **2,** 155–161 (2010).
86. Ratnarajah, L., Bowie, A. R., Lannuzel, D., Meiners, K. M. & Nicol, S. The biogeochemical role of baleen whales and krill in Southern Ocean nutrient cycling. *PLoS One* **9,** e114067 (2014).
87. Schmidt, K. *et al.* Zooplankton Gut Passage Mobilizes Lithogenic Iron for Ocean Productivity. *Curr. Biol.* **26,** 2667–2673 (2016).
88. Sands, M., Nicol, S. & McMinn, A. Fluoride in Antarctic marine crustaceans. *Mar. Biol.* **132,** 591–598 (1998).
89. Soszka, G. J., Suplinska, M. M., Baranski, A., Grzybowska, D. & Pietruszewski, A. Trace metals, fluorine and radionuclides in antarctic krill Euphausia superba Dana. *Polish Polar Res.* **2,** 109–117 (1981).
90. Culik, B. Fluoride turnover in Adélie penguins (Pygoscelis adeliae) and other brid species. *Polar Biol.* **7,** 179–187 (1987).
91. Gunn, D. & Best, A. A new automated non- destructive system for high resolution multi-sensor core logging of open sediment cores. *Geo-Marine Lett.* **18,** 70–77 (1998).
92. Kylander, M. E., Ampel, L., Wohlfahrt, B. & Veres, D. High-resolution X-ray fluorescence core scanning analysis of Les Echets (France) sedimentary sequence: new insights from chemical proxies. *J. Quat. Sci.* **26,** 109–117 (2011).
93. Bertrand, Sebastien, Hughen, K. & Giosan, L. in *Micro-XRF Studies of Sediment Cores: Applications of a non-destructive tool for the environmental sciences* (eds. Croudace, I. W. & Rothwell, R.) 473–490 (2015).
94. Blott, S. & Pye, K. Gradistat: a grain size distribution and statistics package for the analysis of unconsolidated sediments. *Earth Surf. Process. Landforms* **26,** 1237–1248 (2001).
95. Dean, W. Determination of carbonate and organic matter in calcareous sediments and sedimentary rocks by loss on ignition, comparison with other models. *J. Sediment. Petrol.* **44,** 242–248 (1974).
96. Zolitschka, B. in *Relief Boden Paläoklima 13* 176 (Gebrüder Bornträger Verlag, 1998).
97. Street-Perrot, F. *et al.* Late Quaternary changes in ecosystems and carbon cycling on Mt. Kenya, East Africa: a landscape-ecological perspective based on multi-proxy lake-sediment influxes. *Quat. Sci. Rev.* **26,** 1838–1860 (2007).
98. Hedges, R., Law, I., Bronk, C. & Housley, R. The Oxford Accelerator Mass Spectrometry Facility: technical developments in routine dating. *Archaeometry* **31,** 99–113 (1989).
99. Bronk Ramsey, C. Bayesian analysis of radiocarbon dates. *Radiocarbon* **51,** 337–360 (2009).
100. McCormac, F. G. *et al.* SHCal04 southern hemisphere calibration, 0-11.0 cal kyr BP. *Radiocarbon* **46,** 1087–1092 (2004).
101. Reimer, P. J. *et al.* INTCAL04 terrestrial radiocarbon age calibration, 0–26 cal kyr BP. *Radiocarbon* **46,** 1029–1058 (2004).
102. Hua, Q., Barbetti, M. & Rakowski, A. Z. Atmospheric radiocarbon for the period 1950-2010. *Radiocarbon* **55,** 1–14 (2013).
103. Reimer, R. W. & Reimer, P. J. CALIBomb - calibration of post-bomb C-14 data. (2004). at <www.calib.org>

104. Blaauw, M. & Christen, J. Flexible palaeoclimate age-depth models using an autoregressive gamma process. *Bayesian Anal.* **6,** 457–474 (2011).
105. Blaauw, M. Methods and code for 'classical' age-modelling of radiocarbon sequences. *Quat. Geochronol.* **5,** 512–518 (2010).
106. Hogg, A. *et al.* SHCAL13 Southern Hemisphere calibration, 0-50,000 years cal BP. *Radiocarbon* **55,** 1889–1903 (2013).
107. Reimer, P. J. *et al.* INTCAL13 and MARINE13 radiocarbon age calibration curves 0-50,000 years cal BP. *Radiocarbon* **55,** 1869–1887 (2013).
108. Heinrichs, H., Brumsack, H.-J., Loftfield, N. & König, N. Verbessertes Druckaufschlußsystem für biologische und anorganische Materialien. *Z Pflanz Bodenkd.* **149,** 350–353 (1986).
109. Skoog, D. A. & Leary, J. J. *Principles of Instrumental Analysis*. (1992).
110. Scherer, R. P. A new method for the determination of absolute abundance of diatoms and other silt-sized sedimentary particles. *J. Paleolimnol.* **12,** 171–179 (1994).
111. Tomas, C. *Identifying Marine Phytoplankton*. (Academic Press Inc., 1997).
112. Scott, F. J. & Marchant, H. J. *Antarctic Marine Protists*. (Australian Biological Resources Study (ABRS). CSIRO Publishing, 2005).
113. Buffen, A., Leventer, A., Rubin, A. & Hutchins, T. Diatom assemblages in surface sediments of the northwestern Weddell Sea, Antarctic Peninsula. *Mar. Micropaleontol.* **62,** 7–30 (2007).
114. Massé, G. *et al.* Highly branched isoprenoids as proxies for variable sea ice conditions in the Southern Ocean. *Antarct. Sci.* **23,** 487–498 (2011).
115. Kim, J.-H. *et al.* Holocene subsurface temperature variability in the eastern Antarctic continental margin. *Geophys. Res. Lett.* **39,** L06705 (2012).
116. Peck, V. L., Allen, C. S., Kender, S., McClymont, E. L. & Hodgson, D. A. Oceanographic variability on the West Antarctic Peninsula during the Holocene and the influence of upper circumpolar deep water. *Quat. Sci. Rev.* **119,** 54–65 (2015).
117. Hayward, C. High spatial resolution electron probe microanalysis of tephras and melt inclusions without beam-induced chemical modification. *The Holocene* **22,** 119–125 (2012).
118. Hunt, J. B. & Hill, P. G. Tephra geochemistry: a discussion of some persistent analytical problems. *The Holocene* **3,** 271–278 (1993).
119. Sweatman, T. & Long, J. Quantitative electron-probe micro- analysis of rock-forming minerals. *J. Petrol.* **10,** 332–379 (1969).
120. Westgate, J. & Gorton, M. in *Tephra Studies* (eds. Self, S. & Sparks, R.) 73–94 (Reidel, 1981).
121. Suzuki, R. & Shimodaira, H. Pvclust: an R package for assessing the uncertainty in hierarchical clustering. *Bioinformatics* **22,** 1540–2 (2006).
122. Oksanen, J. *et al.* Vegan: Community Ecology Package. R package version 2.0-7. (2013).
123. Oksanen, J. *et al.* Vegan: Community Ecology Package. R package version 2.3-0. (2014). at <http://cran.r-project.org/web/packages/vegan/index.html>
124. Juggins, S. *C2 Version 1.5 User Guide. Software for ecological and palaeoecological data analysis and visualisation*. (Newcastle University, 2007).
125. Juggins, S. Rioja: Analysis of Quaternary Science Data, R package version (0.9-3). (2014).
126. Juggins, S. Rioja: Analysis of Quaternary Science Data, R package version (0.8-5). (2013).
127. Paillard, D., Labeyrie, L. & Yiou, P. Macintosh program performs time-series analysis. *Eos (Washington. DC).* **77:379,** (1996).
128. Boyle, J., Chiverrell, R. & Schillereff, D. in *Micro-XRF Studies of Sediment Cores:*

Applications of a non-destructive tool for the environmental sciences (eds. Croudace, I. & Rothwell, R.) 373–390 (Springer, 2015).
129. Gallagher, J. in *Antarctic Nutrient Cycles and Food Webs* (eds. Siegfried, W. R., Condy, P. R. & Laws, R. M.) 234–237 (Springer, 1985).
130. Ellis-Evans, J. C. & Lemon, E. C. G. Some aspects of iron cycling in maritime antarctic lakes. *Hydrobiologia* **172,** 149–164 (1989).
131. Fisher, R. V & Schmincke, H.-U. *Pyroclastic Rocks*. (Springer, 1984).
132. Marcott, S. A., Shakun, J. D., Clark, P. U. & Mix, A. C. A reconstruction of regional and global temperature for the past 11,300 years. *Science* **339,** 1198–1201 (2013).
133. Le Maitre, R. *et al. A Classification of Igneous rocks and Glossary of Terms: Recommendations of the International Union of Geological Sciences Subcommission on the Systematics of Igneous Rock*. (Blackwell Scientific Publications, 1989).
134. Irvine, T. & Baragar, W. Guide to chemical classification of common volcanic rocks. *Can. J. Earth Sci.* **8,** 523–548 (1971).
135. Kuno, H. Lateral variation of basalt magma type across continental margins and Island Arcs. *Bull. Volcanol.* **29,** 195–222 (1966).
136. Rickwood, P. C. Boundary lines within petrologic diagrams which use oxides of major and minor elements. *Lithos* **22,** 247–263 (1989).
137. Matthies, D., Mäusbacher, R. & Storzer, D. Deception Island tephra: a stratigraphical marker for limnic and marine sediments in Bransfield Strait area, Antarctica. *Zentralblatt für Geol. und Paläontologie* **1,** 153–165 (1990).
